# Supplementary material for: Large-scale crustal growth driven by LIP magmatism during the Paleoproterozoic
Source: Nat Commun. 2025 Nov 28;16:10779. doi: 10.1038/s41467-025-65826-5 (PMC12663317; doi:10.1038/s41467-025-65826-5)
Supplement: Supplementary file 1 — Description of Additional Supplementary Information [file 41467_2025_65826_MOESM1_ESM.pdf]

Description of Additional Supplementary Files

File Name: Supplementary Data 1
Description: Compilation of whole-rock Nd isotopic data from the literature used to reproduce Figures 2 and 4.

File Name: Supplementary Data 2
Description: Compilation of published arguments supporting magmatic arc and LIP models for the Amazon Craton provinces.

File Name: Supplementary Data 3
Description: Maps, field photographs, and macro- to microscopic images showing the location and petrography of the analyzed samples.

File Name: Supplementary Data 4
Description: Cathodoluminescence images of zircon grains showing analytical spots for U–Pb, trace element, and Hf isotopic analyses.

File Name: Supplementary Data 5
Description: Raw zircon U–Pb, Lu–Hf, and trace element data for samples and reference materials, including sample metadata (coordinates, stratigraphy, ages, inherited zircons), age-calculation and Lu–Hf calibration diagrams, and new whole-rock data for oxygen fugacity modelling.

File Name: Supplementary Data 6
Description: Trace-element spidergrams and rare-earth element (REE) diagrams illustrating zircon trace-element compositions of individual samples.

File Name: Supplementary Data 7
Description: Moho depth estimated from chemical models.

File Name: Supplementary Data 8
Description: Compilation of literature zircon trace-element and whole-rock geochemical data used to reproduce Figures 3 and 6.

File Name: Supplementary Data 9
Description: Compilation of literature zircon U–Pb data used to reproduce Figure 7.
