# Supplementary material for: Large-scale crustal growth driven by LIP magmatism during the Paleoproterozoic
Source: Nat Commun. 2025 Nov 28;16:10779. doi: 10.1038/s41467-025-65826-5 (PMC12663317; doi:10.1038/s41467-025-65826-5)
Supplement: Supplementary file 4 — Supplementary Data 3 [file 41467_2025_65826_MOESM4_ESM.pdf]

Supplementary Data for

Large-scale crustal growth driven by LIP magmatism during the Paleoproterozoic

Matheus S. Simões, Andrew R.C. Kylander-Clark, Marcelo L. Vasquez, Carlos A. Sommer, Lucas M.M. Rossetti, John M. Cottle, Túlio A. Mendes

Sample description and images. Coordinates are present in Supplementary Data 5

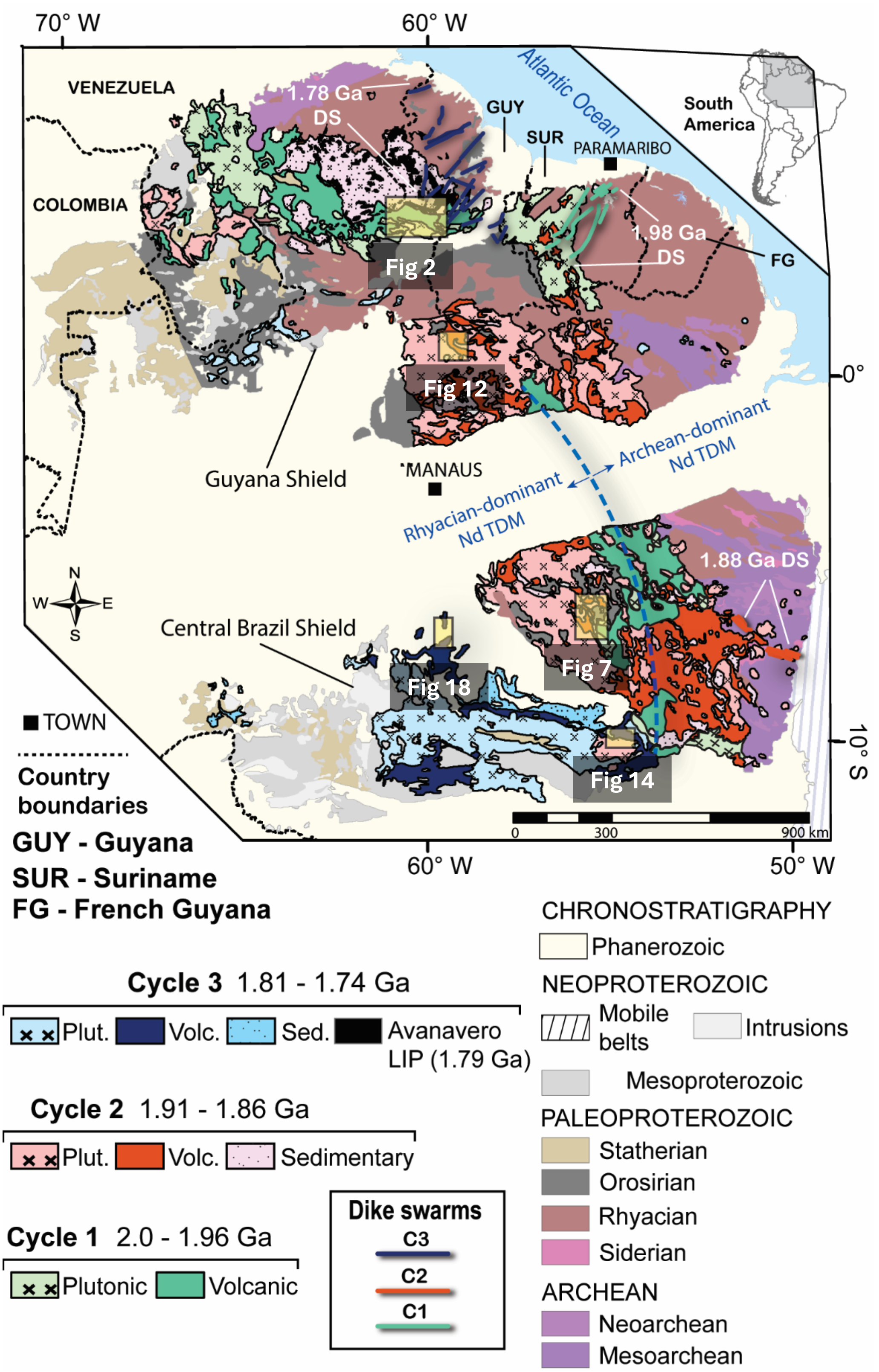

Fig 1 - Chronostratigraphic map of the Amazon Craton (excluding the southern Paraguá and Rio Apa domains). The magmatic cycles indicated in the legend are interpreted in this work. Yellow transparent rectangles indicate the area of the following geological maps. Modified from [1]

Cycle 1

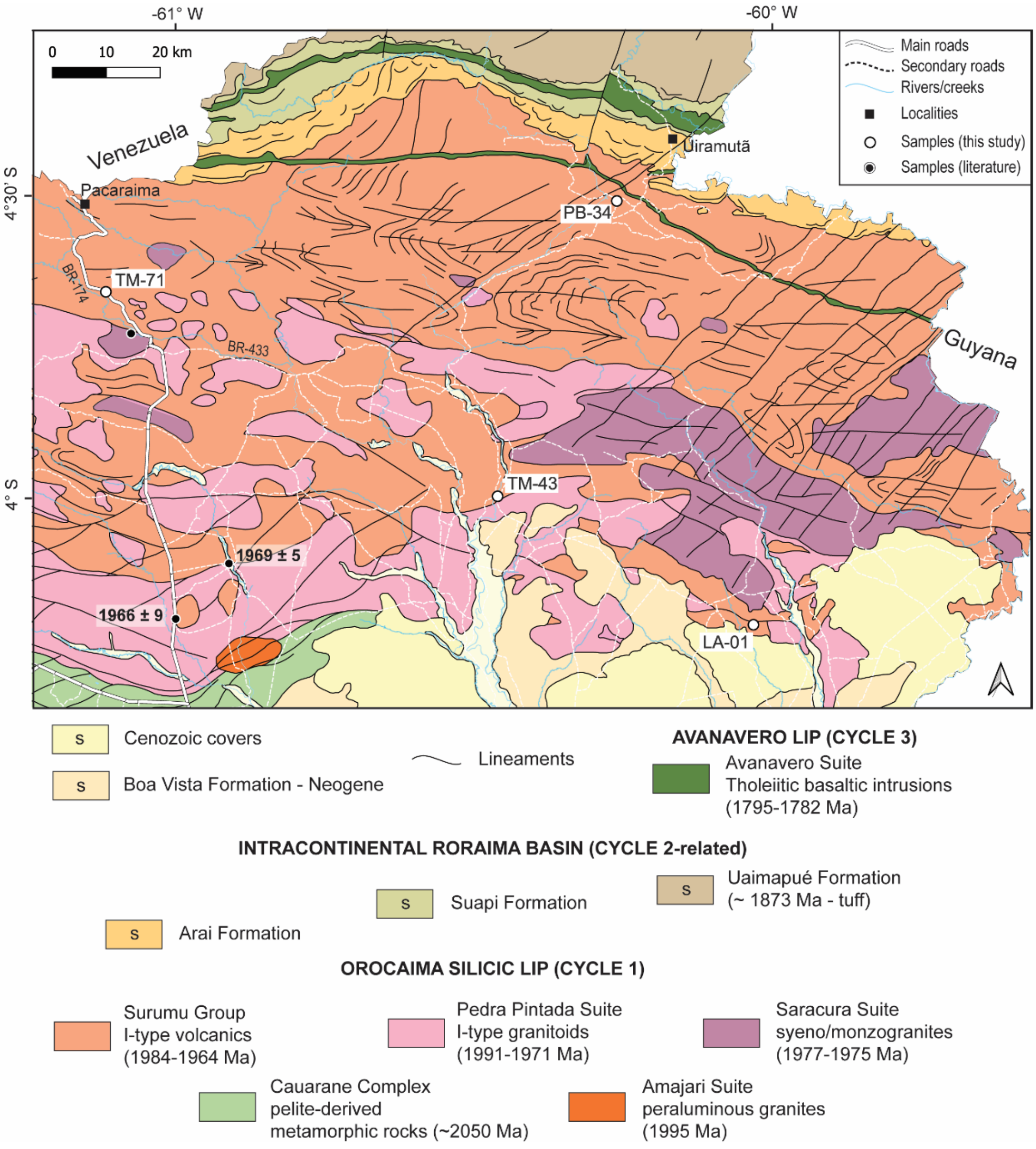

Fig 2 - Geological map with Cycle 1 sample location from this study and the literature in the Guyana Shield. (s) is for sedimentary units. Modified from [2].

TM-43 - Surumu Group

Location: Pinnacle-shaped outcrops at the Northern margin of the BR-433 road, 27.2 km south to the road interchange with BR-171 (A, B).

Field features: Dark grey porphyritic dacite with plagioclase phenocrysts and aphanitic groundmass (C). Metamorphic-mylonitic foliation parallel to the pinnacles. Down-dip lineation.

Description: Foliated and porphyritic grey dacitic ignimbrite (D) with (1) 20-30% of anhedral to subhedral tabular plagioclase phenocrysts (0.5 – 3 mm of the major axis) with albite-pericline twinning (E), partially replaced by epidote, chlorite and white mica. Locally in glomeroporphyritic texture; (2) anhedral to subhedral amoeboid Fe-Ti oxides up to 1 mm with margins replaced by leucoxene, locally in glomeroporphytitic texture. The groundmass is quartz-feldspathic with chlorite and white mica in lepidoblastic texture.

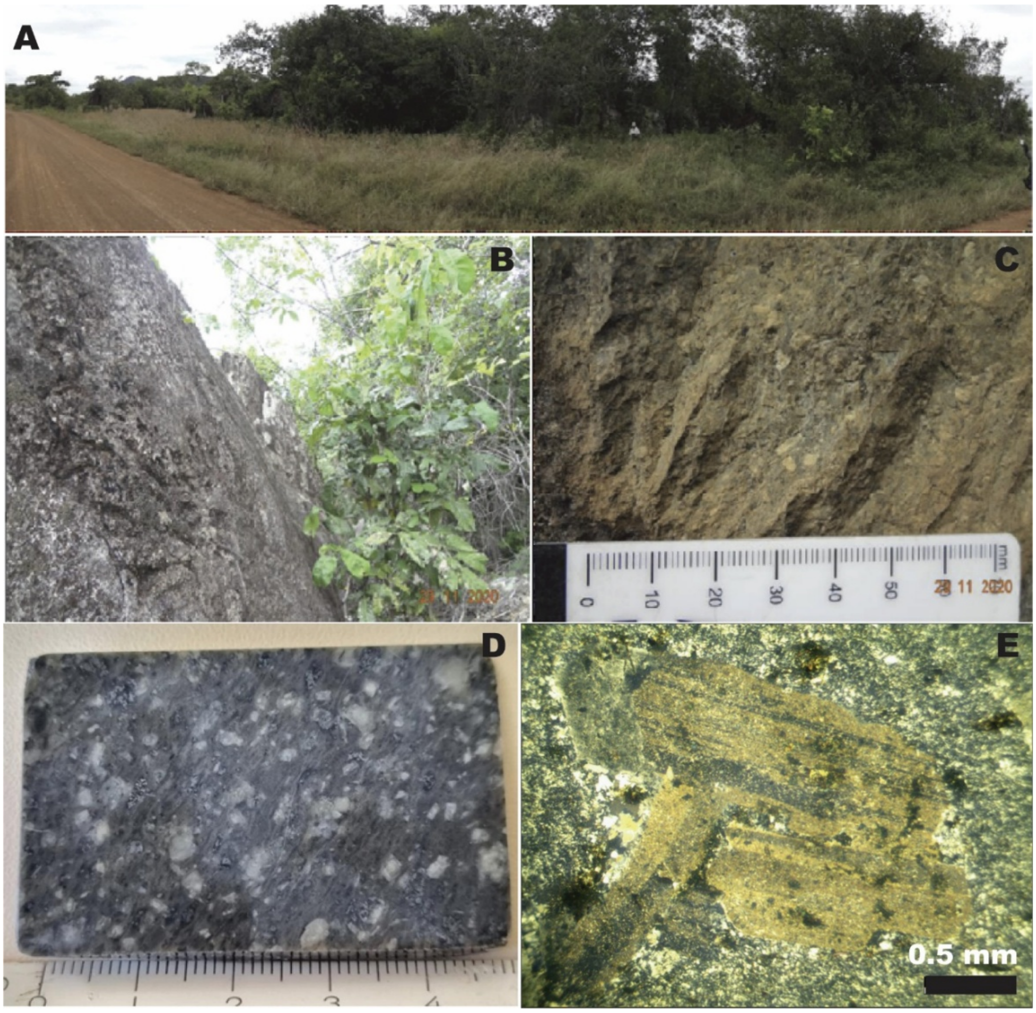

Fig 3 - Field, macroscopic and microscopic features of sample TM-43. (A) View of the outcrop location. (B) View of the outcrop dimensions. (C) Altered porphyritic volcanic rock. (D) Foliated porphyritic volcanic rock. (E) Plagioclase phenocrysts immersed in a microcrystalline groundmass.

TM-71 - Surumu Group

Location: Pinnacle-shaped outcrop at the western margin of the BR-171 (A).

Field features: Greyish porphyritic meta-tuff (B) with metamorphic foliation and 30-40% of plagioclase and mafic phenocrysts.

Description: Whitish, greyish porphyritic meta-latitic ignimbrite (C) with (1) 40% of anhedral, subhedral, tabular and broken perthite phenocrysts (0.5 – 3 mm, major axis) moderately altered to white mica (D), displaying corrosion gulfs and partially corroded nuclei; (2) Anhedral to subhedral prismatic plagioclase (0.5 – 2.5 mm) partially chloritized; (3) Green chlorite and leucoxene-bearing aggregates (0.5 – 4 mm); (4) anhedral amoeboid to globular solitary Fe-Ti oxides sometimes in within chlorite + perthite + zircon aggregates. The groundmass if quartz-feldspathic with opaque minerals and chlorite.

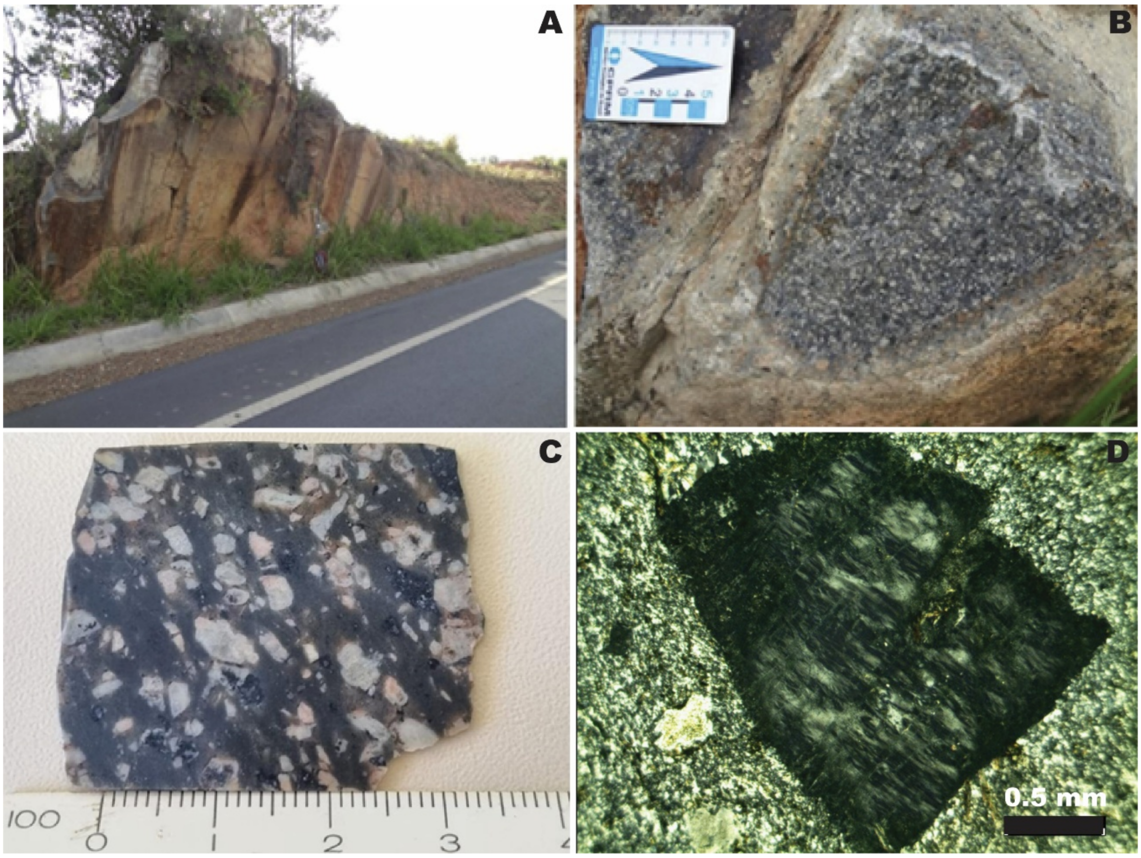

Fig 4 - Field, macroscopic and microscopic features of sample TM-71. (A) View of the outcrop location and dimensions. (B) Altered porphyritic volcanic rock. (C) Slightly foliated porphyritic volcanic rock with feldspar and mafic mineral phenocrysts. (E) Perthite phenocrysts immersed in a microcrystalline groundmass.

LA-01 – Cachoeira da Ilha Formation

Location: Hill close to the extraction sector 1 of the Atola range (A)

Field features: Greyish to greenish foliated meta-tuff (B).

Description: Foliated rock with 7% of porphyroclasts of (1) euhedral to anhedral prismatic perthite (0.8 – 4 mm, major axis), variably zoned, altered to white mica and containing apatite and Fe-Ti oxide inclusions; (2) subhedral fractured laths of plagioclase (0.4 – 2 mm, major axis), with apatite and Fe-Ti oxide inclusions©; (3) sector zoned anhedral quartz (0.5 mm); (4) cubic to globular euhedral to subhedral Fe-Ti oxides (<0.1 mm – 0.5 mm) isolated or in glomeroporphyritic texture, locally associated with biotite. The groundmass is composed of recrystallized K-feldspar, plagioclase, quartz and biotite with < 0.1 to 0.5 mm. The rock framework is oriented, showing foliation marked by biotite orientation and stretched quartz with pressure shadows (D). Accessory phases are apatite, zircon and hematite.

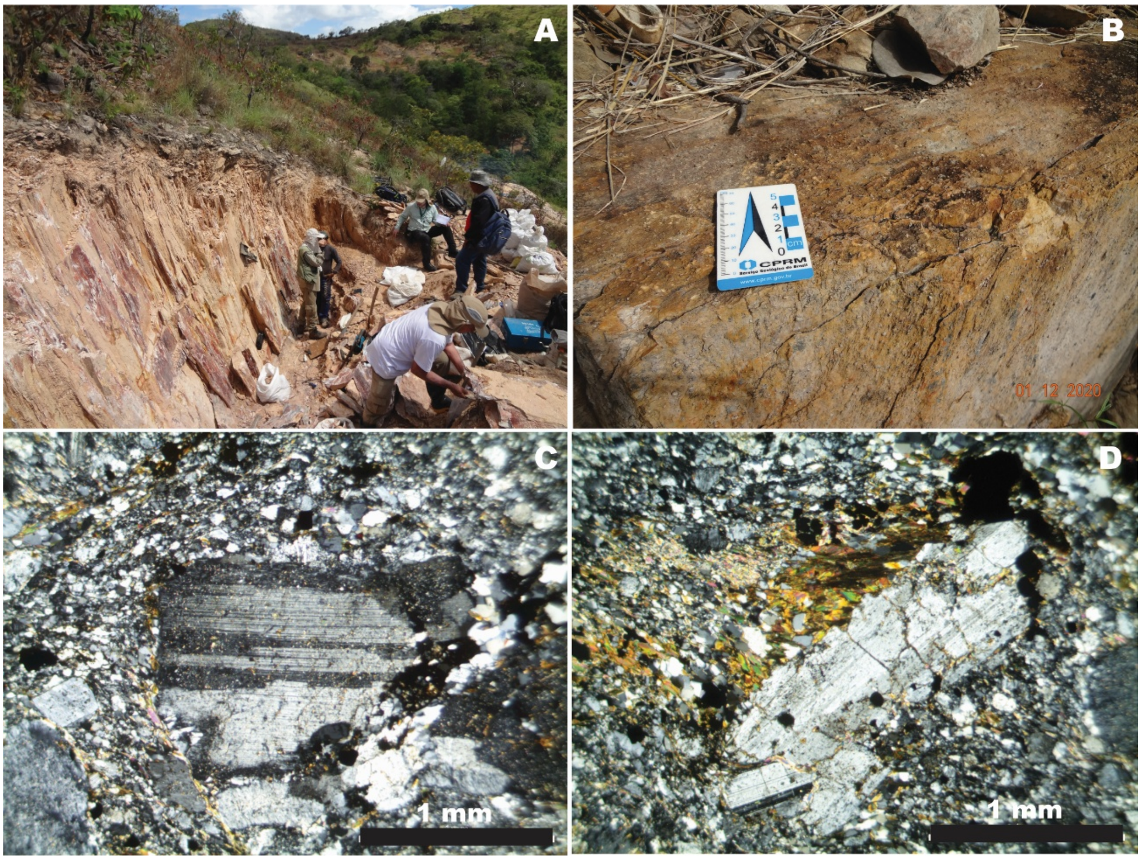

Fig 5 - Field, macroscopic and microscopic features of sample LA-01. (A,B) View of the outcrop location and dimensions. (C) Plagioclase porphyroclast immersed in microcrystalline groundmass. (D) Pressure shadow filled by biotite associated to plagioclase porphyroclast.

PB-34 – Surumu Group

Location: Extensive outcrop along the Socó creek close to BR-171 road.

Field features: Greyish foliated porphyritic rock (A).

Description: Porphyritic ignimbrite (B) with plagioclase phenocrysts (1 mm, major axis) and aphanitic groundmass. Columnar joints (A) and entablature occur with joints elongated in the ESE-WNE trend. The horizontal bedding is cut by metamorphic foliation.

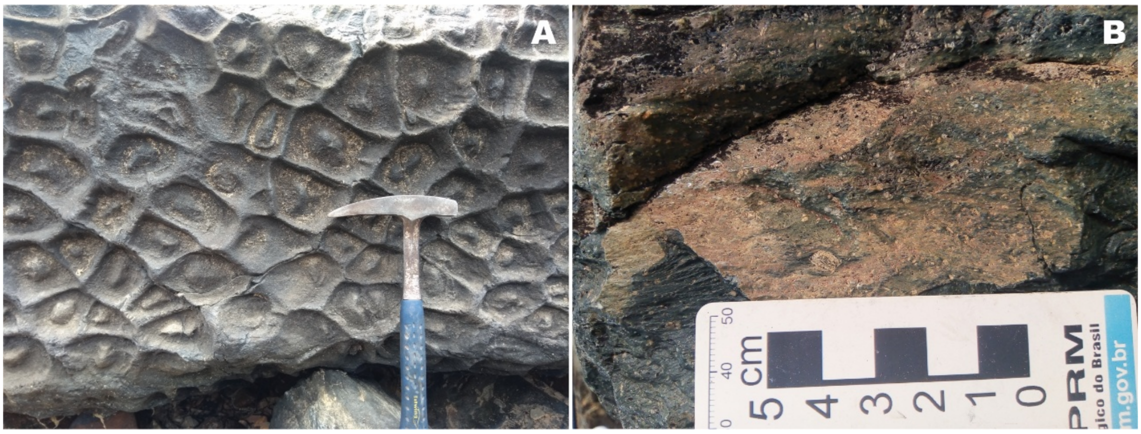

Fig 6 - Field and macroscopic features of sample PB-34. (A) Columnar joints in the outcrop. (B) Altered porphyritic volcanic rock.

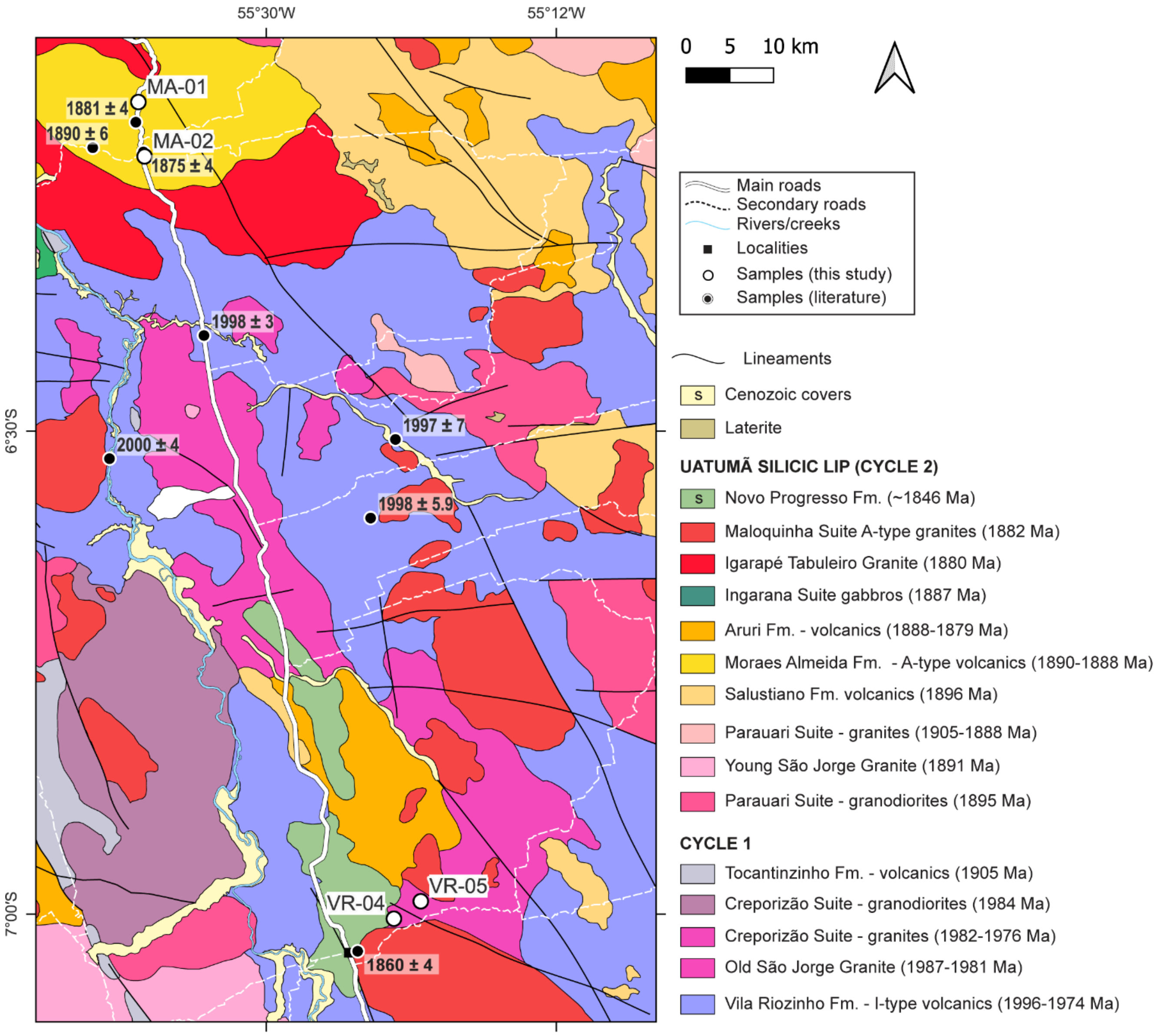

Fig 7 - Geological map with Cycle 1 and 2 sample location from this study and the literature in the Central Brazil Shield. (s) is for sedimentary units. Modified from [3].

VR-04 – Vila Riozinho Formation

Location: Boulders in the margin of the Celeste secondary road (A).

Field features: Porphyritic volcanic rock (B).

Description: Rhyolitic lapilli tuff with porphyritic texture, showing 10-15% of phenocrysts composed of (1) anhedral to subhedral prismatic perthite, with 0.2 – 5 mm, which are broken (C, D), contain chloritized biotite and quartz inclusions and is altered to white mica. Smaller grains are globular and bigger grains have embayment; (2) subhedral do anhedral prismatic to globular quartz (0.4 mm), sometimes broken and with embayments; (3) subhedral to anhedral cubic to globular oxides (0.1 – 0.5 mm). This rock also contains lithic fragments (1 – 10 mm) compositionally resembling the population of phenocrysts (perthite, quarz and oxides). The groundmass is microcrystalline with quartz, feldspars and oxides with elongated, up to 3 mm, structures filled with epitaxial quartz, feldspar and submilimetric oxide aggregates.

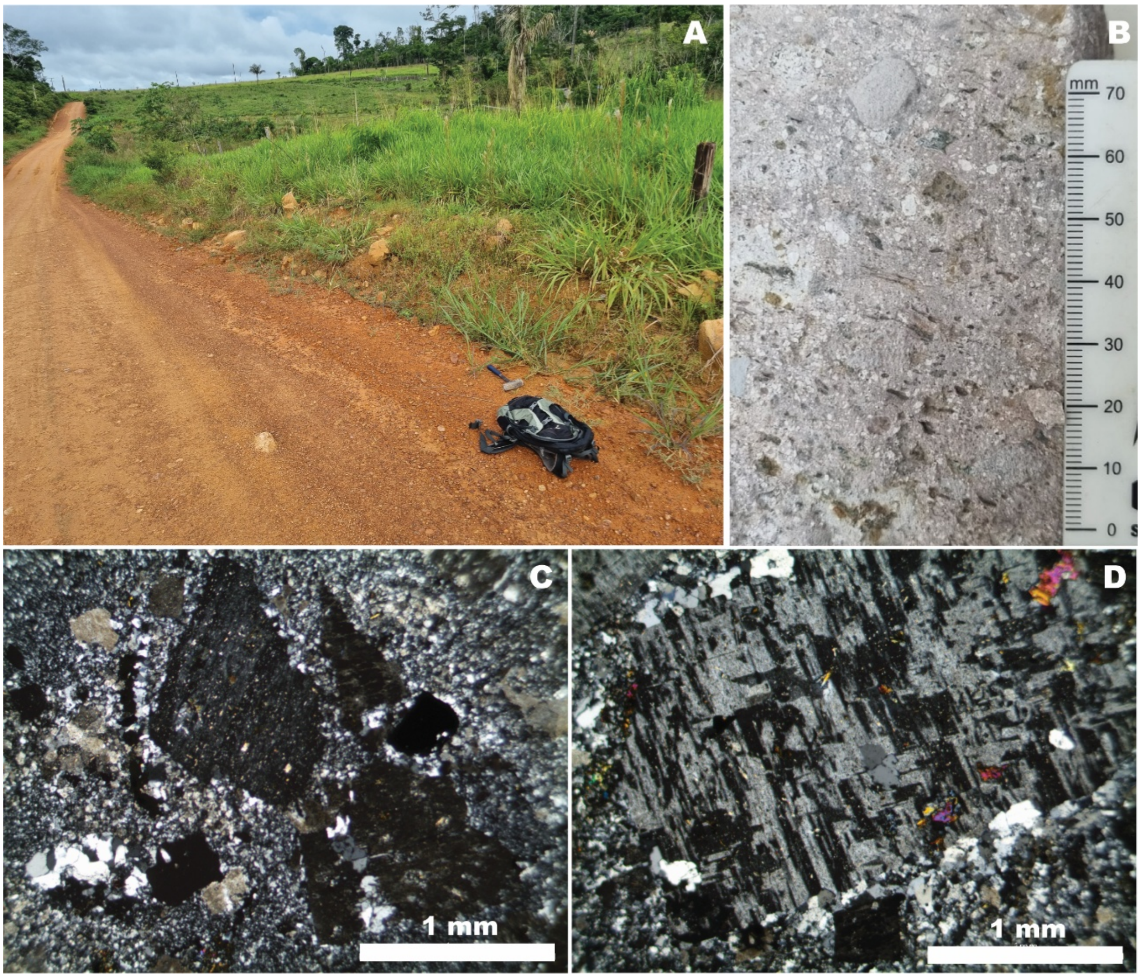

Fig 8 - Field, macroscopic and microscopic features of sample LA-01. (A) View of the outcrop location and dimensions. (B) Fragments within a vitroclastic groundmass. (C) General texture of broken phenocrysts in microcrystalline groundmass. (D) Perthite phenocryst.

VR-05 – Vila Riozinho Formation

Location: Boulders in the margin of the Celeste secondary road (A).

Field features: Porphyritic volcanic rock (B).

Description: Trachytic lapilli tuff with porphyritic texture, showing 35% of phenocrysts composed of (1) subhedral prismatic and broken perthite (0.2 – 2 mm) which is concentrically zoned, with corroded or recrystallized margins (C). Altered to epidote and white mica. (2) subhedral prismatic and broken plagioclase (0.4 – 1.2 mm), exhibiting sector zoning, locally with corroded magins and cores (D), contains oxide inclusions and is altered do epidote and white mica (3) Cubic to orthorombic oxides (~0.4 mm) with melt inclusions, associated with zircon. Cognate lithics are presente with plagioclase + orhoclase + pertite + plagioclase + quartz + oxides. Elongated fragments with chlorite-rich and quartz feldspathic bands occur. The rock is cut by quartz veins causing alteration to white mica and opaque mineral precipitation.

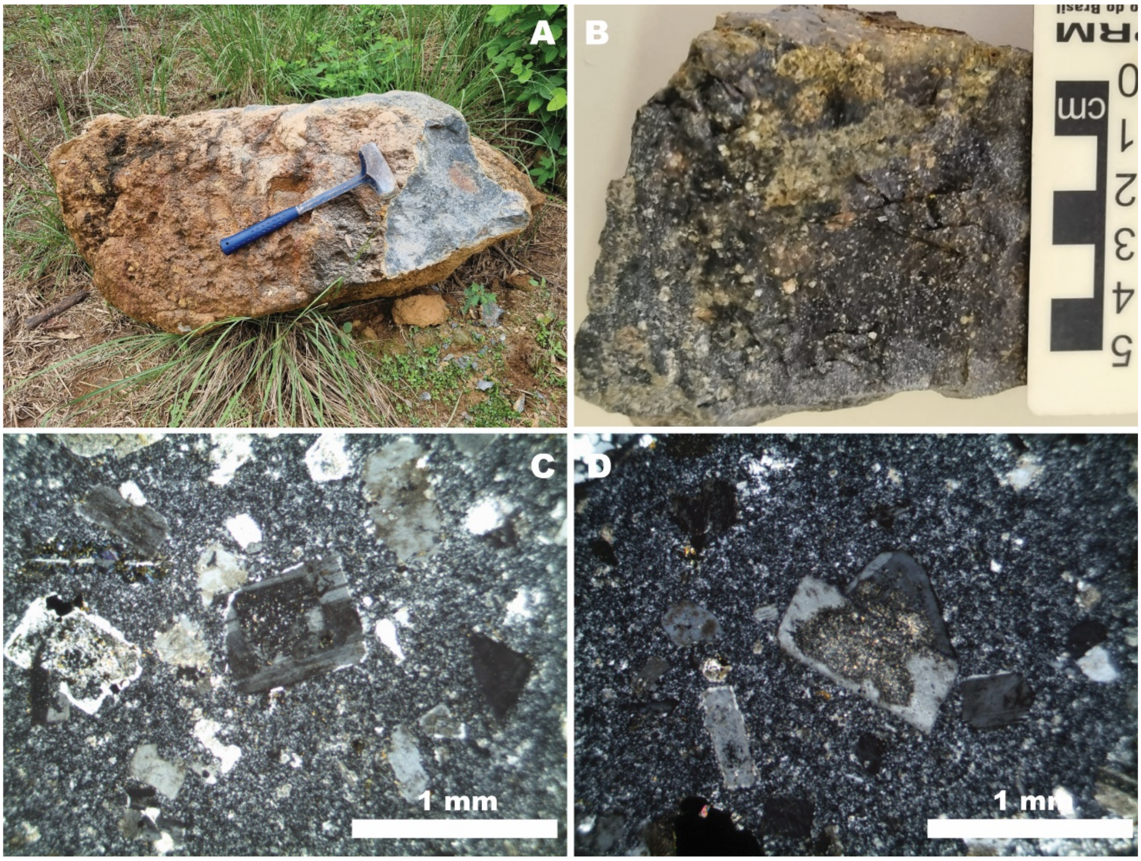

Fig 9 - Field, macroscopic and microscopic features of sample LA-01. (A) View of the outcrop location and dimensions. (B) Porphyritic volcanic rock. (C) General texture of broken phenocrysts in microcrystalline groundmass. (D) Plagioclase phenocryst with corroded nuclei.

Cycle 2

MA-01 – Moraes Almeida Formation

Location: Road-cut at BR-163 road, close to Moraes Almeida town (A).

Field features: Color-banded igneous (B, C) rock enriched in feldspar, quartz and biotite crystals. Banding is parallel to a regional WNW-ESE mountain range trend.

Description: Porphyritic rhyolite with ~ 45% phenocrysts of (1) subhedral tabular perthite (0.2 – 5 mm, major axis), locally zoned and pervasively corroded in margins and nuclei. Opaque, serpentinized mafics, apatite and amphibole inclusions; (2) subhedral tabular corroded plagioclase (0.2 – 2 mm) (D). Corrosion occurs in margins and cores along with resorption textures and concentric zoning. Opaque and amphibole inclusions; (3) subhedral to anhedral prismatic quartz (0.1 – 4 mm), locally broken and with resorption textures. Melt inclusions also occur. At the margins there are reaction textures between quartz and groundmass, internally the grains may present graphic textures; (4) subhedral prismatic clinopyroxene (~ 1.8 mm) with blue, pink and yellow interference colors (E) displaying margins transformed to green amphibole (uralite); (5) Anhedral prismatic green amphibole (<0.4 mm) with opaque and biotite inclusions, occurring altered and with resorption textures; (6) subhedral to anhedral brown biotite (<0.4 mm) with opaque inclusions and generally resorbed; (7) subhedral rhombohedral opaques (< 0.5 mm) mantled by biotite as isolated crystals or in glomeroporphyritic texture. The groundmass is fine-grained (< 0.1 mm grains) and composed of quartz, K-feldspar, plagioclase, amphibole, biotite and opaques.

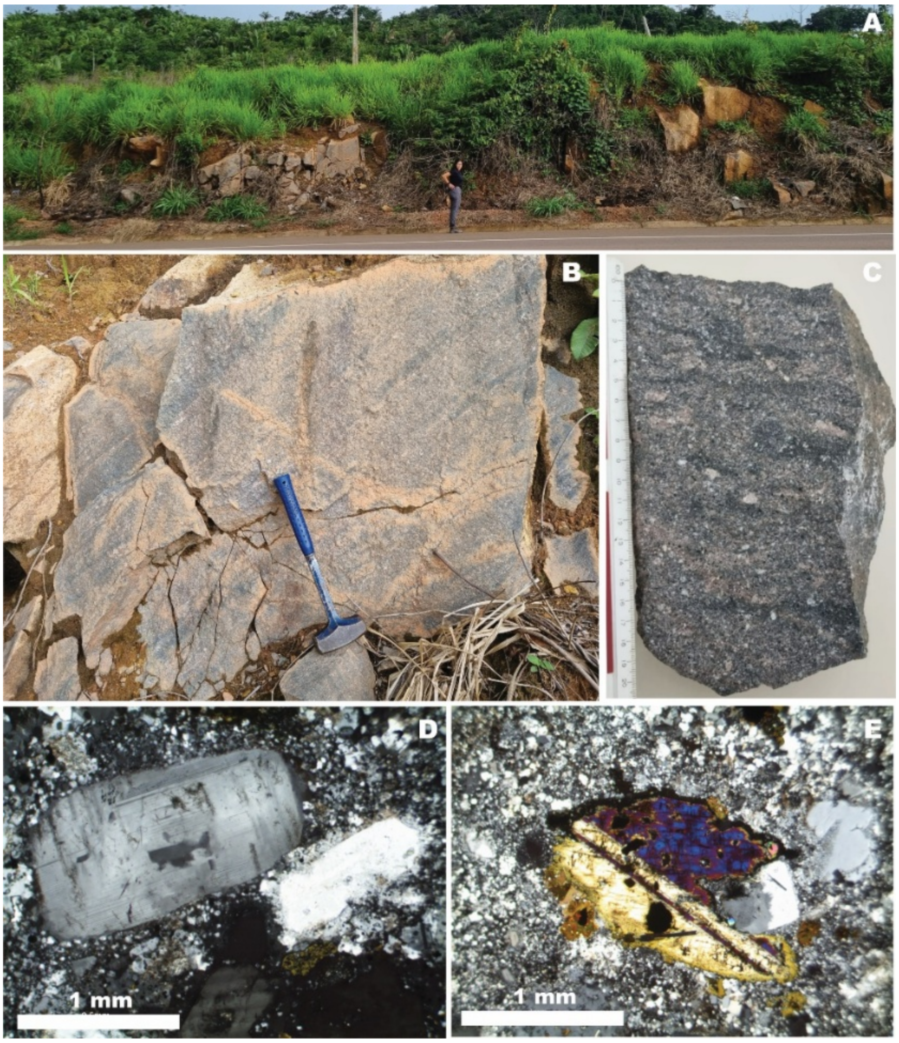

Fig 10 - Field, macroscopic and microscopic features of sample MA-01. (A) View of the outcrop location and dimensions. (B) Irregular color banding. (C) Banded porphyritic volcanic rock. (D) Plagioclase and quartz phenocrysts immersed in a fine-grained groundmass. (E) Clinopyroxene with twinning immersed in a fine-grained groundmass.

MA-02 – Moraes Almeida Formation

Location: Boulders in the eastern margin of BR-163 road, close to a gas station (A).

Field features: Reddish to brownish crystal-rich ignimbrite (B) with rapakivi texture in feldspar phenocrysts, globular quartz and small mafic minerals.

Description: Crystal-rich ignimbrite (C) with phenocrysts and crystal clasts of: (1) subhedral to euhedral prismatic quartz (0.3 – 2.2 mm), generally broken and rarely anhedral amoeboid. It occurs partially resorbed, with corrosion gulfs and melt inclusions; (2) subhedral to to anhedral prismatic perthite (0.7 – 2.1 mm), with opaque inclusions, generally broken. It occurs with concentric zoning, locally with corrosion gulfs and melt inclusions. Partially altered to white mica; (3) subhedral prismatic plagioclase (0.3 – 1 mm) as isolated or glomeroporphyritic phases, pervasively altered to white mica; (4) prismatic pyroxene (0.3 – 1.5 mm) pervasively altered to hematite and serpentine, with opaque inclusions; (5) subhedral rhombohedral opaques (0.2 – 0.8 mm) as isolated phases or in glomeroporphyritic texture, altered to hematite; (6) 'possible' subhedral prismatic fayalite (0.6 mm) intensely fractured and replaced by hematite and serpentine. The groundmass is microcrystalline, quartz-feldspathic, with angulous and broken quartz and feldspar clasts. It also shows shards and contorted fiammé enveloping crystals.

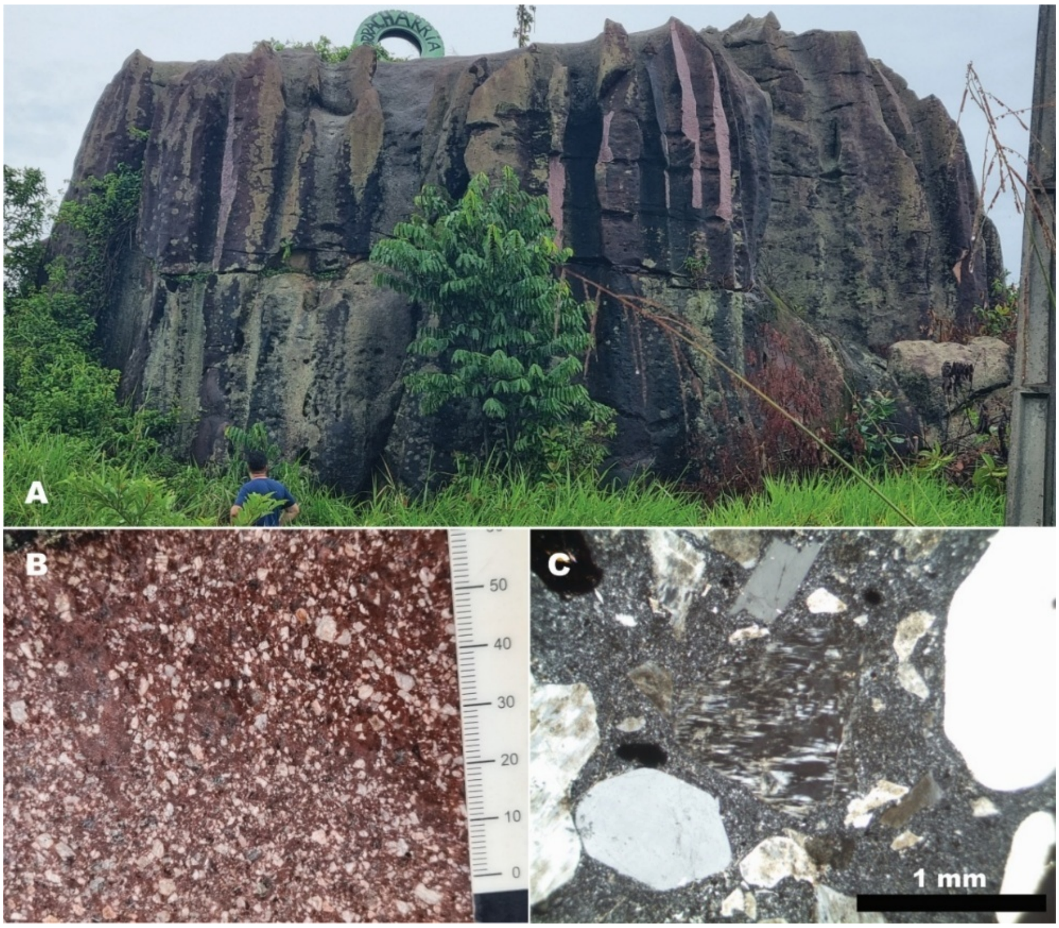

Fig 11 - Field, macroscopic and microscopic features of sample MA-02. (A) View of the outcrop location and dimensions. (B) Crystal-rich ignimbrite. (C) Perthite and quartz phenocrysts and crystal clasts immersed in a microcrystalline groundmass.

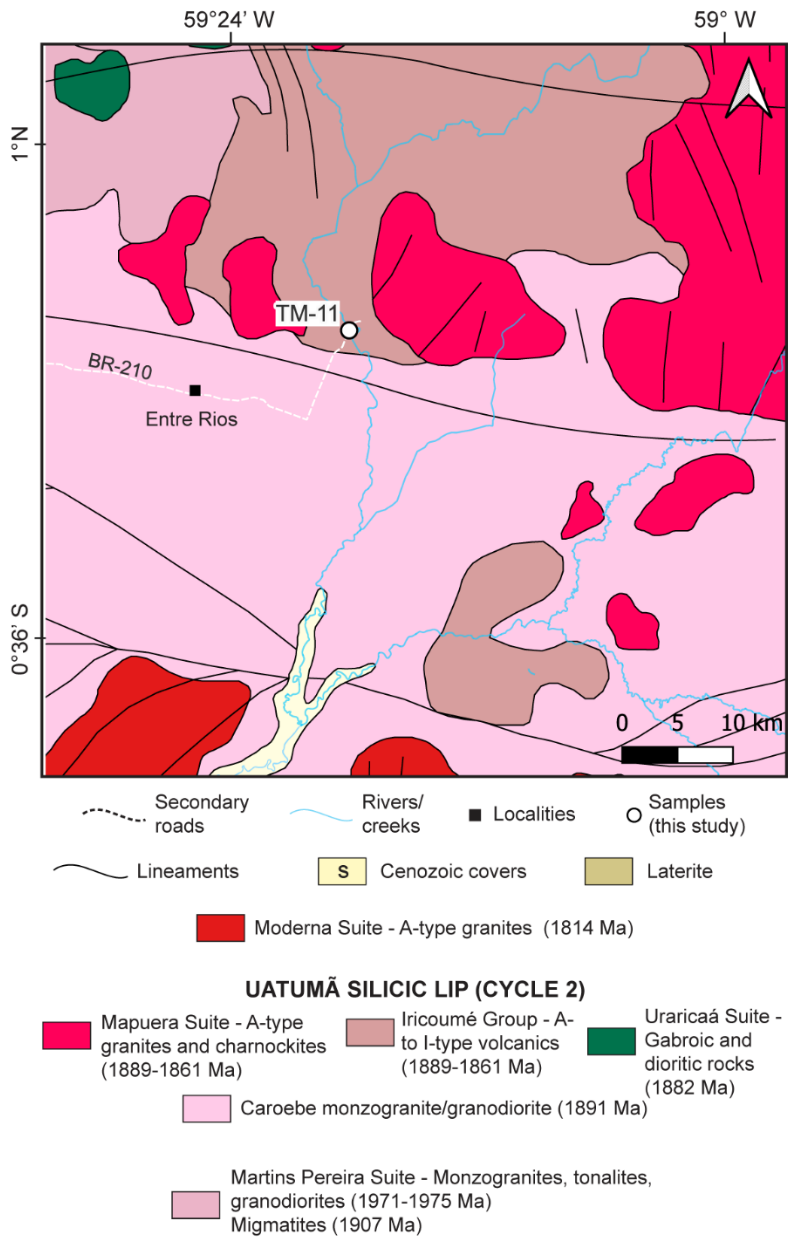

Fig 12 - Geological map with Cycle 2 sample location from this study. (s) is for sedimentary units. Modified from [2].

TM-11 – Jatapu Formation

Location: Under the bridge of a secondary road to BR-210 along the Jatapu river.

Field features: Porphyritic volcanic rock with vertical foliation.

Description: Porphyritic dacite with ~ 40% phenocrysts of (1) euhedral to subhedral tabular plagioclase (0.5 – 5 mm) with albite-pericline twinning, pervasively altered to epidote and white mica. They occur isolated or in glomeroporphyritic texture, forming 5 – 6 mm aggregates. Some broken and anhedral crystals also occur; (2) globular to cubic Fe-Ti oxides with rounded edges (<0.5 – 1 mm), locally in glomeroporphyritic texture; (3) subhedral prismatic mafic mineral relicts (0.1 – 2 mm) replaced by microgranular opaques, chlorite and epidote; (4) subhedral mafic relicts (1 mm) totally replaced by chlorite, epidote and carbonate; (5) anhedral amoeboid

quartz (1 mm). The groundmass is composed of tabular plagioclase, globular opaque and epidote microlites along with euhedral apatite.

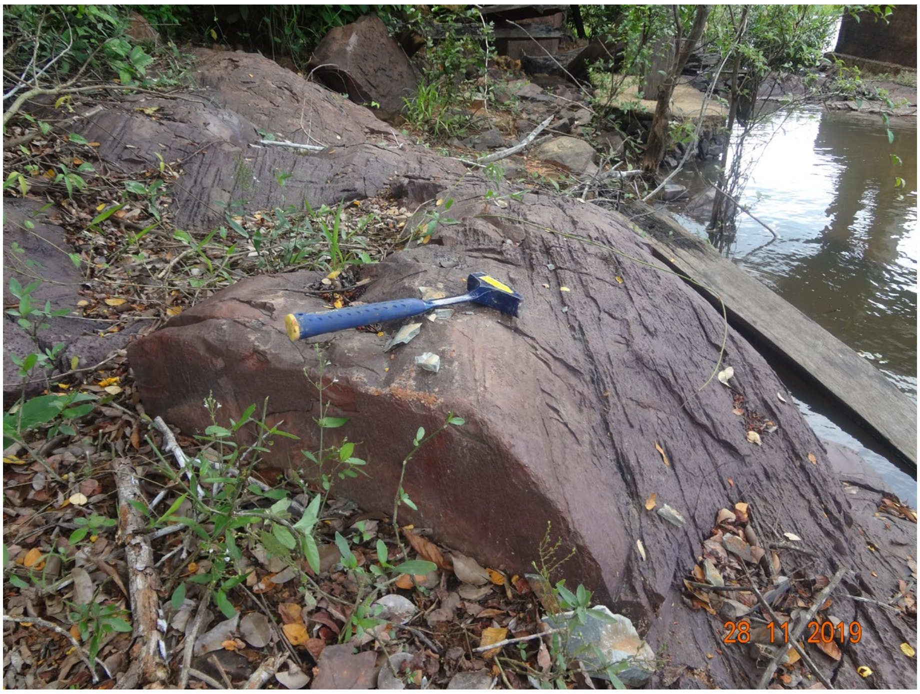

Fig 13 - Field, macroscopic and microscopic features of sample TM-11. Foliated dacite.

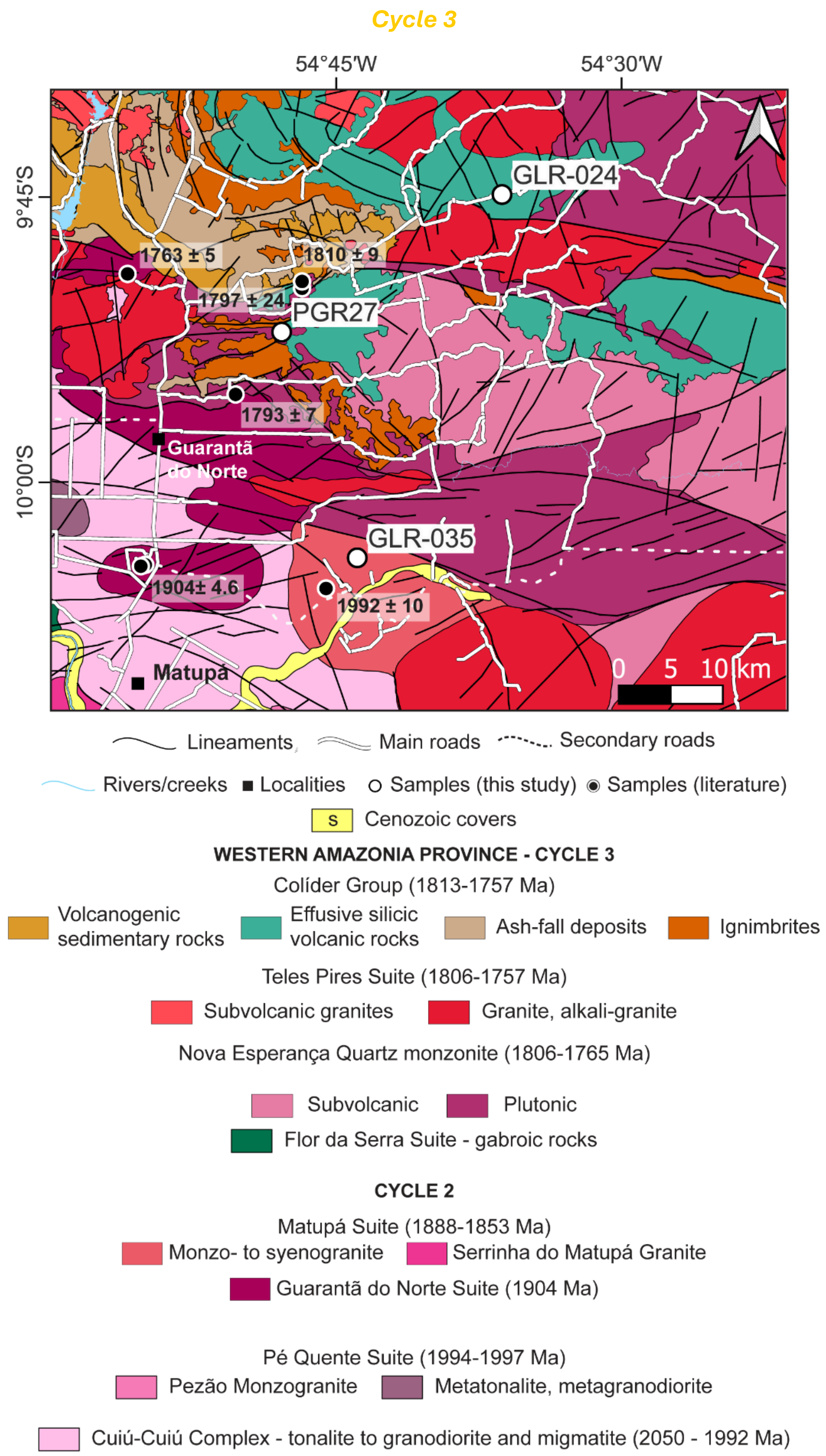

Fig 14 - Geological map with Cycle 3 sample location from this study. (s) is for sedimentary units. Modified from [4].

GLR-035 - Colíder Group

Location: Quarry on a secondary road to the east of BR-163.

Field features: Massive brownish volcanic rock with few phenocrysts and fine-grained groundmass (A).

Description: Rhyolitic lapilli tuff with 10-15% of phenocrysts of: (1) euhedral to subhedral tabular perthite (major axis with 0.5 – 2.5 mm) with corroded margins, with apatite, opaque and zircon inclusion, altered to white mica and associated with microcline (B); (2) subhedral, generally chloritized, biotite (0.3 – 1.2 mm) with leucoxene and hematite between lamellae, exhibiting opaque and zircon inclusions; (3) anhedral globular to slightly elongated quartz (0.3 – 0.8 mm) with corrosion gulfs and corroded margins and slightly undulating extinction; (4) subhedral to anhedral globular to cubic opaques (0.2 – 0.5 mm) with apatite inclusions; (5) euhedral prismatic apatite (0.4 mm). The rock presents lithic fragments (1 – 4 mm) of allotriomorphic textured equigranular quartz-feldspathic rocks, locally with porphyritic texture with the same mineralogy of the phenocrysts. The groundmass is quartz-feldspathic microcrystalline with oxides, apatite and zircon, displaying relicts of vitroclastic textures.

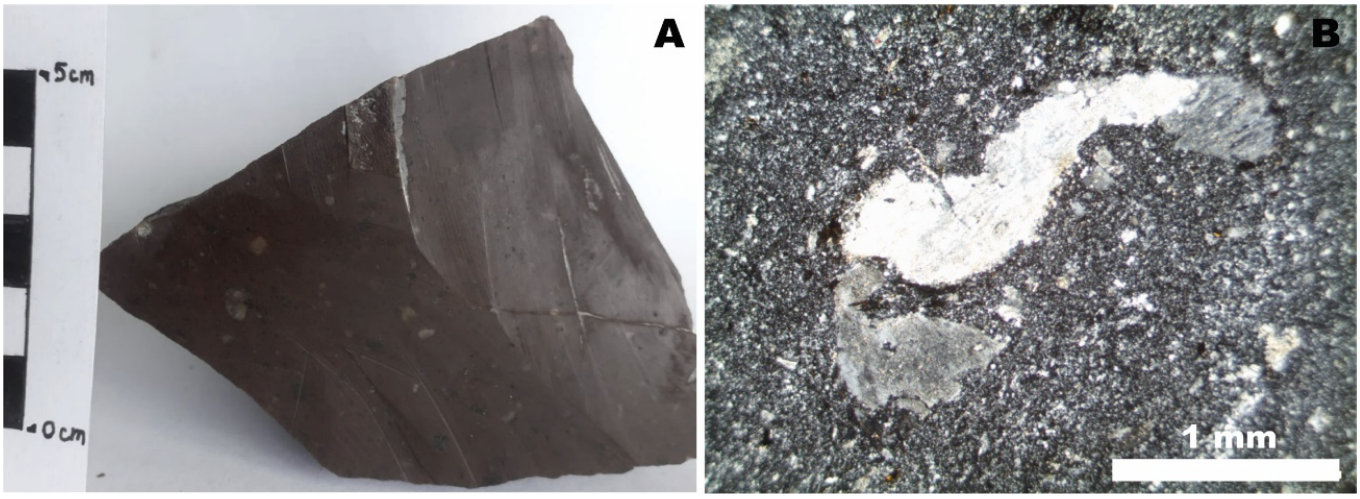

Fig 15 - Macroscopic and microscopic features of sample GLR-035. (A) Slightly porphyritic volcanic rock. (B) Quartz and feldspar phenocrysts within a microcrystalline groundmass.

GLR-024 - Colíder Group

Location: Boulders on a secondary road to the east of BR-163.

Field features: Boulders of porphyritic rhyolite.

Description: Porphyritic rhyolite (A) with 3% of phenocrysts of: (1) subhedral perthite (0.5 – 3 mm) with opaque inclusions and resorption features in margins and nuclei along with corrosion gulfs, generally oxidized and altered to white mica; (2) subhedral prismatic feldspars in glomeroporphyritic texture, pervasively altered to white mica, smectite and hematite; (3) subhedral lamellar biotite (0.6 mm) altered to chlorite and carbonate associated with zircon; (4) anhedral and subhedral globular to cubic opaques (0.2 – 0.4 mm). The groundmass has alotriomorphic texture (B) and is composed of quartz, K-feldspar, plagioclase, opaques and biotite, locally presenting myrmekite textures.

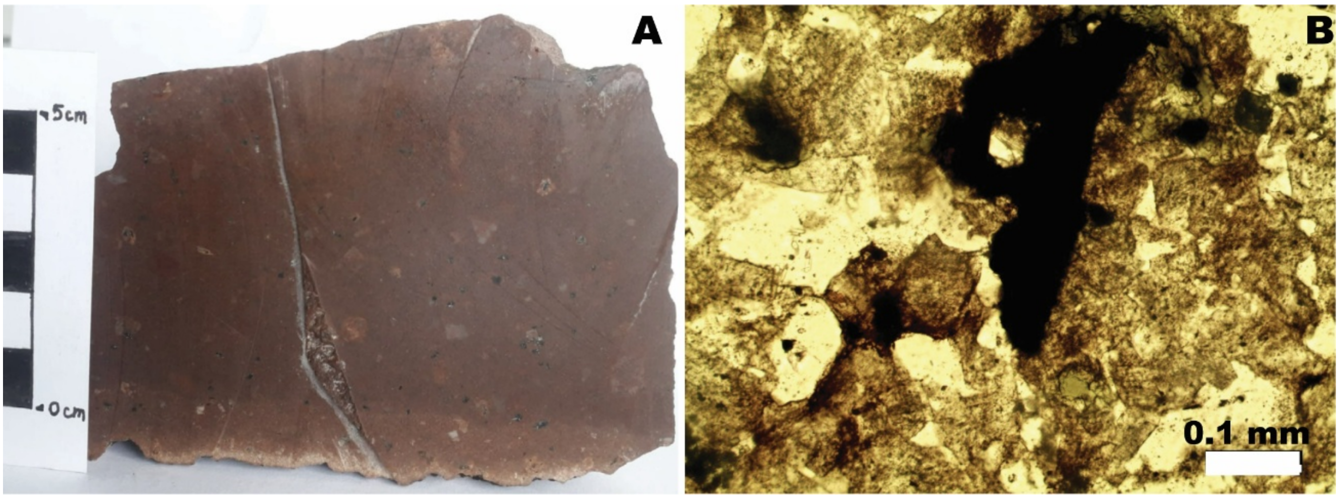

Fig 16 - Macroscopic and microscopic features of sample GLR-035. (A) Slightly porphyritic volcanic rock. (B) Crystal clasts within a microcrystalline groundmass.

PGR-27 - Colíder Group

Location: Boulders on a secondary road to the east of BR-163.

Field features: Boulders of slightly foliated volcanic rock.

Description: Porphyritic rhyolitic lapilli tuff (A) with 35% of fragments including phenocrysts of (1) subhedral to anhedral prismatic broken perthite (0.3 – 2 mm) altered to white mica with corroded margins, isolated or in glomeropophyritic texture, with opaque, apatite, zircon and biotite inclusions; (2) anhedral broken plagioclase (0.3 – 3 mm) with albite/albite pericline twinning, resorbed margins, isolated or in glomeroporphyritic texture; (3) euhedral to subhedral prismatic clinopyroxene (0.2 – 0.5 mm) as entire or broken crystals pervasively altered to hematite and carbonate; (4) subhedral lamellar biotite (~0.4 mm) generally chloritized and altered to hematite and white mica; (5) anhedral broken amoeboid quartz (0.2 – 0.4 mm) with corroded margins and slightly undulating extinction; (6) subhedral cubic to globular oxides (0.2 – 0.4 mm) isolated or in glomeroporphyritic texture. In places, they are broken and with margins altered to leucoxene and carbonate and contain zircon and clinopyroxene inclusions. Lithic fragments are composed of (A) Laminated siltstone (5 mm); (B) quartz-feldspathic fine-grained well-sorted sandstone; (C) Subvolcanic quartz-feldspathic fine-grained allotriomorphic rock (cognate?); (D) porphyritic subvolcanic rock; (E) medium-grained quartz-feldspathic sandstone; (F) subvolcanic rhyolite with biotite and opaques (cognate?).

The groundmass is poorly-sorted with fragments of quartz, feldspars, opaques (all < 0.2 mm), showing fine discontinuous banding enveloping the phenocrysts, interpreted as vitroclastic texture (B).

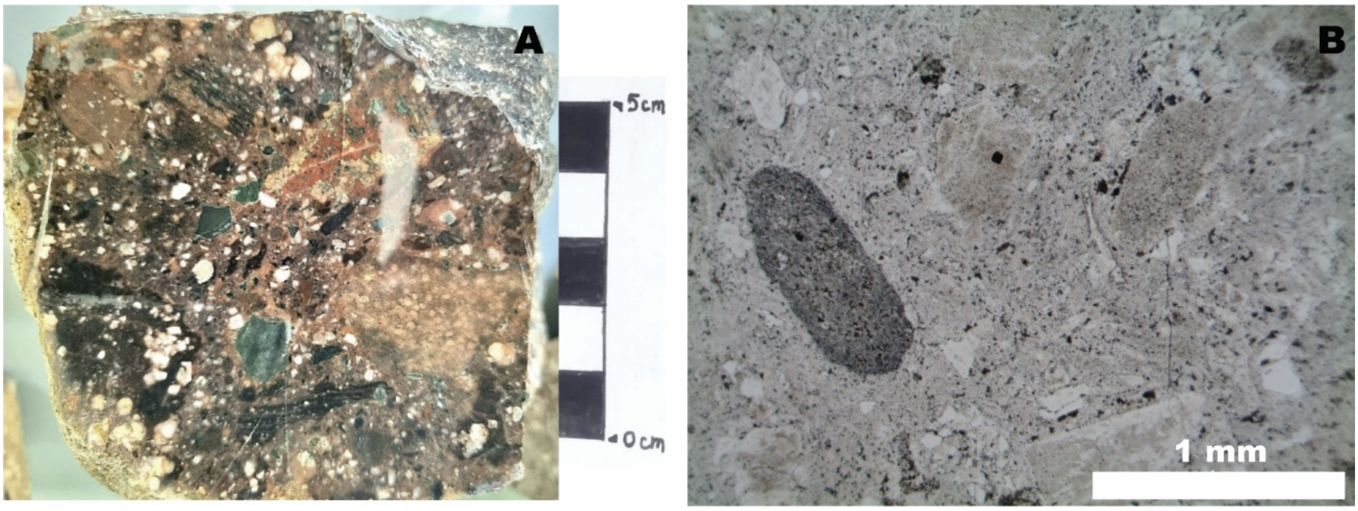

Fig 17 - Macroscopic and microscopic features of sample PGR-27. (A) Crystal and lithic-rich ignimbrite with vitroclastic clasts. (B) Dark rounded siltstone lithic and vitroclastic shard-rich groundmass.

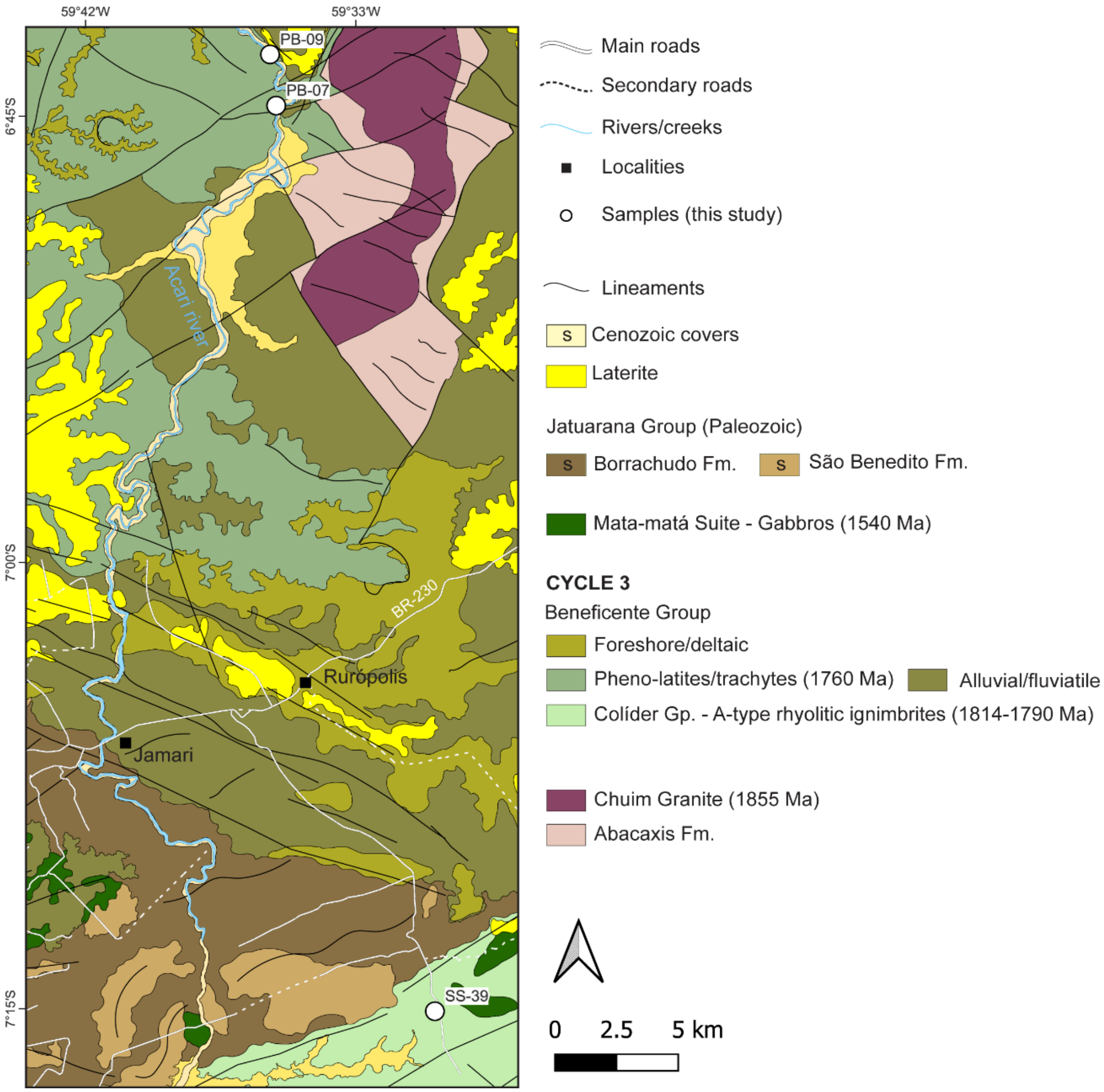

Fig 18 - Geological map with Cycle 3 sample location from this study. (s) is for sedimentary units. Modified from [5].

SS-39 - Colíder Group

Location: Três Estados secondary road to BR-230.

Field features: Loose blocks and boulders of slightly foliated and porphyritic pinkish porphyritic rhyolite (A).

Description: Crystal-rich rhyolitic fine- to medium-grained ignimbrite. Crystal clasts are composed of (1) euhedral to subhedral quartz as hexagonal prisms or angular fragments (0.5 – 5 mm) presenting slight undulating extinction, resorption features, melt inclusions and broken apart textures. Low to medium recrystallization degree and concentration of chlorite at the margins with the groundmass (B); (2) subhedral to anhedral prismatic K-feldspar (0.6 – 5 mm) with mesoperthite, resorption features, broken apart textures and melt inclusions; (3) anhedral to subhedral rhombohedral opaques (0.2 – 0.6 mm) exhibiting titanite exsolution and margins with to epidote and chlorite; (4) anhedral plagioclase as angular fragments (~2 mm) with polysynthetic albite-pericline twinning, partially recrystallized along the margins; (5) prismatic zircon with concentric zoning (~0.5 mm) associated with opaques; (6) amphibole pseudomorphs as chlorite+epidote+opaque aggregates. The rock also contains lithoclasts (~0,2 mm) as subrounded fragments composed of quartz, plagioclase, K-feldspar, opaques and apatite. Alteration minerals are from chlorite group (with Berlim blue interference color), epidote and white mica. The groundmass is microcrystalline, quartz-feldspathic and is recrystallized near contacts with crystal clasts.

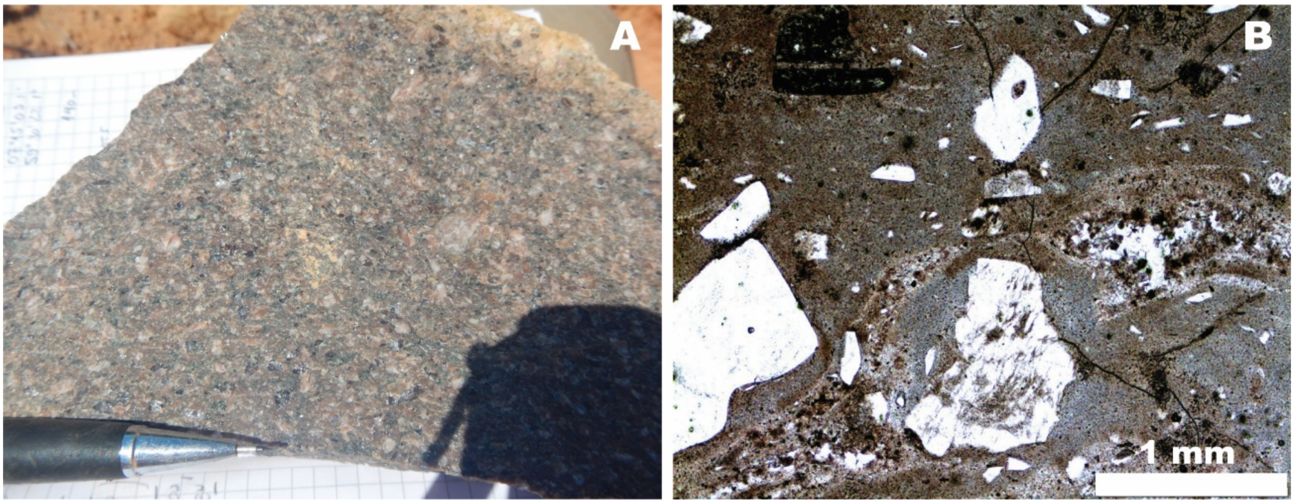

Fig 19 - Macroscopic and microscopic features of sample SS-39. (A) Porphyritic volcanic rock. (B) Quartz and feldspar phenocrysts within a microcrystalline groundmass.

PB-07 - Beneficente Group, Pedro Sara Formation

Location: Outcrop along the course of the Acari river, north of the BR-230 road.

Field features: Extensive outcrop of volcanic rock in the bed of the river.

Description: porphyritic quartz trachyte with 25% of phenocrysts of: (1) subhedral to anhedral broken perthite (0.4 – 3 mm) locally zoned, with inclusions of altered mafic minerals. Locally altered to extensively altered to white mica, hematite, carbonate and quartz; (2) subhedral to anhedral prismatic quartz (~ 0.7 mm) with undulating sector zonation, containing melt inclusions; (3) subhedral globular to prismatic mafic mineral, interpreted as olivine, replaced to serpentine and hematite associated with opaques and apatite; (4) euhedral globular to rhombohedral opaques (0.2 – 0.6 mm). The groundmass is quartz-feldspathic with quench textures.

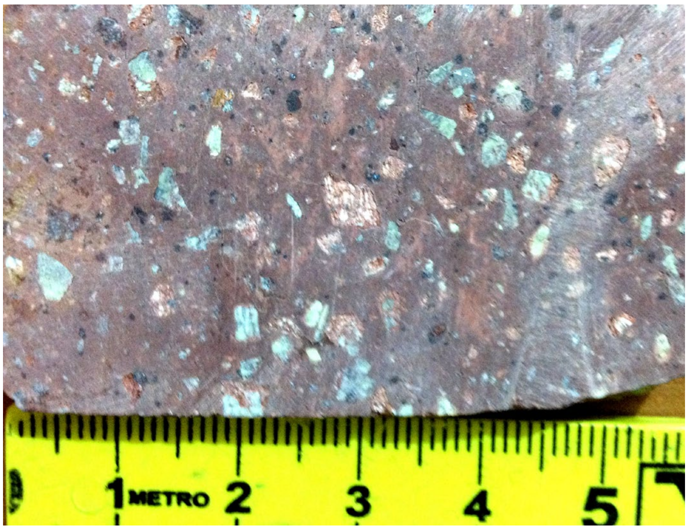

Fig 20 - Macroscopic features of sample PB-07 displaying porphyritic texture with reddish to greenish perthite and plagioclase along with mafic phenocryst immersed in a brownish aphanitic groundmass.

PB-09 - Beneficente Group, Pedro Sara Formation

Location: Outcrop along the course of the Acari river, north of the BR-230 road.

Field features: Extensive outcrop of volcanic rock in the bed of the river.

Description: porphyritic trachyte with 30% of phenocrysts of: (1) subhedral to anhedral broken perthite (0.4 – 4 mm) locally zoned, with inclusions of altered mafic minerals. Locally altered to extensively altered to white mica, hematite, carbonate and quartz; (2) subhedral globular to prismatic mafic mineral (0.3 – 0.7 mm), interpreted as olivine, replaced to serpentine and hematite associated with opaques and apatite; (3) euhedral globular to rhombohedral opaques (0.2 – 0.6 mm). The groundmass is quartz-feldspathic with quench textures.

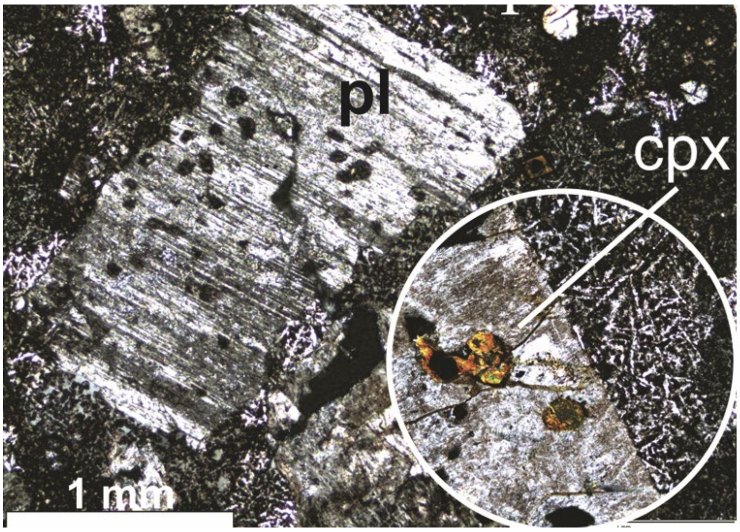

Fig 21 - Microscopic features of sample PB-09 displaying plagioclase phenocrysts with clinopyroxene inclusions immersed in a quench-textured quartz-feldspathic groundmass.

References

1. Cordani, U. G. *et al. Tectonic map of South America*. 2nd ed. (CGMW–CPRM–SEGEMAR, Paris, 2016). Scale 1:5,000,000.

2. Mendes, T.A.A. *et al. Mapa Geológico do Estado de Roraima*. Manaus: SGB CPRM. Scale 1:1.000.000. Projeto Geologia e Recursos Minerais de Roraima. (2022)

3. Vasquez, M.L., Gaia, S.M.S., Chaves, C.L., Silva, C.M.G. (Orgs.) 2024. *Áreas de Relevante Interesse Mineral (ARIM): evolução crustal e metalogenia da Província Mineral do Tapajós*. Serviço Geológico do Brasil (SGB-CPRM), Belém (2024)

4. Rizzotto, G.J., Alves, C.L., Rios, F.S., Gonçalves, G.F., Lopes, L.L. 2019. *Carta de Integração Geológica-Geofísica - Escala 1:300.000*. Goiânia: CPRM – Serviço Geológico do Brasil. (2019)

5. Meloni, R.E., Simões, M.S., Oliveira, A.C.S. 2021. *Áreas de Relevante Interesse Mineral (ARIM): evolução crustal e metalogenia do sudeste do Amazonas – distrito aurífero Juma, estado do Amazonas*. Serviço Geológico do Brasil (SGB-CPRM), Manaus, (2021).
