# Supplementary material for: Large-scale crustal growth driven by LIP magmatism during the Paleoproterozoic
Source: Nat Commun. 2025 Nov 28;16:10779. doi: 10.1038/s41467-025-65826-5 (PMC12663317; doi:10.1038/s41467-025-65826-5)
Supplement: Supplementary file 5 — Supplementary Data 4 [file 41467_2025_65826_MOESM5_ESM.pdf]

Supplementary Data for

Large-scale crustal growth driven by LIP magmatism during the Paleoproterozoic

Matheus S. Simões, Andrew R.C. Kylander-Clark, Marcelo L. Vasquez, Carlos A. Sommer, Lucas M.M. Rossetti, John M. Cottle, Túlio A. Mendes

Zircon CL images

Cycle 1

LA-01

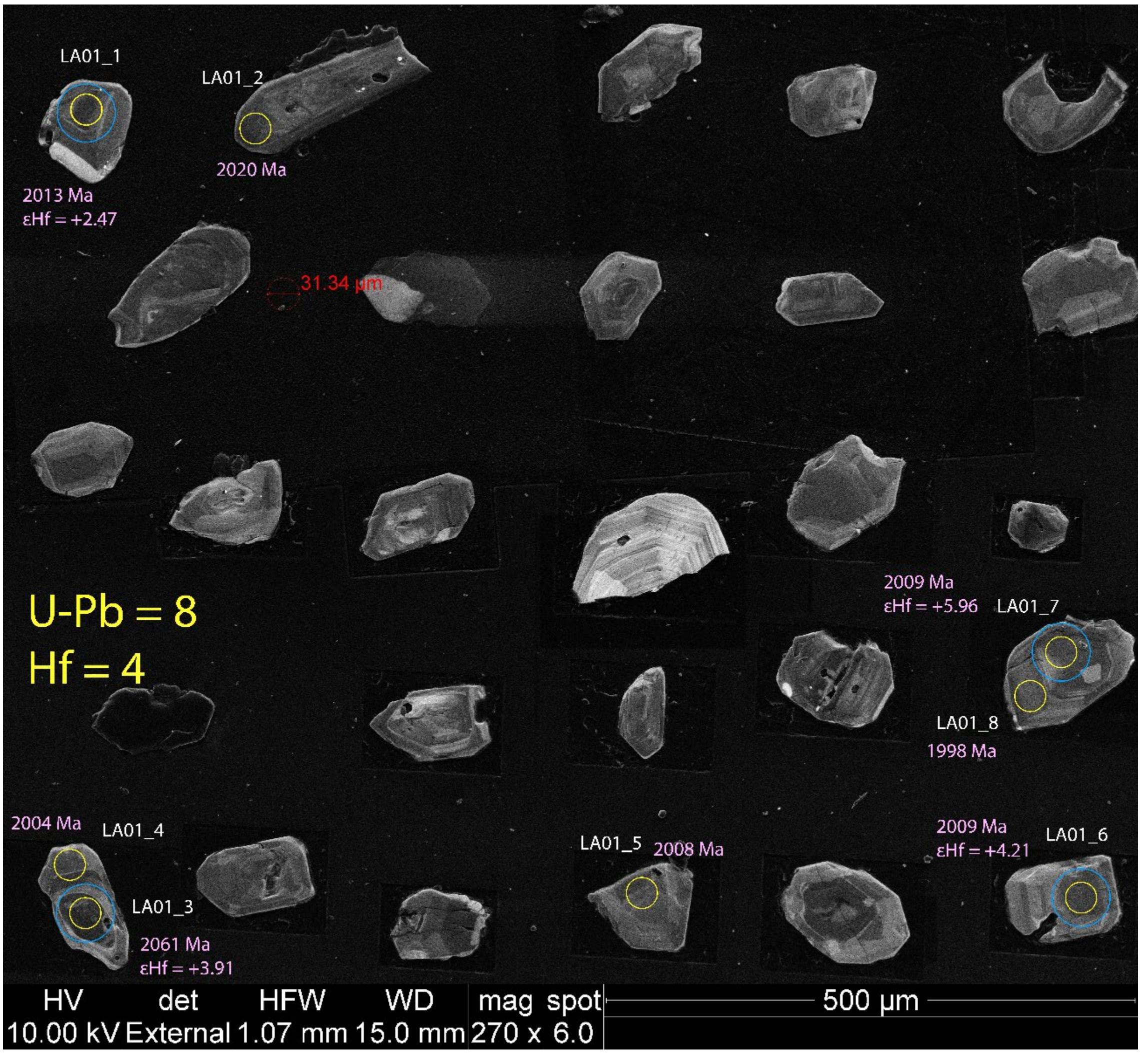
LA01_1
LA01_2
2020 Ma
2013 Ma
εHf = +2.47
31.34 µm
U-Pb = 8
Hf = 4
2009 Ma
εHf = +5.96
LA01_7
LA01_8
1998 Ma
2004 Ma
LA01_4
LA01_5
2008 Ma
2009 Ma
εHf = +4.21
LA01_6
LA01_3
2061 Ma
εHf = +3.91
HV
det
HFW
WD
mag
spot
500 µm
10.00 kV
External
1.07 mm
15.0 mm
270 x
6.0

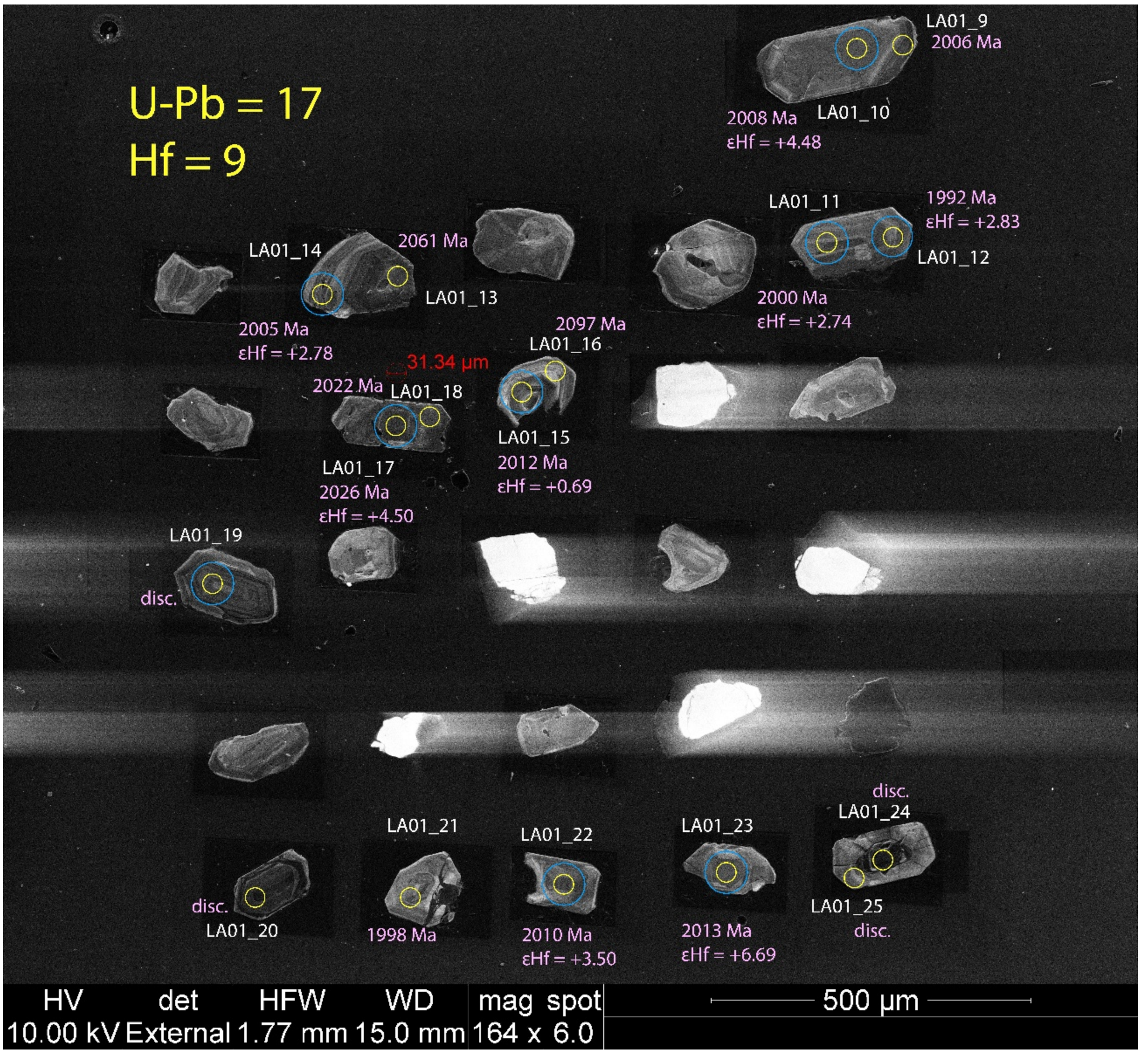

U-Pb = 17
Hf = 9
LA01_9
2006 Ma
2008 Ma
LA01_10
εHf = +4.48
LA01_11
1992 Ma
εHf = +2.83
LA01_12
2000 Ma
εHf = +2.74
LA01_14
2061 Ma
LA01_13
2005 Ma
εHf = +2.78
2097 Ma
LA01_16
31.34 µm
2022 Ma
LA01_18
LA01_15
2012 Ma
εHf = +0.69
LA01_17
2026 Ma
εHf = +4.50
LA01_19
disc.
LA01_21
LA01_22
LA01_23
disc.
LA01_24
disc.
LA01_20
1998 Ma
2010 Ma
εHf = +3.50
2013 Ma
εHf = +6.69
LA01_25
disc.
HV det HFW WD mag spot
10.00 kV External 1.77 mm 15.0 mm 164 x 6.0
500 µm

PB-34

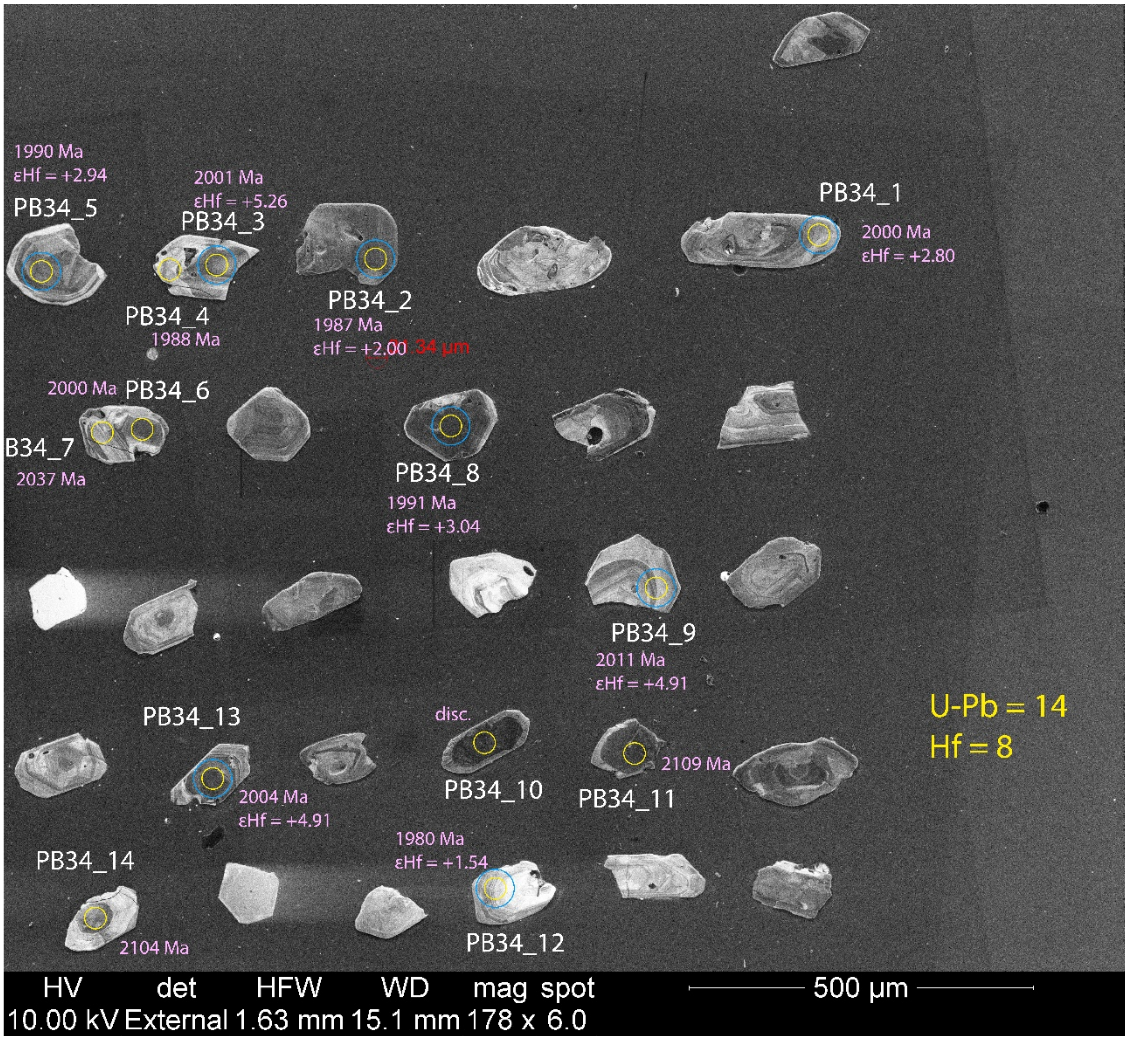

1990 Ma
εHf = +2.94
PB34_5
2001 Ma
εHf = +5.26
PB34_3
PB34_1
2000 Ma
εHf = +2.80
PB34_4
1988 Ma
PB34_2
1987 Ma
εHf = +2.00
2000 Ma
PB34_6
B34_7
2037 Ma
PB34_8
1991 Ma
εHf = +3.04
PB34_9
2011 Ma
εHf = +4.91
U-Pb = 14
Hf = 8
PB34_13
2004 Ma
εHf = +4.91
disc.
PB34_10
2109 Ma
PB34_11
PB34_14
2104 Ma
1980 Ma
εHf = +1.54
PB34_12
HV det HFW WD mag spot
10.00 kV External 1.63 mm 15.1 mm 178 x 6.0
500 µm

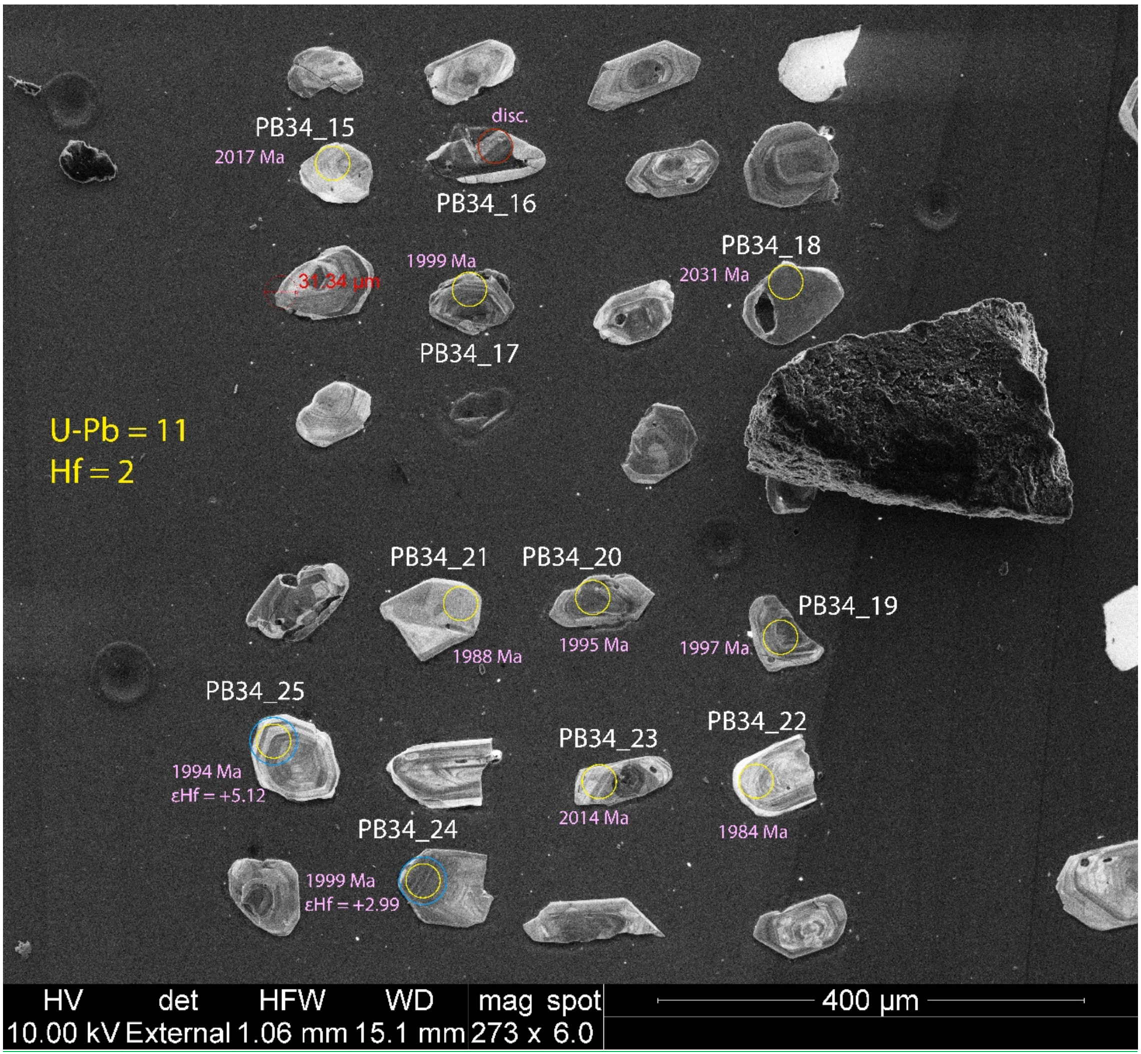
PB34_15
2017 Ma
disc.
PB34_16
1999 Ma
31.34 µm
PB34_18
2031 Ma
PB34_17
U-Pb = 11
Hf = 2
PB34_21
PB34_20
PB34_19
1988 Ma
1995 Ma
1997 Ma
PB34_25
1994 Ma
εHf = +5.12
PB34_23
PB34_22
2014 Ma
1984 Ma
PB34_24
1999 Ma
εHf = +2.99
HV det HFW WD mag spot
10.00 kV External 1.06 mm 15.1 mm 273 x 6.0
400 µm

TM-43

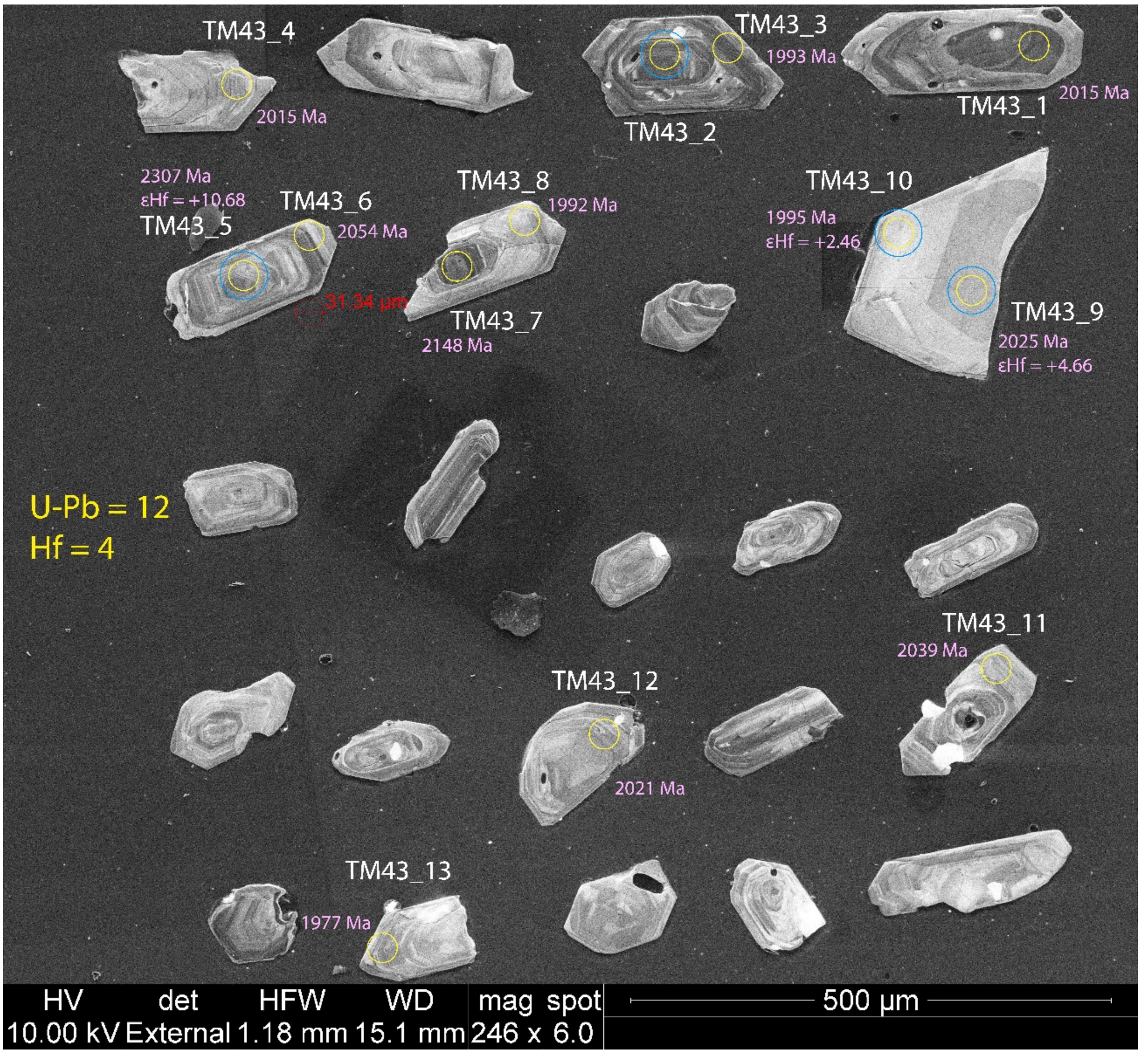

TM43_4
2015 Ma
TM43_3
1993 Ma
TM43_2
TM43_1
2015 Ma
2307 Ma
εHf = +10.68
TM43_5
TM43_6
2054 Ma
TM43_8
1992 Ma
31.34 µm
TM43_7
2148 Ma
TM43_10
1995 Ma
εHf = +2.46
TM43_9
2025 Ma
εHf = +4.66
U-Pb = 12
Hf = 4
TM43_11
2039 Ma
TM43_12
2021 Ma
TM43_13
1977 Ma
HV det HFW WD mag spot
10.00 kV External 1.18 mm 15.1 mm 246 x 6.0
500 µm

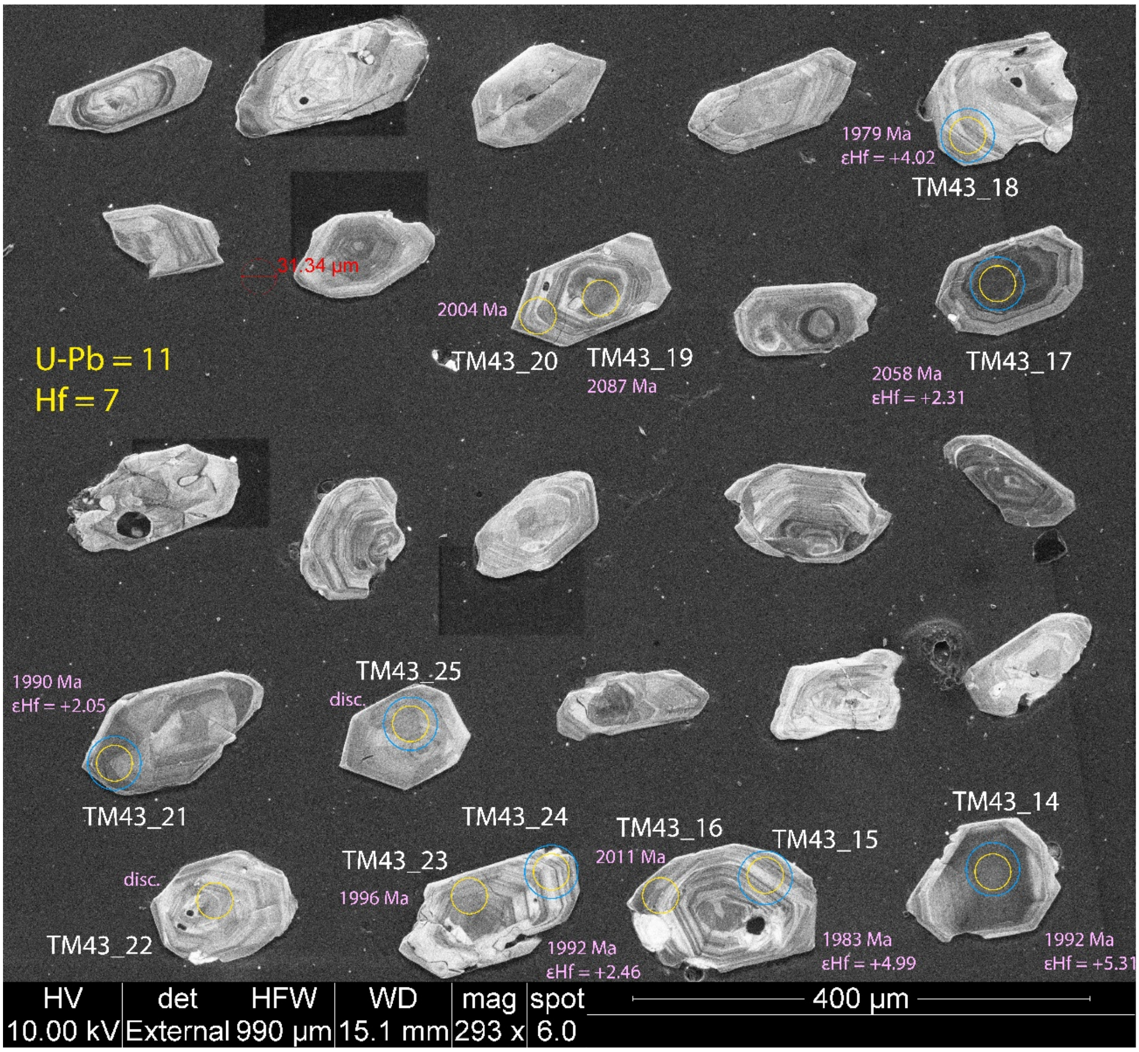
1979 Ma
εHf = +4.02
TM43_18
31.34 µm
2004 Ma
TM43_20
TM43_19
2087 Ma
2058 Ma
εHf = +2.31
TM43_17
U-Pb = 11
Hf = 7
1990 Ma
εHf = +2.05
TM43_25
disc.
TM43_21
TM43_24
TM43_16
2011 Ma
TM43_15
TM43_14
TM43_23
1996 Ma
disc.
TM43_22
1992 Ma
εHf = +2.46
1983 Ma
εHf = +4.99
1992 Ma
εHf = +5.31
HV det HFW WD mag spot
10.00 kV External 990 µm 15.1 mm 293 x 6.0
400 µm

TM-71

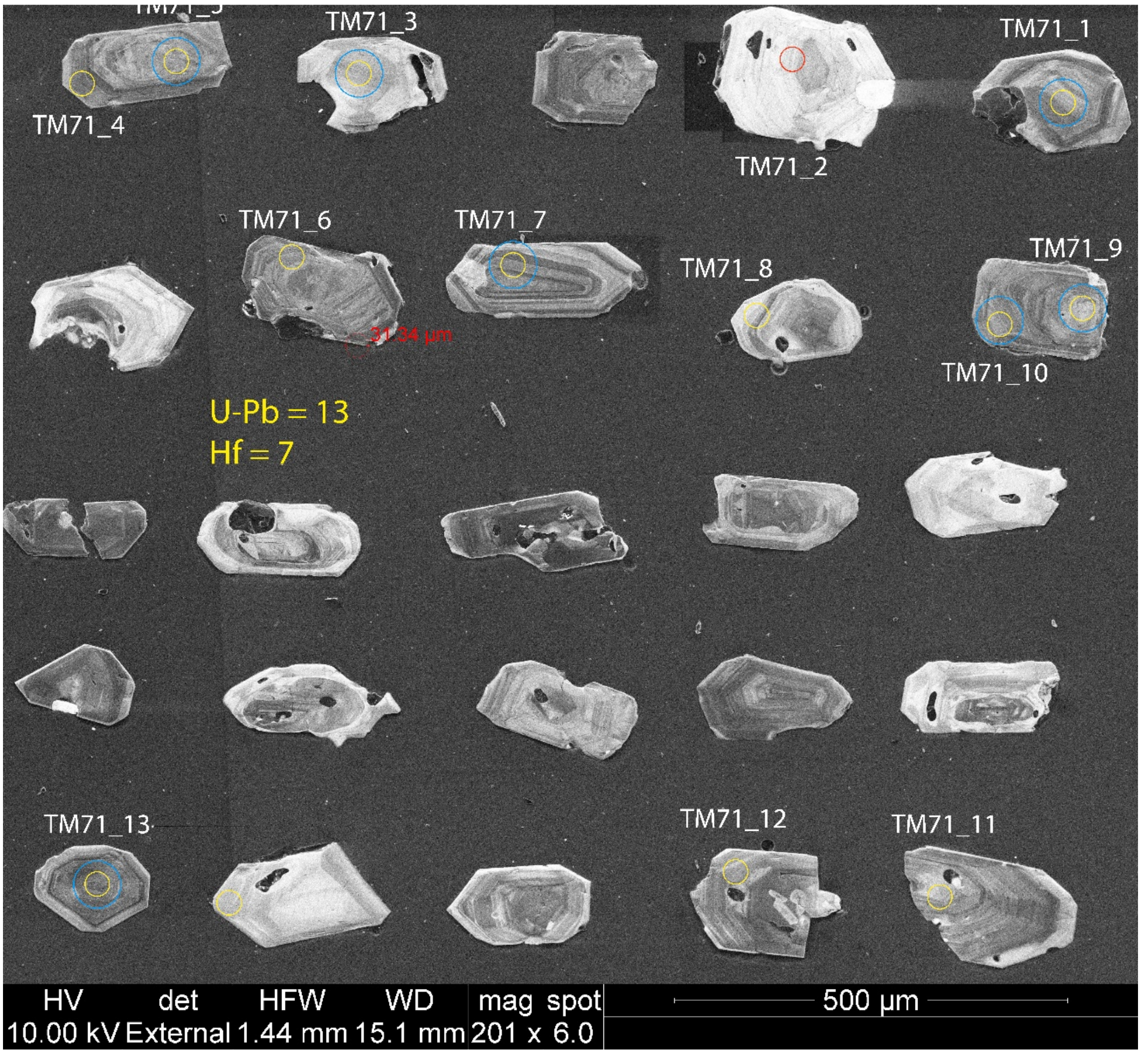
TM71_3
TM71_1
TM71_4
TM71_2
TM71_6
TM71_7
TM71_8
TM71_9
TM71_10
U-Pb = 13
Hf = 7
TM71_13
TM71_12
TM71_11
HV det HFW WD mag spot
10.00 kV External 1.44 mm 15.1 mm 201 x 6.0
500 µm

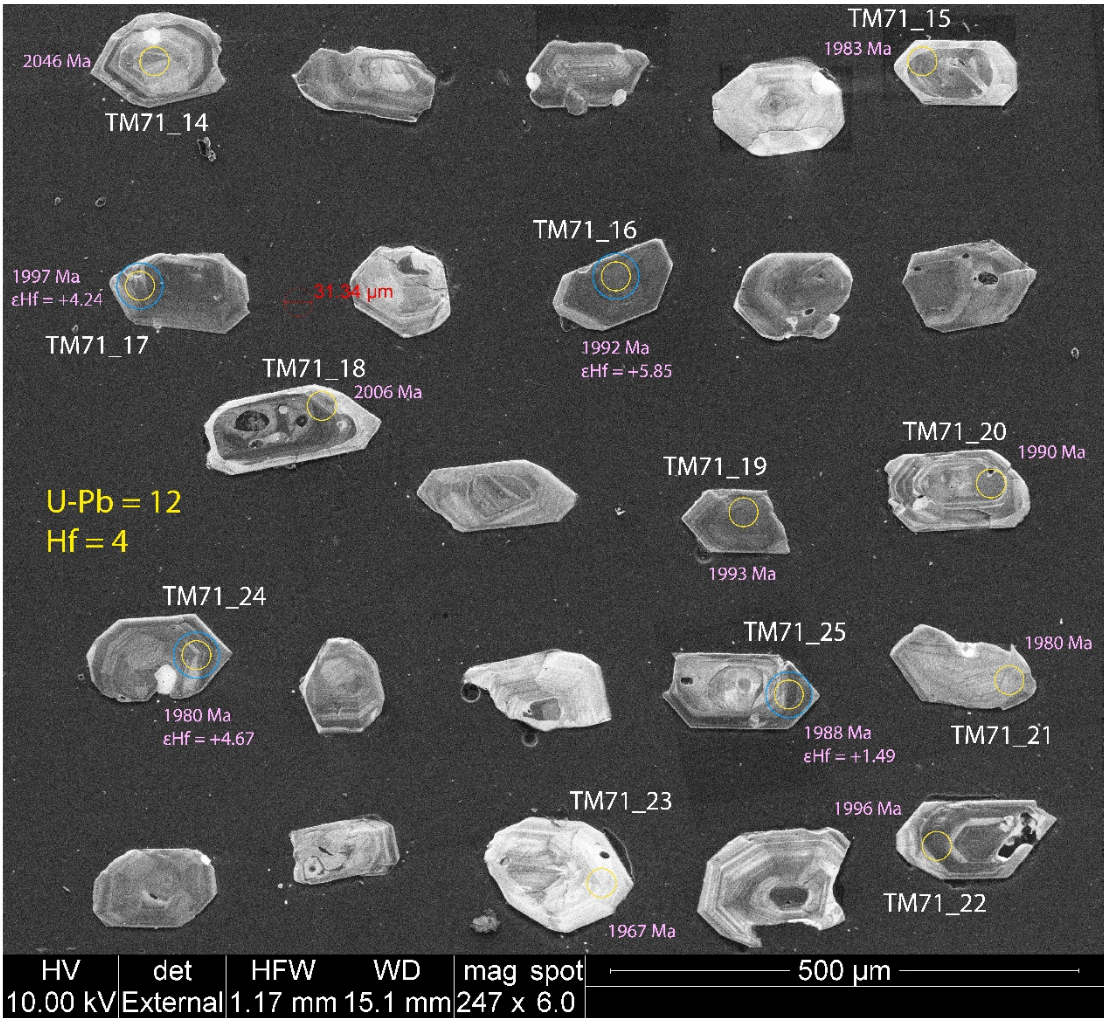
TM71_14
2046 Ma
TM71_15
1983 Ma
TM71_16
1992 Ma
εHf = +5.85
TM71_17
1997 Ma
εHf = +4.24
31.34 µm
TM71_18
2006 Ma
TM71_19
1993 Ma
TM71_20
1990 Ma
U-Pb = 12
Hf = 4
TM71_24
1980 Ma
εHf = +4.67
TM71_25
1988 Ma
εHf = +1.49
TM71_21
1980 Ma
TM71_23
1967 Ma
TM71_22
1996 Ma
HV 10.00 kV
det External
HFW 1.17 mm
WD 15.1 mm
mag 247 x
spot 6.0
500 µm

VR-05

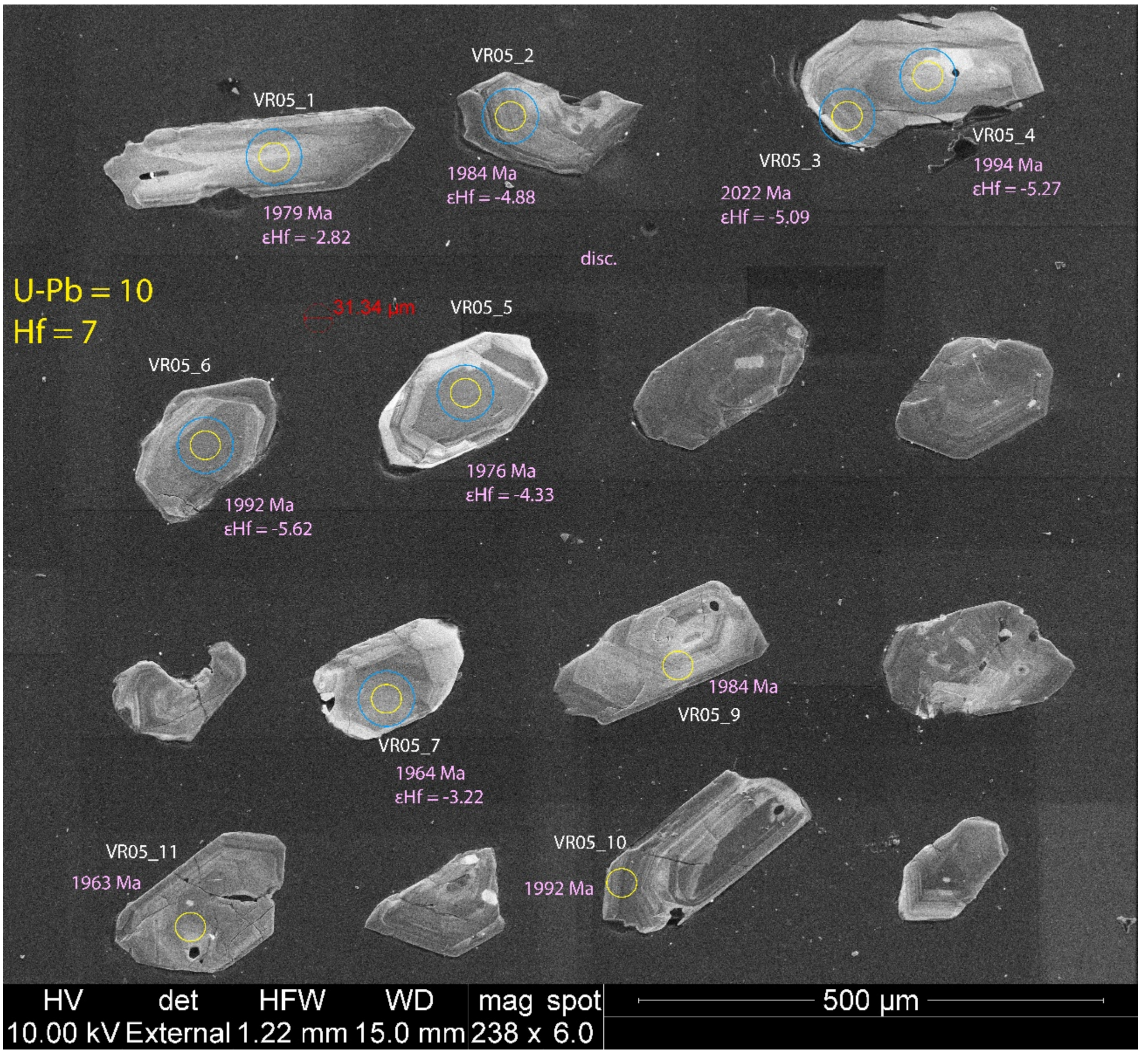

VR05_1
1979 Ma
εHf = -2.82
VR05_2
1984 Ma
εHf = -4.88
VR05_3
2022 Ma
εHf = -5.09
VR05_4
1994 Ma
εHf = -5.27
disc.
U-Pb = 10
Hf = 7
31.34 µm
VR05_5
1976 Ma
εHf = -4.33
VR05_6
1992 Ma
εHf = -5.62
1984 Ma
VR05_9
VR05_7
1964 Ma
εHf = -3.22
VR05_11
1963 Ma
VR05_10
1992 Ma
HV det HFW WD mag spot
10.00 kV External 1.22 mm 15.0 mm 238 x 6.0
500 µm

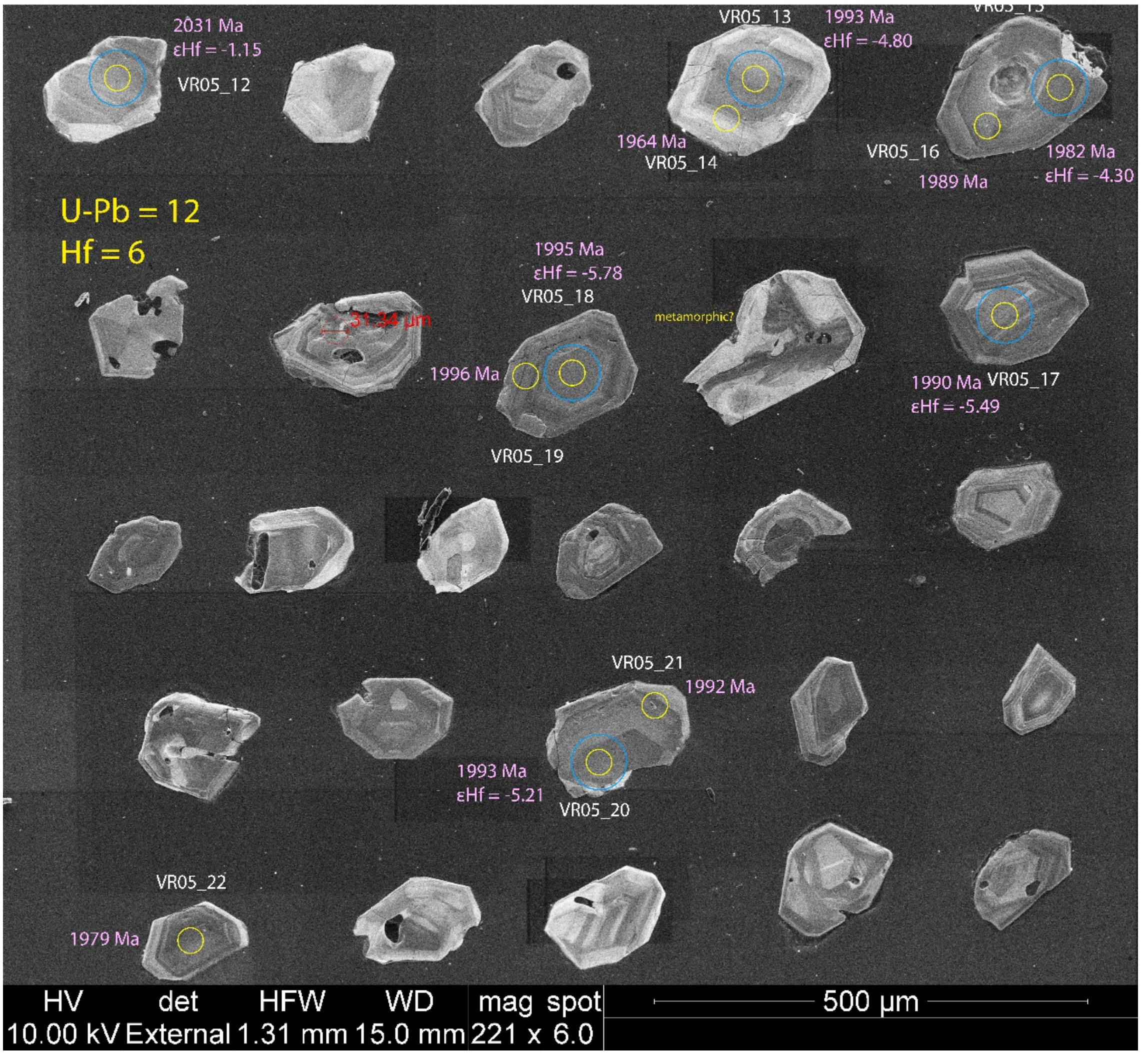
2031 Ma
εHf = -1.15
VR05_12
VR05_13
1993 Ma
εHf = -4.80
1964 Ma
VR05_14
VR05_16
1989 Ma
1982 Ma
εHf = -4.30
U-Pb = 12
Hf = 6
1995 Ma
εHf = -5.78
VR05_18
metamorphic?
1996 Ma
VR05_19
1990 Ma
VR05_17
εHf = -5.49
VR05_21
1992 Ma
1993 Ma
εHf = -5.21
VR05_20
VR05_22
1979 Ma
HV det HFW WD mag spot
10.00 kV External 1.31 mm 15.0 mm 221 x 6.0
500 µm

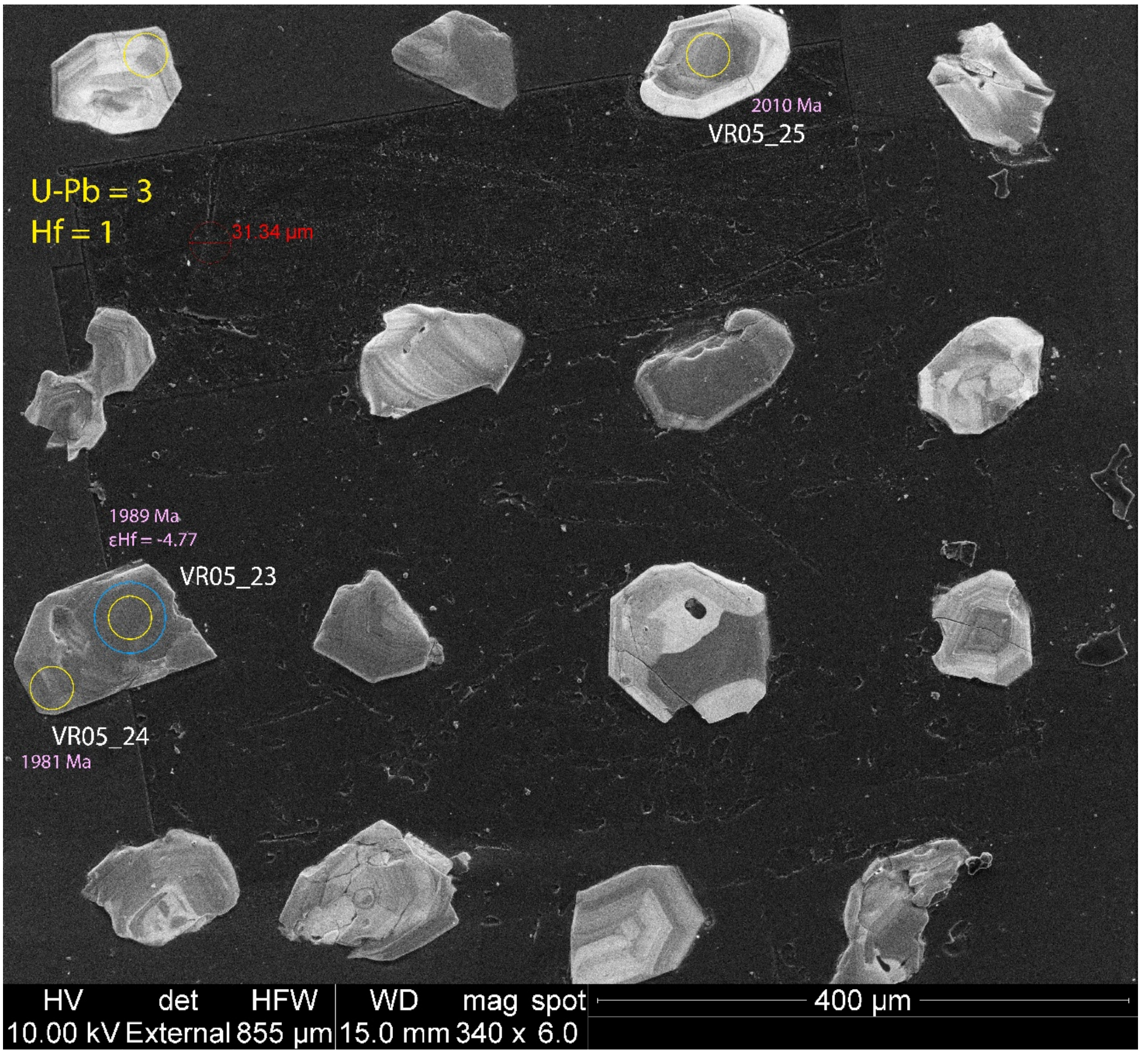
2010 Ma
VR05_25
U-Pb = 3
Hf = 1
31.34 µm
1989 Ma
εHf = -4.77
VR05_23
VR05_24
1981 Ma
HV det HFW WD mag spot
10.00 kV External 855 µm 15.0 mm 340 x 6.0
400 µm

VR-04

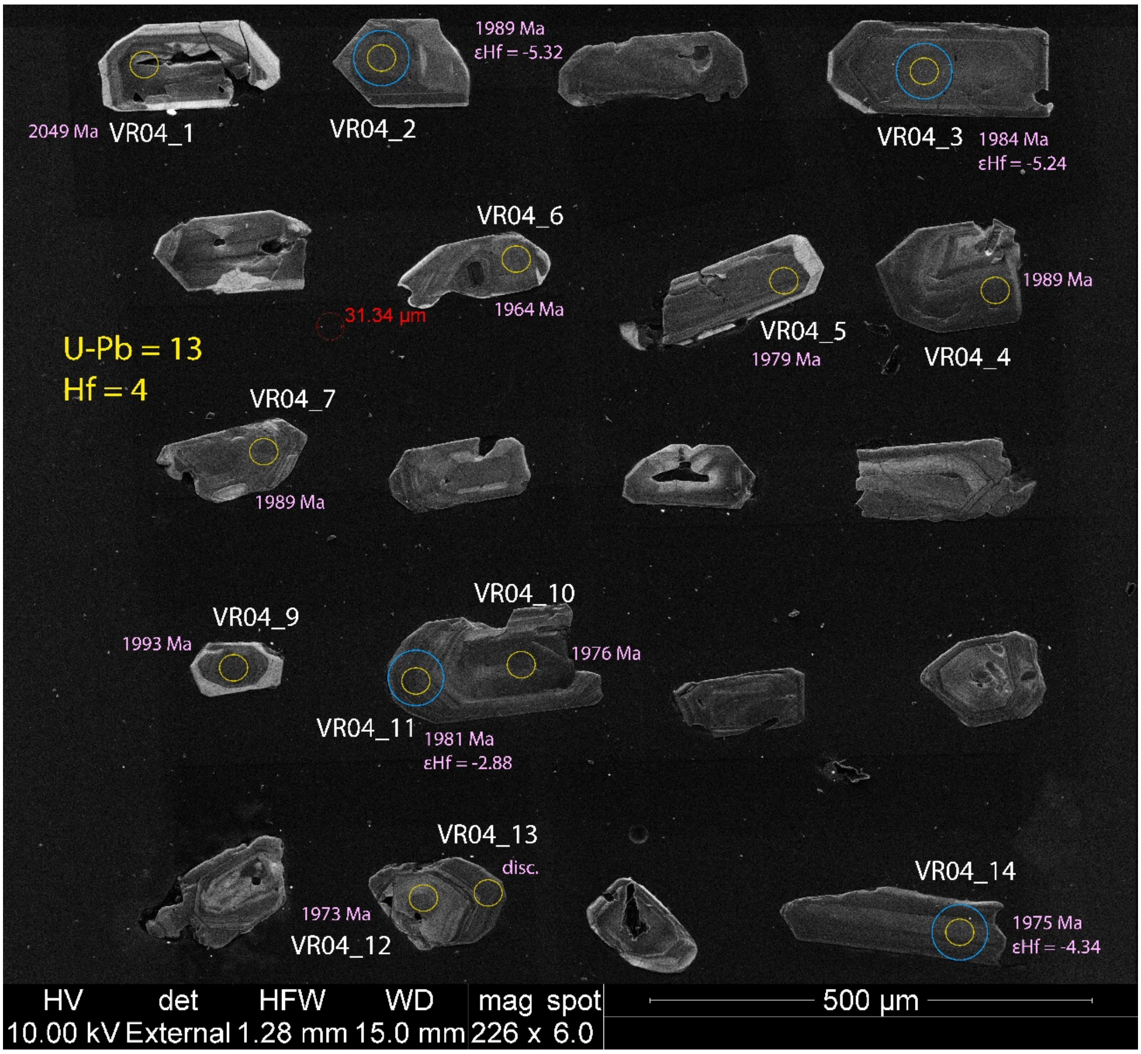

VR04_1
2049 Ma
VR04_2
1989 Ma
εHf = -5.32
VR04_3
1984 Ma
εHf = -5.24
VR04_6
1964 Ma
31.34 µm
VR04_5
1979 Ma
VR04_4
1989 Ma
U-Pb = 13
Hf = 4
VR04_7
1989 Ma
VR04_9
1993 Ma
VR04_10
1976 Ma
VR04_11
1981 Ma
εHf = -2.88
VR04_13
disc.
VR04_12
1973 Ma
VR04_14
1975 Ma
εHf = -4.34
HV
det
HFW
WD
mag
spot
10.00 kV
External
1.28 mm
15.0 mm
226 x
6.0
500 µm

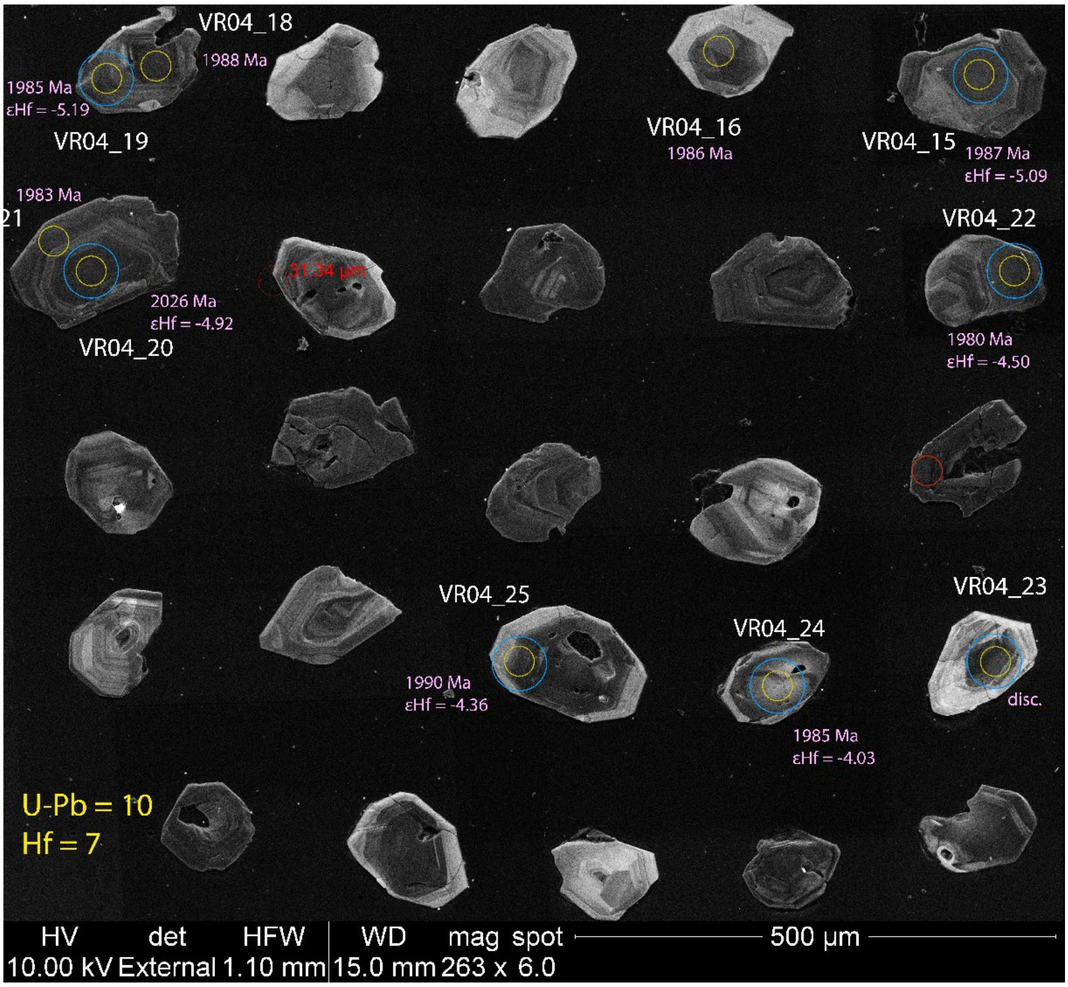
VR04_18
1988 Ma
1985 Ma
εHf = -5.19
VR04_19
VR04_16
1986 Ma
VR04_15
1987 Ma
εHf = -5.09
1983 Ma
21
2026 Ma
εHf = -4.92
VR04_20
31.34 µm
VR04_22
1980 Ma
εHf = -4.50
VR04_25
1990 Ma
εHf = -4.36
VR04_24
1985 Ma
εHf = -4.03
VR04_23
disc.
U-Pb = 10
Hf = 7
HV det HFW WD mag spot 500 µm
10.00 kV External 1.10 mm 15.0 mm 263 x 6.0

Cycle 2

MA-01

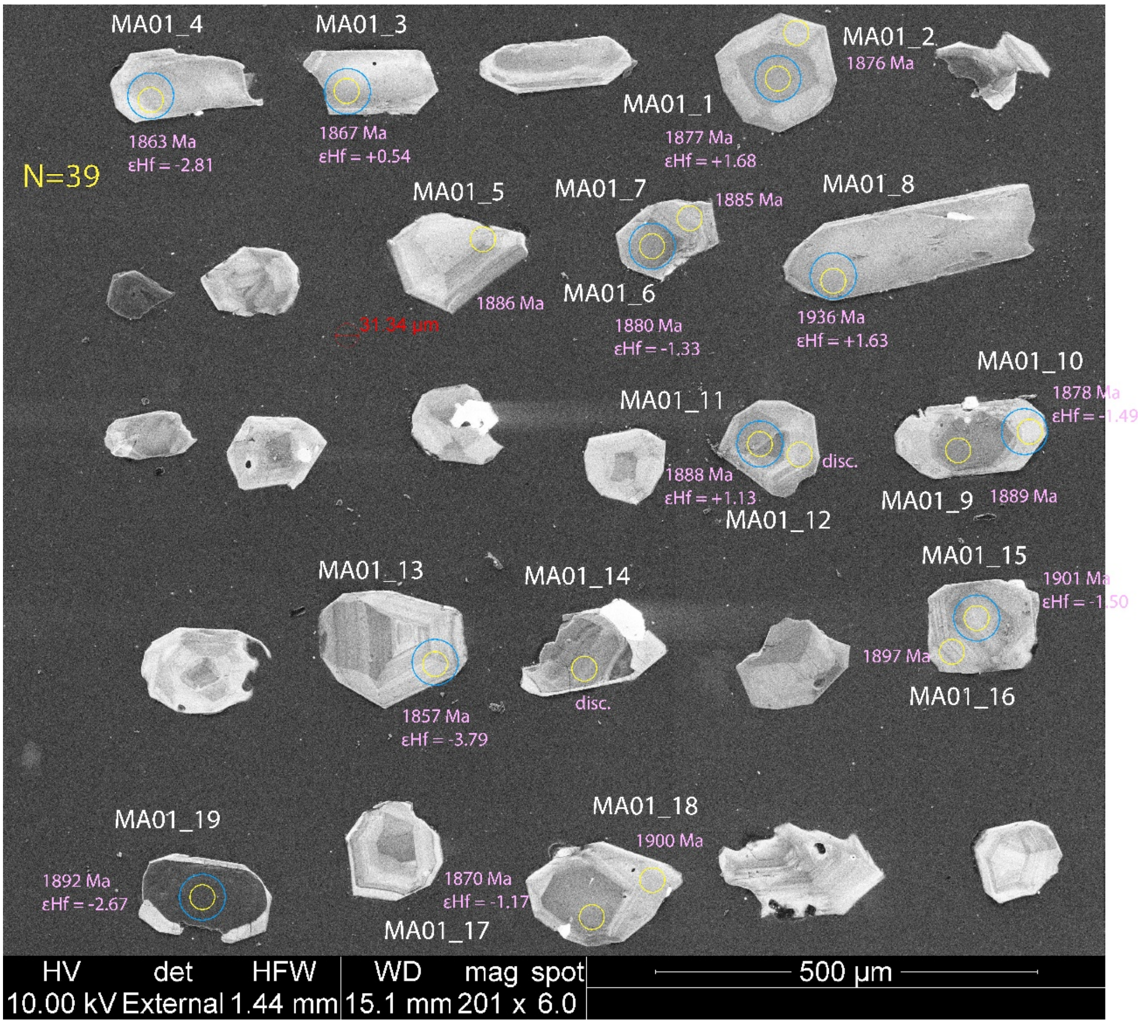

MA01_4
MA01_3
MA01_2
1876 Ma
MA01_1
1863 Ma
εHf = -2.81
1867 Ma
εHf = +0.54
1877 Ma
εHf = +1.68
N=39
MA01_5
MA01_7
1885 Ma
MA01_8
MA01_6
1886 Ma
1880 Ma
εHf = -1.33
1936 Ma
εHf = +1.63
MA01_10
1878 Ma
εHf = -1.49
MA01_11
disc.
1888 Ma
εHf = +1.13
MA01_9
1889 Ma
MA01_12
MA01_15
MA01_13
MA01_14
1901 Ma
εHf = -1.50
1897 Ma
MA01_16
disc.
1857 Ma
εHf = -3.79
MA01_18
MA01_19
1900 Ma
1892 Ma
εHf = -2.67
1870 Ma
εHf = -1.17
MA01_17
HV det HFW WD mag spot
10.00 kV External 1.44 mm 15.1 mm 201 x 6.0
500 µm

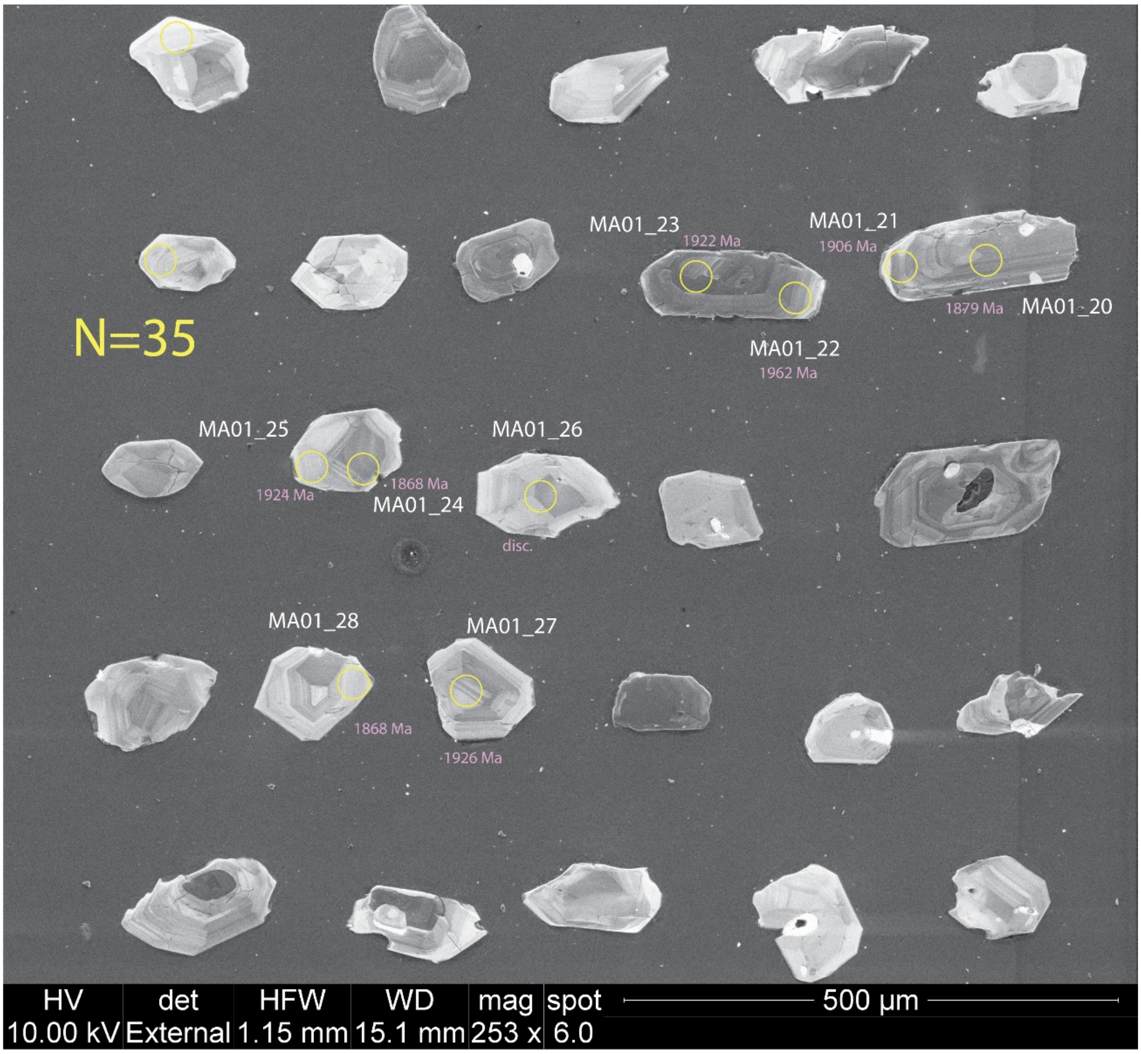

N=35
MA01_23
1922 Ma
MA01_21
1906 Ma
1879 Ma
MA01_20
MA01_22
1962 Ma
MA01_25
1924 Ma
1868 Ma
MA01_24
MA01_26
disc.
MA01_28
1868 Ma
MA01_27
1926 Ma
HV 10.00 kV
det External
HFW 1.15 mm
WD 15.1 mm
mag 253 x
spot 6.0
500 µm

MA-02

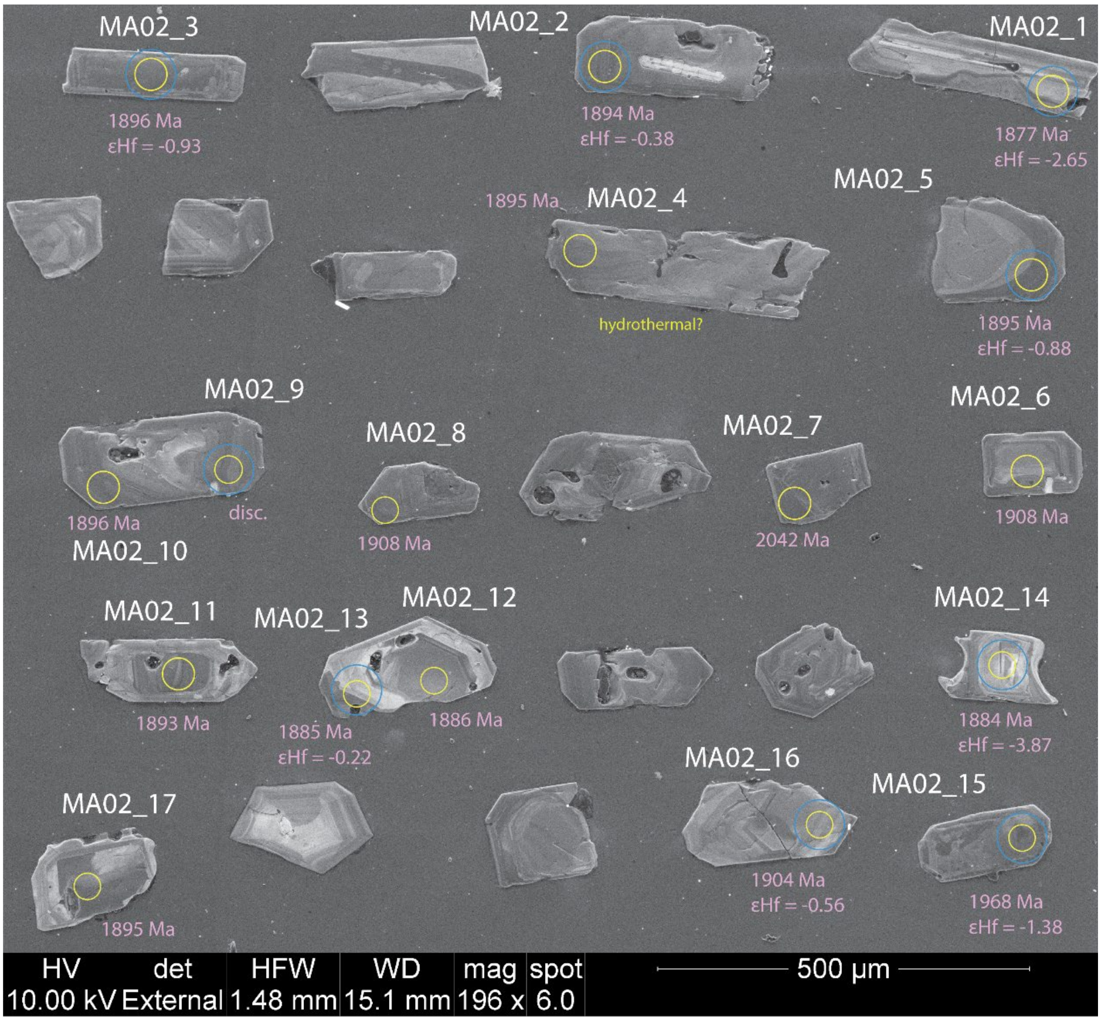

MA02_3
1896 Ma
εHf = -0.93
MA02_2
1894 Ma
εHf = -0.38
MA02_1
1877 Ma
εHf = -2.65
1895 Ma
MA02_4
hydrothermal?
MA02_5
1895 Ma
εHf = -0.88
MA02_9
1896 Ma
disc.
MA02_10
MA02_8
1908 Ma
MA02_7
2042 Ma
MA02_6
1908 Ma
MA02_11
1893 Ma
MA02_13
1885 Ma
εHf = -0.22
MA02_12
1886 Ma
MA02_14
1884 Ma
εHf = -3.87
MA02_16
1904 Ma
εHf = -0.56
MA02_15
1968 Ma
εHf = -1.38
MA02_17
1895 Ma
HV det HFW WD mag spot
10.00 kV External 1.48 mm 15.1 mm 196 x 6.0
500 μm

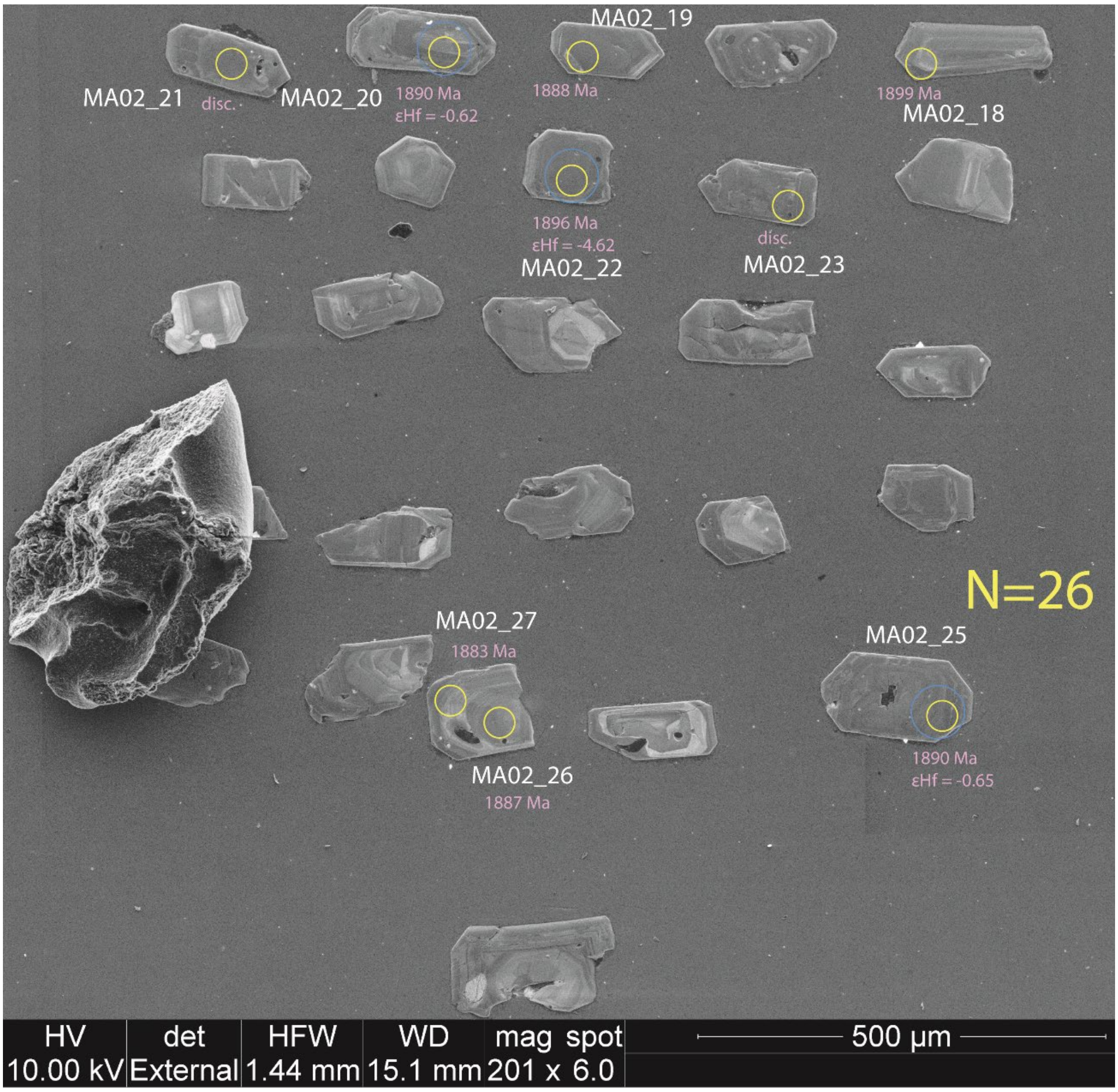
MA02_19
MA02_21
disc.
MA02_20
1890 Ma
εHf = -0.62
1888 Ma
1899 Ma
MA02_18
1896 Ma
εHf = -4.62
MA02_22
disc.
MA02_23
N=26
MA02_27
1883 Ma
MA02_25
MA02_26
1887 Ma
1890 Ma
εHf = -0.65
HV
det
HFW
WD
mag
spot
500 μm
10.00 kV
External
1.44 mm
15.1 mm
201 x
6.0

TM-11

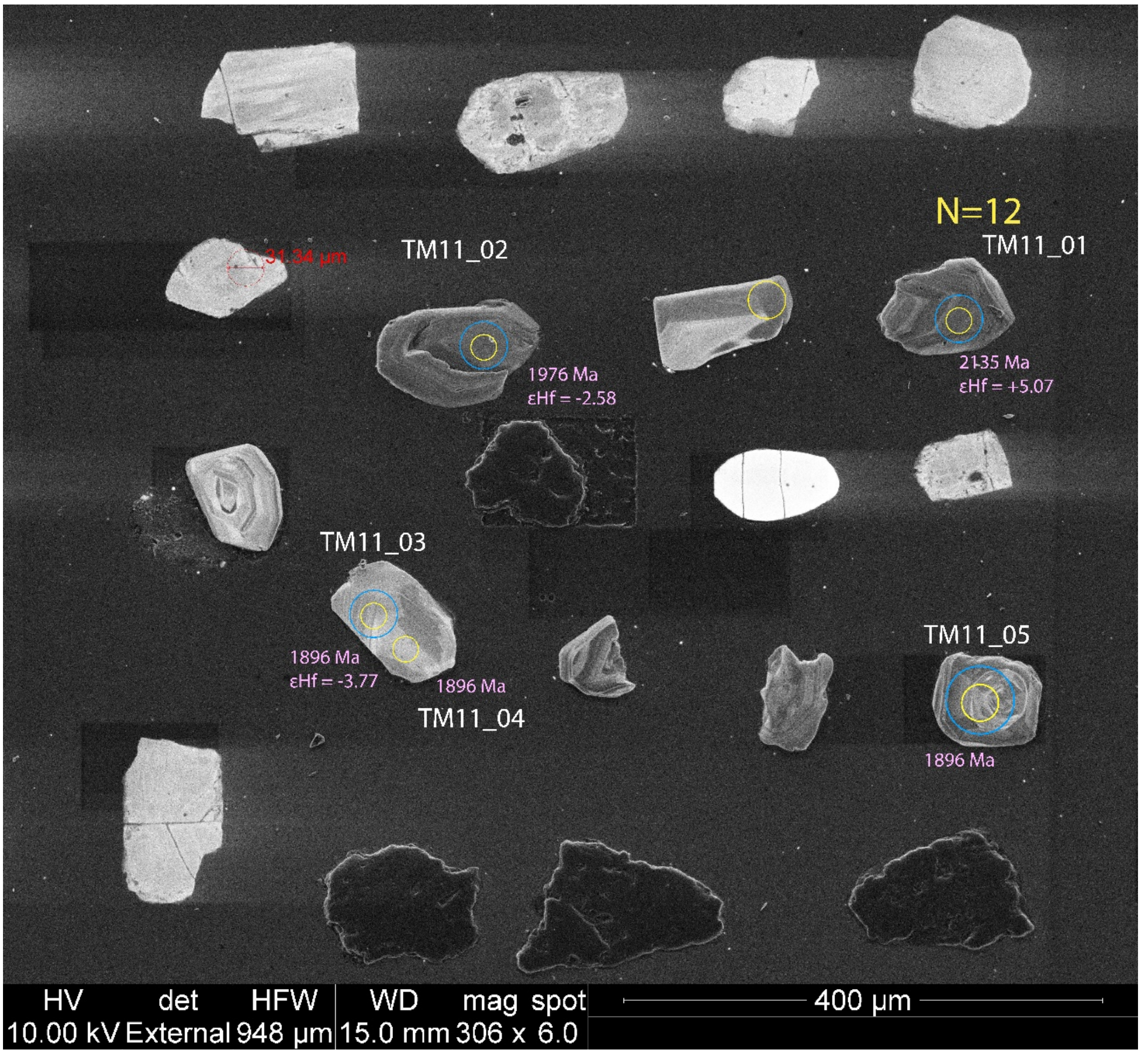

N=12
TM11_01
2135 Ma
εHf = +5.07
TM11_02
1976 Ma
εHf = -2.58
TM11_03
1896 Ma
εHf = -3.77
1896 Ma
TM11_04
TM11_05
1896 Ma
HV det HFW WD mag spot
10.00 kV External 948 µm 15.0 mm 306 x 6.0
400 µm

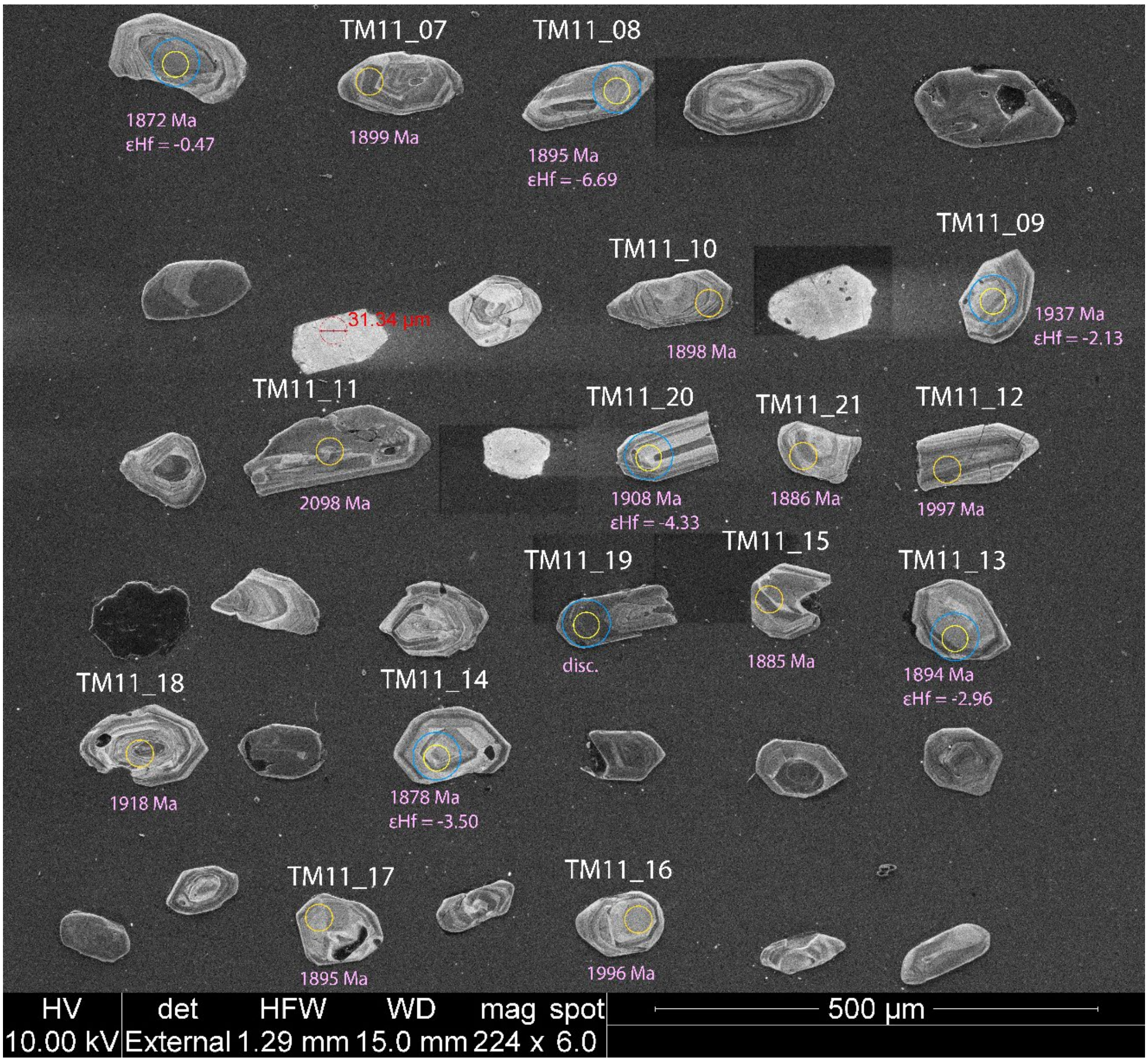

TM11_07
TM11_08
1872 Ma
εHf = -0.47
1899 Ma
1895 Ma
εHf = -6.69
TM11_10
TM11_09
31.34 µm
1898 Ma
1937 Ma
εHf = -2.13
TM11_11
TM11_20
TM11_21
TM11_12
2098 Ma
1908 Ma
εHf = -4.33
1886 Ma
1997 Ma
TM11_19
TM11_15
TM11_13
disc.
1885 Ma
1894 Ma
εHf = -2.96
TM11_18
TM11_14
1918 Ma
1878 Ma
εHf = -3.50
TM11_17
TM11_16
1895 Ma
1996 Ma
HV
det
HFW
WD
mag
spot
500 µm
10.00 kV
External
1.29 mm
15.0 mm
224 x
6.0

Cycle 3

GLR-24

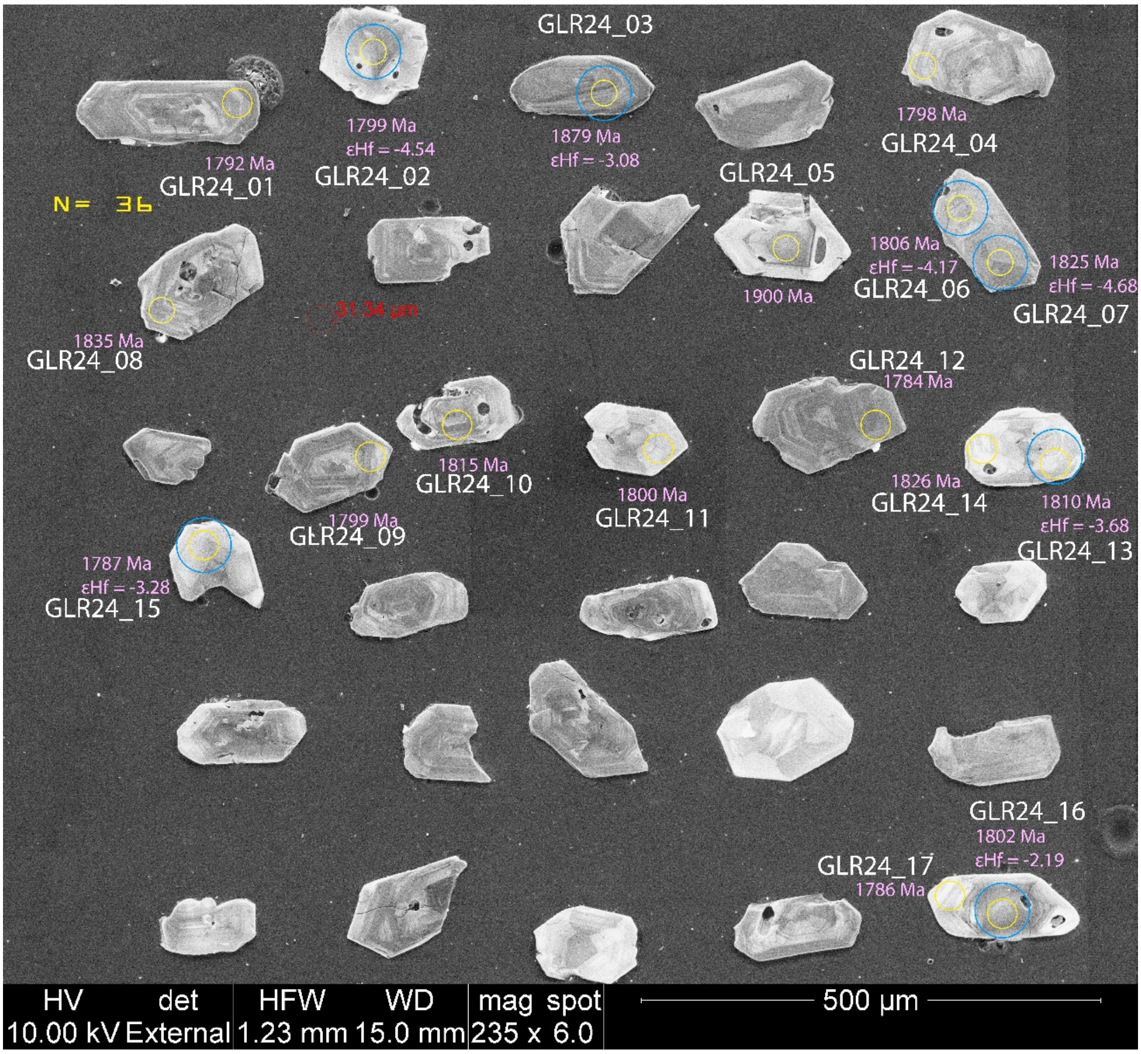
GLR24_03
1792 Ma
GLR24_01
N= 36
1799 Ma
εHf = -4.54
GLR24_02
1879 Ma
εHf = -3.08
GLR24_05
1798 Ma
GLR24_04
1806 Ma
εHf = -4.17
GLR24_06
1825 Ma
εHf = -4.68
GLR24_07
1900 Ma
1835 Ma
GLR24_08
GLR24_12
1784 Ma
1815 Ma
GLR24_10
1800 Ma
GLR24_11
1826 Ma
GLR24_14
1810 Ma
εHf = -3.68
GLR24_13
1799 Ma
GLR24_09
1787 Ma
εHf = -3.28
GLR24_15
GLR24_16
1802 Ma
εHf = -2.19
GLR24_17
1786 Ma
HV det HFW WD mag spot
10.00 kV External 1.23 mm 15.0 mm 235 x 6.0
500 µm

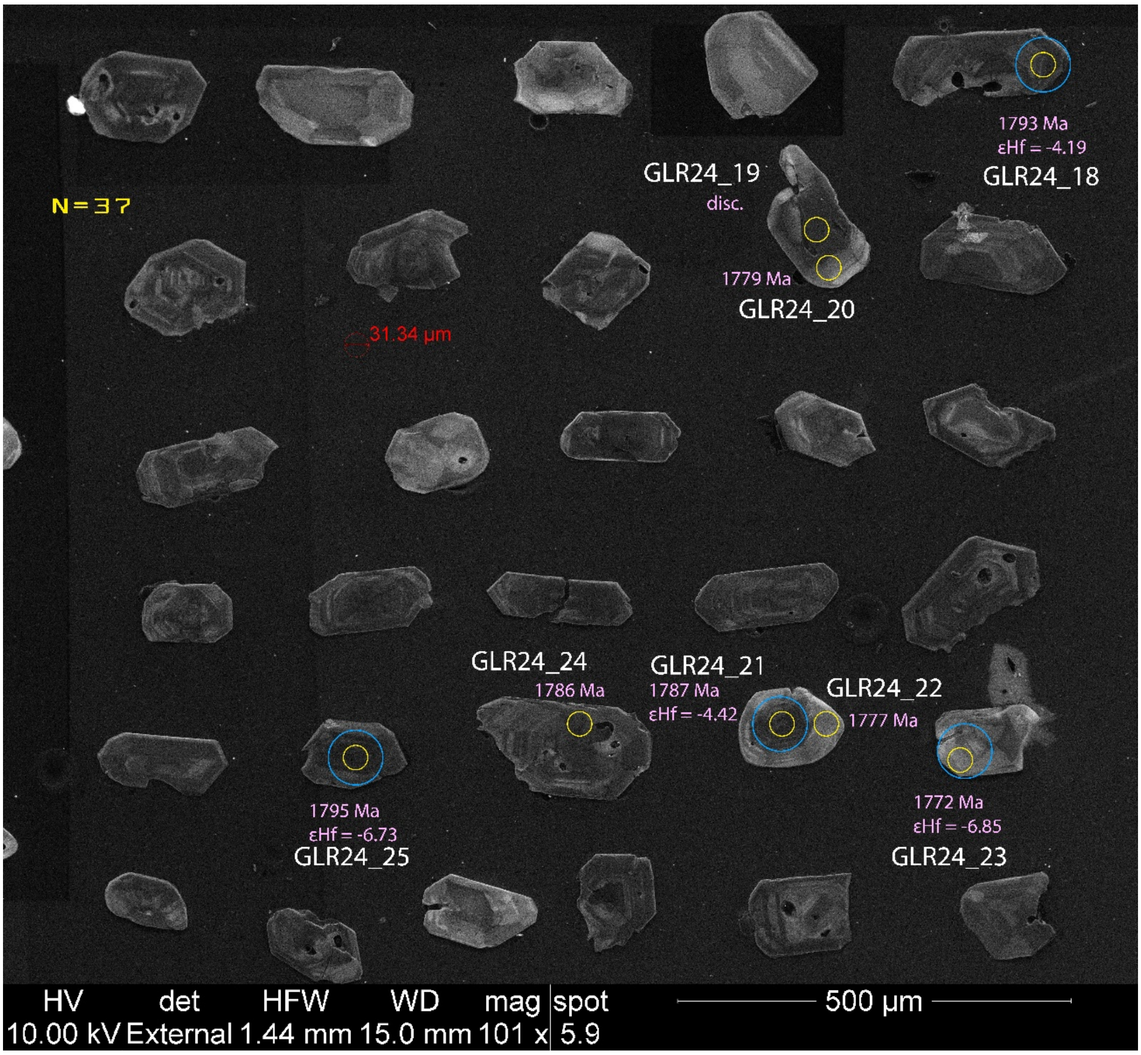
N=37
GLR24_19
disc.
1793 Ma
εHf = -4.19
GLR24_18
1779 Ma
GLR24_20
31.34 µm
GLR24_24
1786 Ma
GLR24_21
1787 Ma
εHf = -4.42
GLR24_22
1777 Ma
1795 Ma
εHf = -6.73
GLR24_25
1772 Ma
εHf = -6.85
GLR24_23
HV det HFW WD mag spot
10.00 kV External 1.44 mm 15.0 mm 101 x 5.9
500 µm

GLR-35

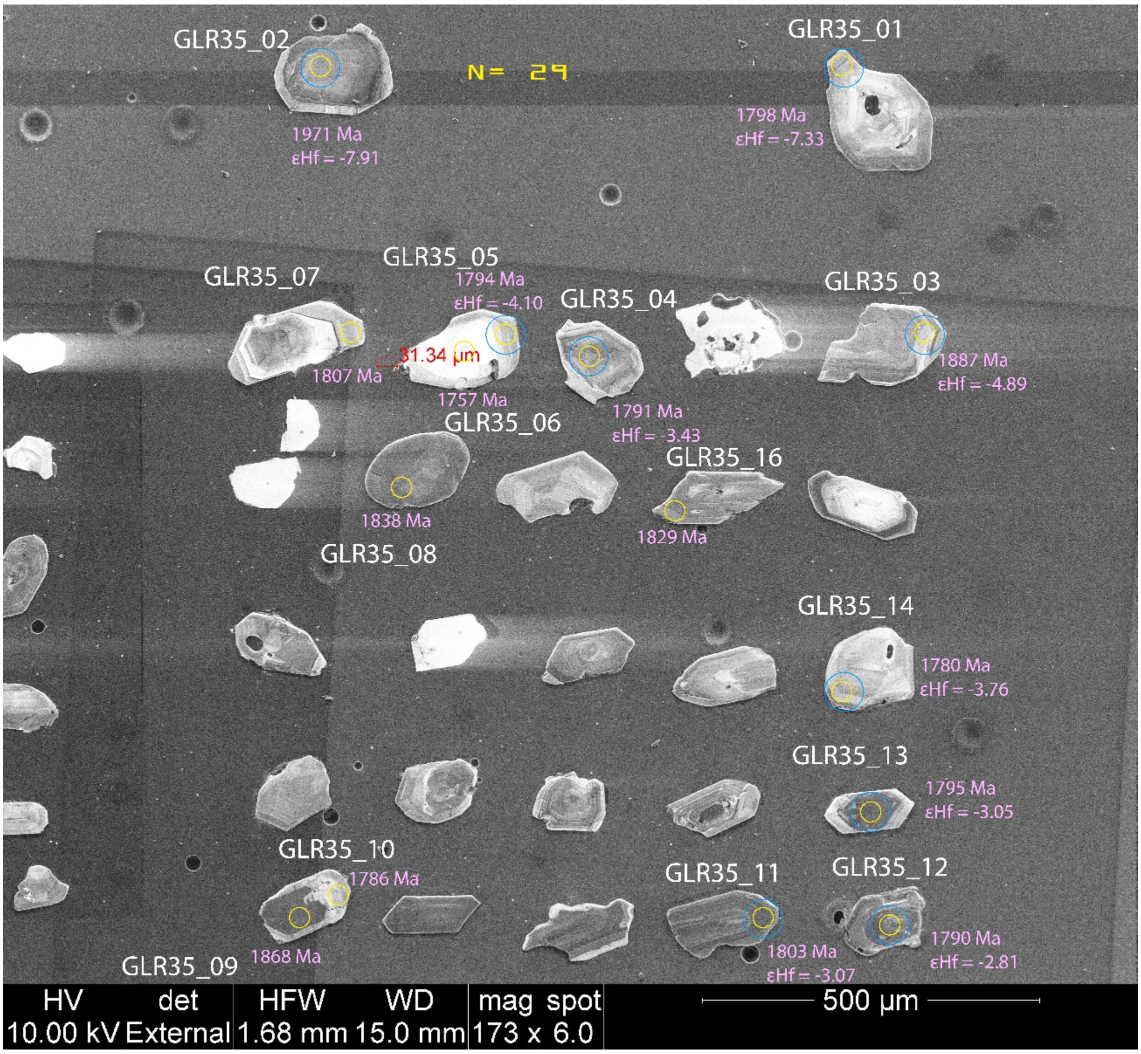

GLR35_02
N= 29
GLR35_01
1971 Ma
εHf = -7.91
1798 Ma
εHf = -7.33
GLR35_07
GLR35_05
1794 Ma
εHf = -4.10
GLR35_04
GLR35_03
1807 Ma
31.34 μm
1757 Ma
1887 Ma
εHf = -4.89
1791 Ma
εHf = -3.43
GLR35_06
GLR35_16
1838 Ma
1829 Ma
GLR35_08
GLR35_14
1780 Ma
εHf = -3.76
GLR35_13
1795 Ma
εHf = -3.05
GLR35_10
1786 Ma
GLR35_11
GLR35_12
1803 Ma
εHf = -3.07
1790 Ma
εHf = -2.81
GLR35_09
1868 Ma
HV det HFW WD mag spot
10.00 kV External 1.68 mm 15.0 mm 173 x 6.0
500 μm

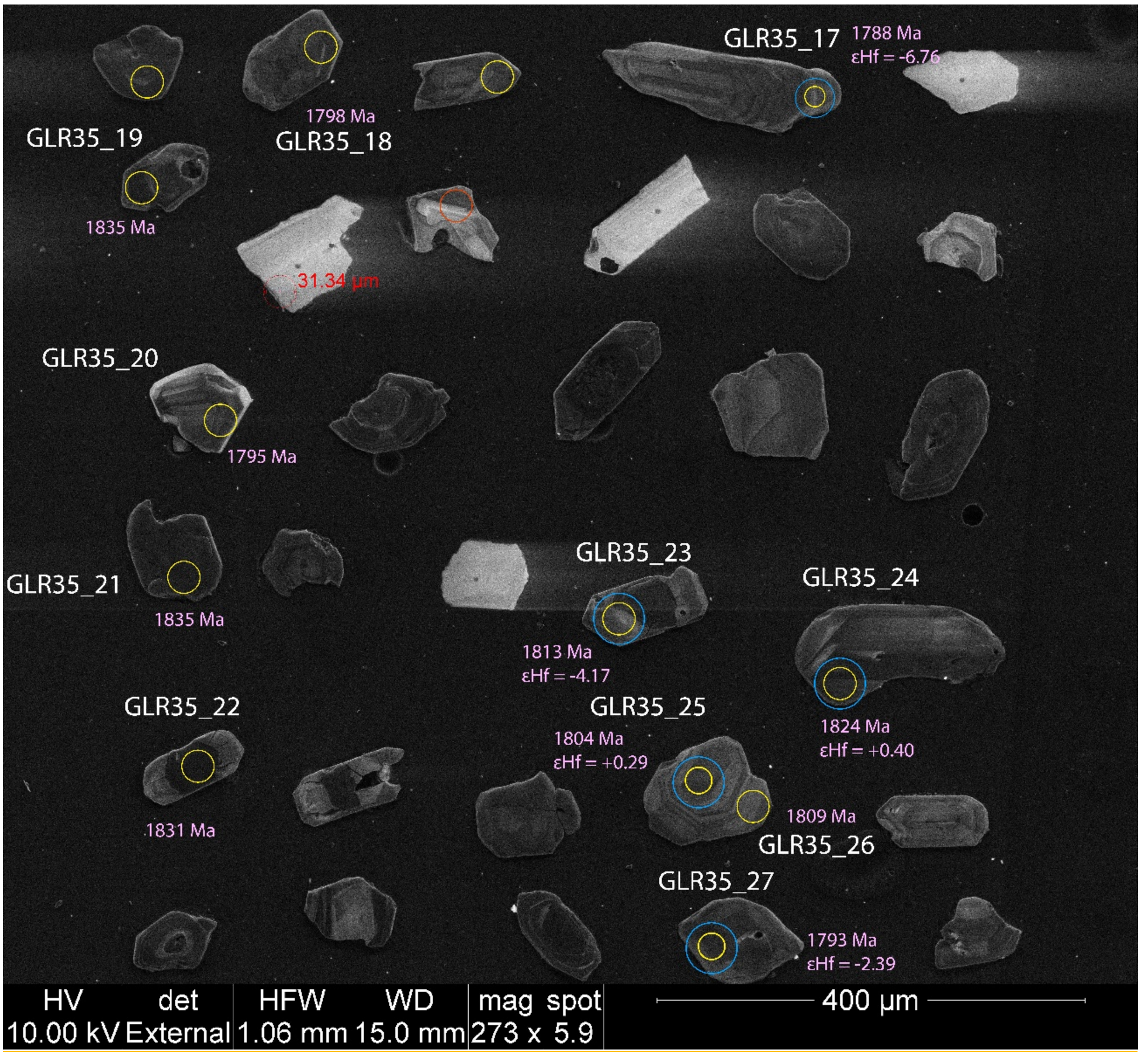
GLR35_17
1788 Ma
εHf = -6.76
1798 Ma
GLR35_18
GLR35_19
1835 Ma
31.34 μm
GLR35_20
1795 Ma
GLR35_21
1835 Ma
GLR35_23
1813 Ma
εHf = -4.17
GLR35_24
1824 Ma
εHf = +0.40
GLR35_22
1831 Ma
GLR35_25
1804 Ma
εHf = +0.29
1809 Ma
GLR35_26
GLR35_27
1793 Ma
εHf = -2.39
HV det HFW WD mag spot
10.00 kV External 1.06 mm 15.0 mm 273 x 5.9
400 μm

PGR-27

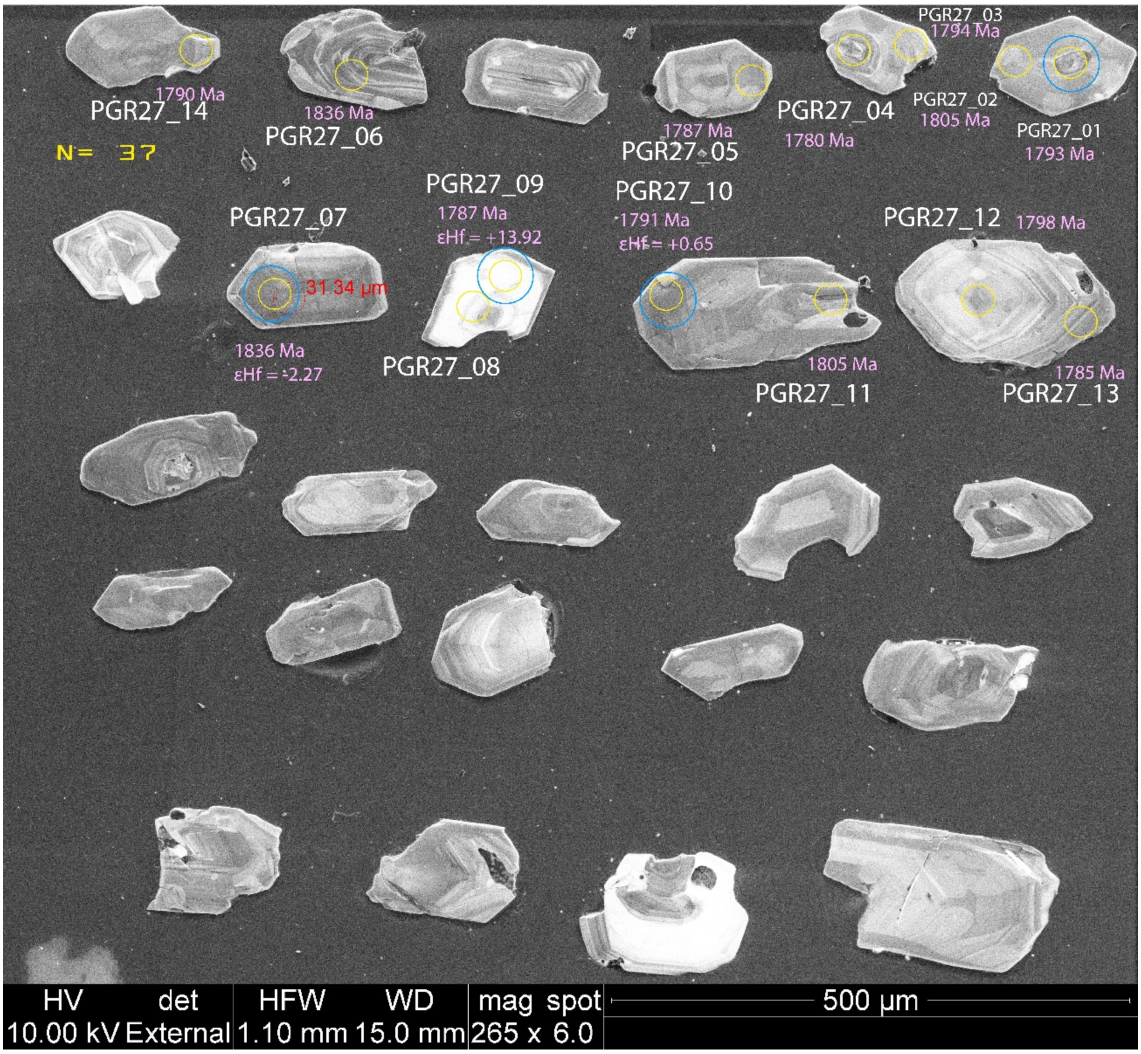

PGR27_14
1790 Ma
PGR27_06
1836 Ma
N= 37
1787 Ma
PGR27_05
PGR27_04
1780 Ma
PGR27_03
1794 Ma
PGR27_02
1805 Ma
PGR27_01
1793 Ma
PGR27_07
31.34 µm
1836 Ma
εHf = -2.27
PGR27_09
1787 Ma
εHf = +13.92
PGR27_08
PGR27_10
1791 Ma
εHf = +0.65
1805 Ma
PGR27_11
PGR27_12
1798 Ma
1785 Ma
PGR27_13
HV
det
HFW
WD
mag
spot
10.00 kV
External
1.10 mm
15.0 mm
265 x
6.0
500 µm

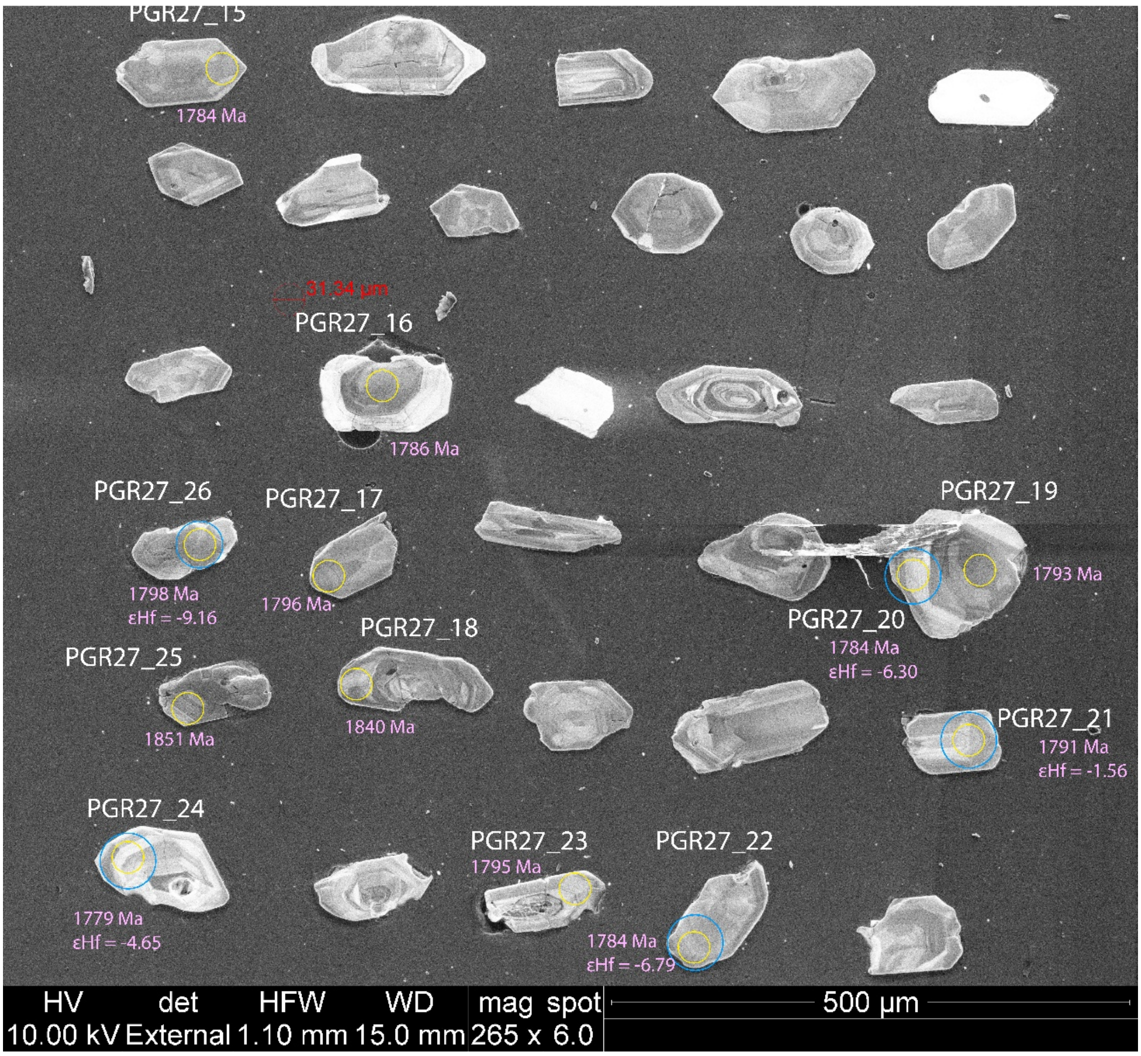
PGR27_15
1784 Ma
31.34 µm
PGR27_16
1786 Ma
PGR27_26
1798 Ma
εHf = -9.16
PGR27_17
1796 Ma
PGR27_19
1793 Ma
PGR27_20
1784 Ma
εHf = -6.30
PGR27_18
1840 Ma
PGR27_25
1851 Ma
PGR27_21
1791 Ma
εHf = -1.56
PGR27_24
1779 Ma
εHf = -4.65
PGR27_23
1795 Ma
PGR27_22
1784 Ma
εHf = -6.79
HV det HFW WD mag spot
10.00 kV External 1.10 mm 15.0 mm 265 x 6.0
500 µm

SS-39A

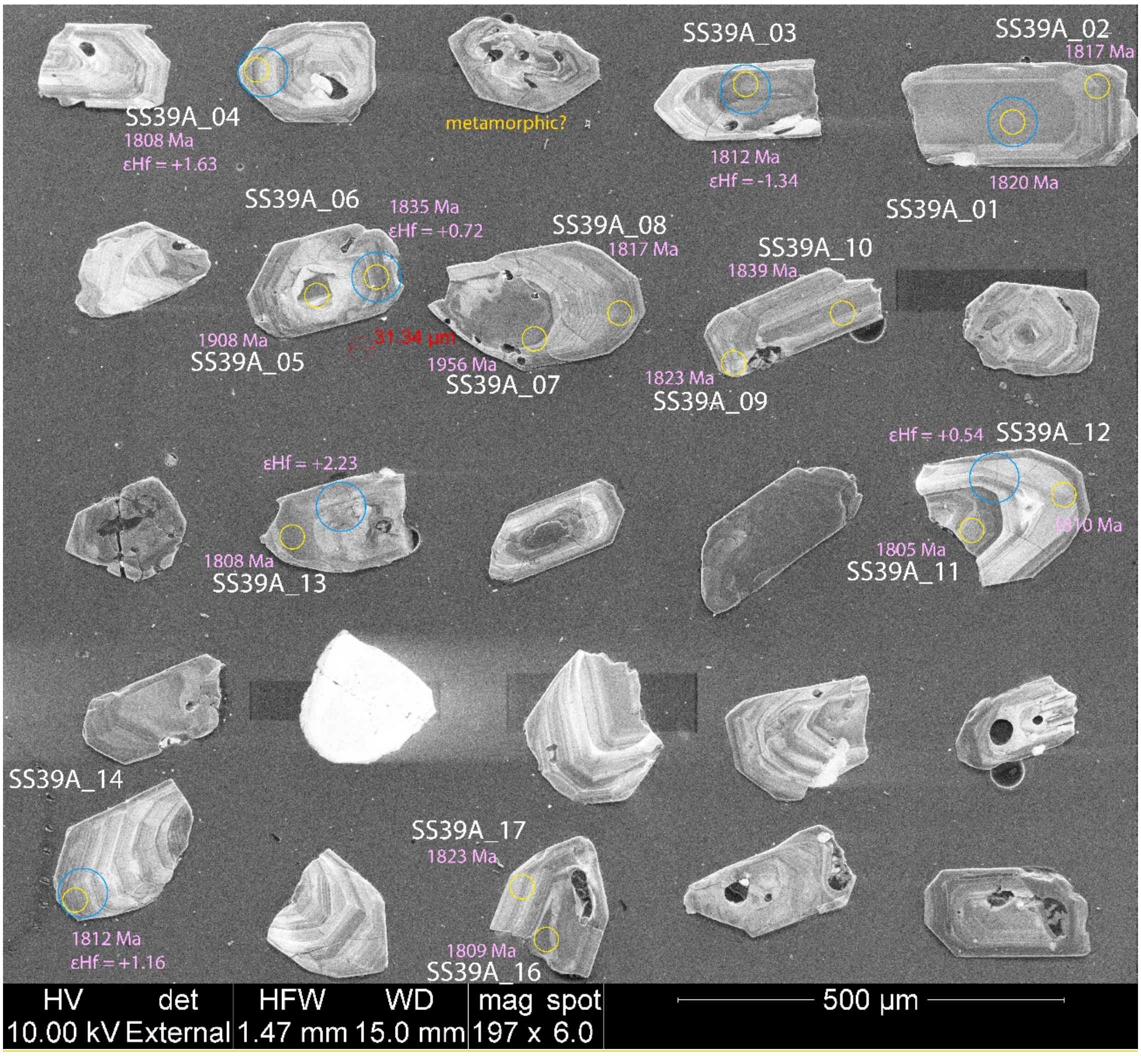
SS39A_04
1808 Ma
εHf = +1.63
metamorphic?
SS39A_03
1812 Ma
εHf = -1.34
SS39A_02
1817 Ma
1820 Ma
SS39A_01
SS39A_06
1835 Ma
εHf = +0.72
SS39A_08
1817 Ma
SS39A_10
1839 Ma
1908 Ma
SS39A_05
31.34 µm
1956 Ma
SS39A_07
1823 Ma
SS39A_09
εHf = +0.54
SS39A_12
εHf = +2.23
1810 Ma
1808 Ma
SS39A_13
1805 Ma
SS39A_11
SS39A_14
SS39A_17
1823 Ma
1812 Ma
εHf = +1.16
1809 Ma
SS39A_16
HV det HFW WD mag spot
10.00 kV External 1.47 mm 15.0 mm 197 x 6.0
500 µm

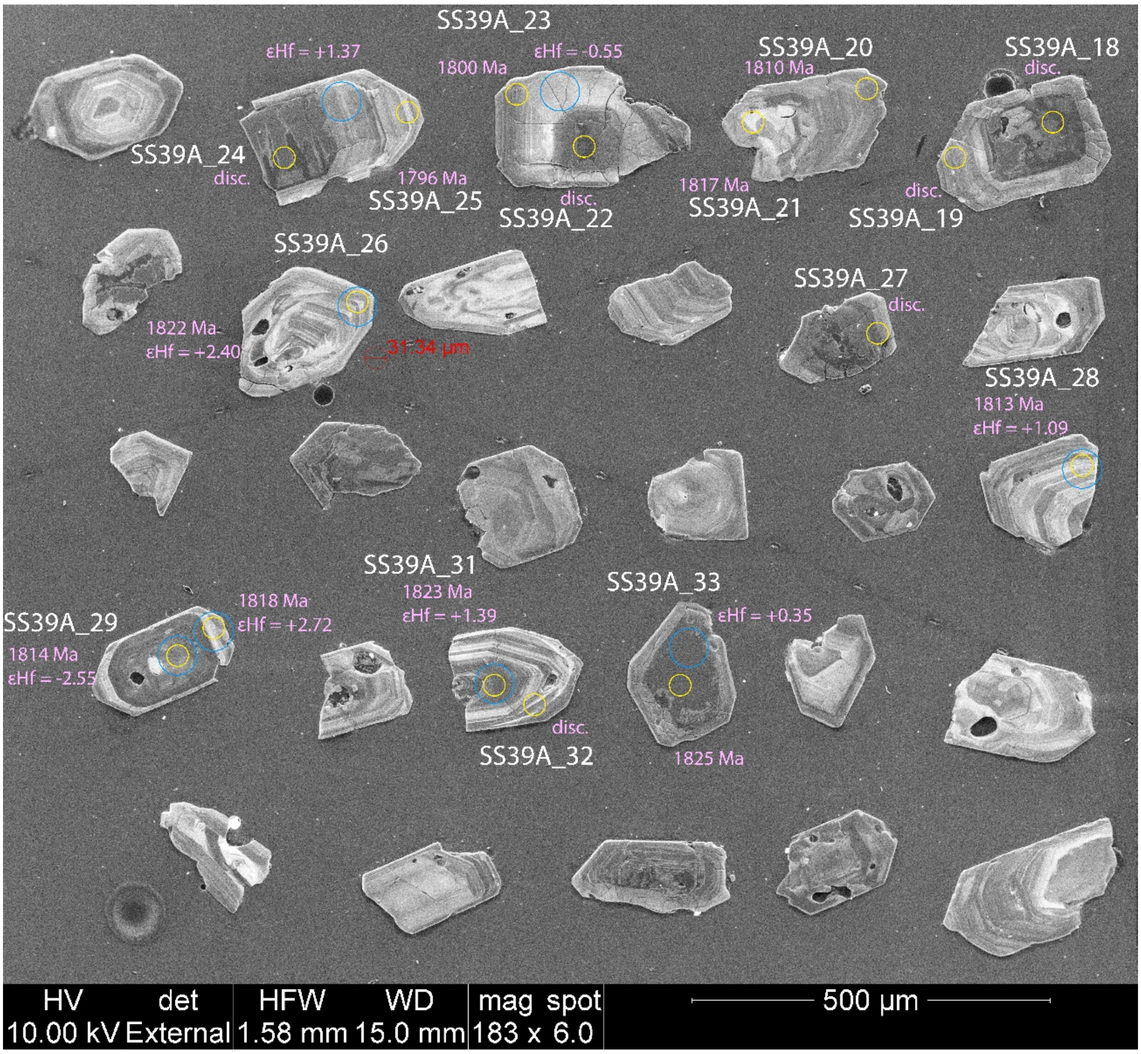
SS39A_23
εHf = +1.37
εHf = -0.55
1800 Ma
SS39A_20
1810 Ma
SS39A_18
disc.
SS39A_24
disc.
1796 Ma
SS39A_25
disc.
SS39A_22
1817 Ma
SS39A_21
disc.
SS39A_19
SS39A_26
1822 Ma
εHf = +2.40
31.34 µm
SS39A_27
disc.
SS39A_28
1813 Ma
εHf = +1.09
SS39A_31
1823 Ma
εHf = +1.39
SS39A_33
εHf = +0.35
SS39A_29
1818 Ma
εHf = +2.72
1814 Ma
εHf = -2.55
disc.
SS39A_32
1825 Ma
HV det HFW WD mag spot
10.00 kV External 1.58 mm 15.0 mm 183 x 6.0
500 µm

PB-07A

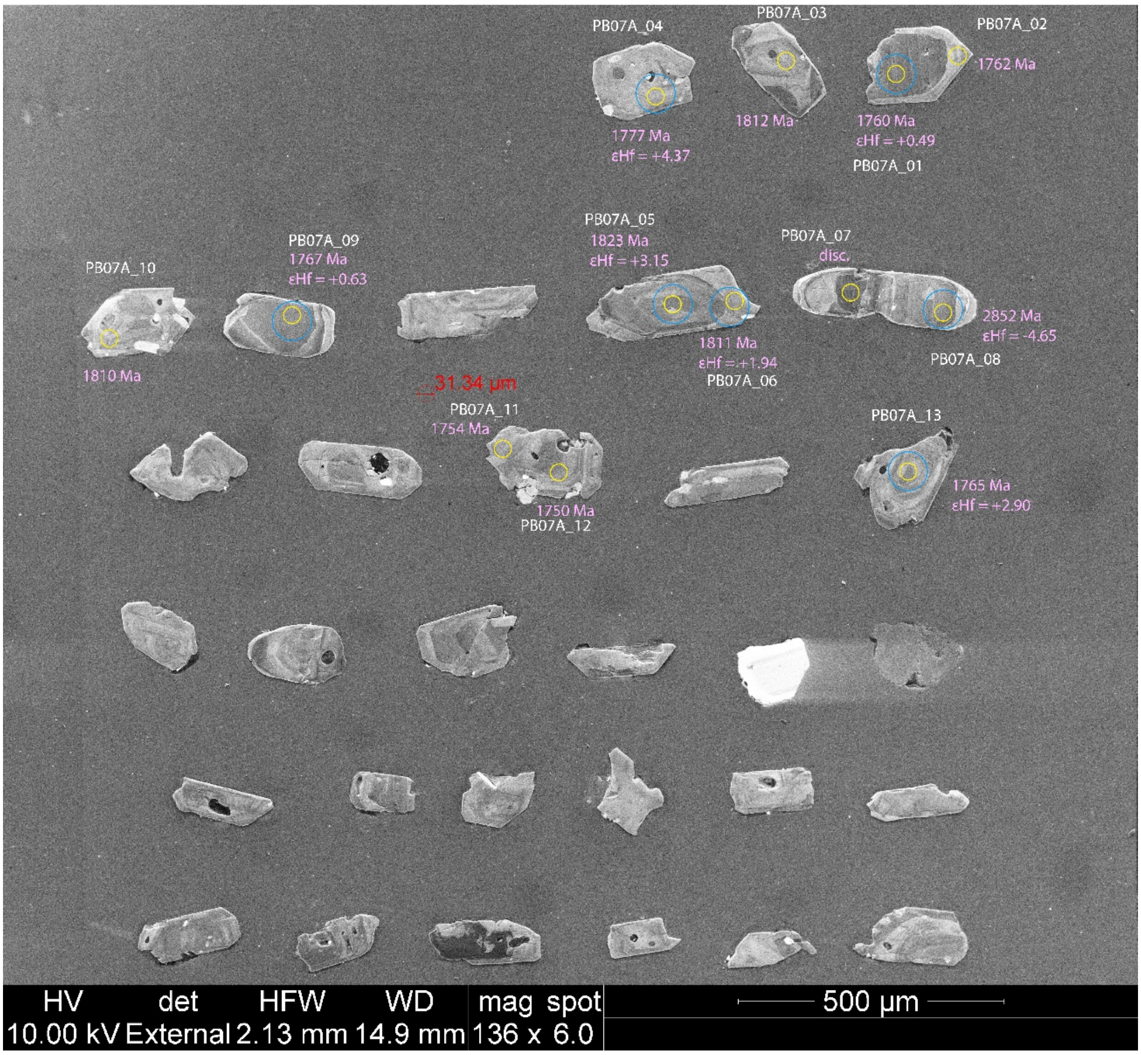
PB07A_04
PB07A_03
PB07A_02
1762 Ma
1777 Ma
εHf = +4.37
1812 Ma
1760 Ma
εHf = +0.49
PB07A_01
PB07A_05
1823 Ma
εHf = +3.15
PB07A_07
disc.
PB07A_09
1767 Ma
εHf = +0.63
PB07A_10
1810 Ma
1811 Ma
εHf = +1.94
PB07A_06
2852 Ma
εHf = -4.65
PB07A_08
31.34 μm
PB07A_11
1754 Ma
1750 Ma
PB07A_12
PB07A_13
1765 Ma
εHf = +2.90
HV
det
HFW
WD
mag
spot
10.00 kV
External
2.13 mm
14.9 mm
136 x
6.0
500 μm

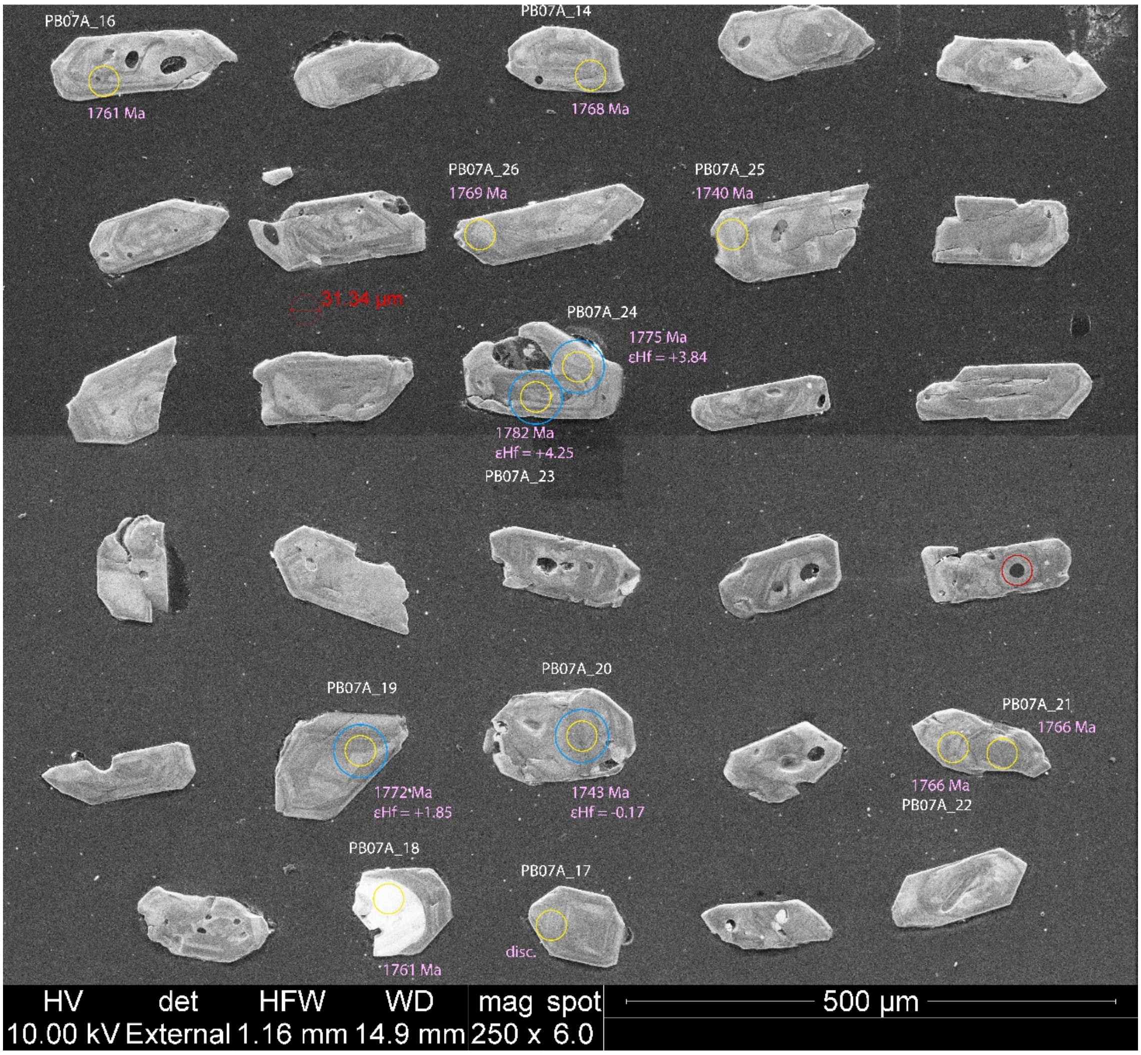
PB07A_16
1761 Ma
PB07A_14
1768 Ma
PB07A_26
1769 Ma
PB07A_25
1740 Ma
31.34 µm
PB07A_24
1775 Ma
εHf = +3.84
1782 Ma
εHf = +4.25
PB07A_23
PB07A_19
1772 Ma
εHf = +1.85
PB07A_20
1743 Ma
εHf = -0.17
PB07A_21
1766 Ma
1766 Ma
PB07A_22
PB07A_18
1761 Ma
PB07A_17
disc.
HV det HFW WD mag spot
10.00 kV External 1.16 mm 14.9 mm 250 x 6.0
500 µm

PB-09

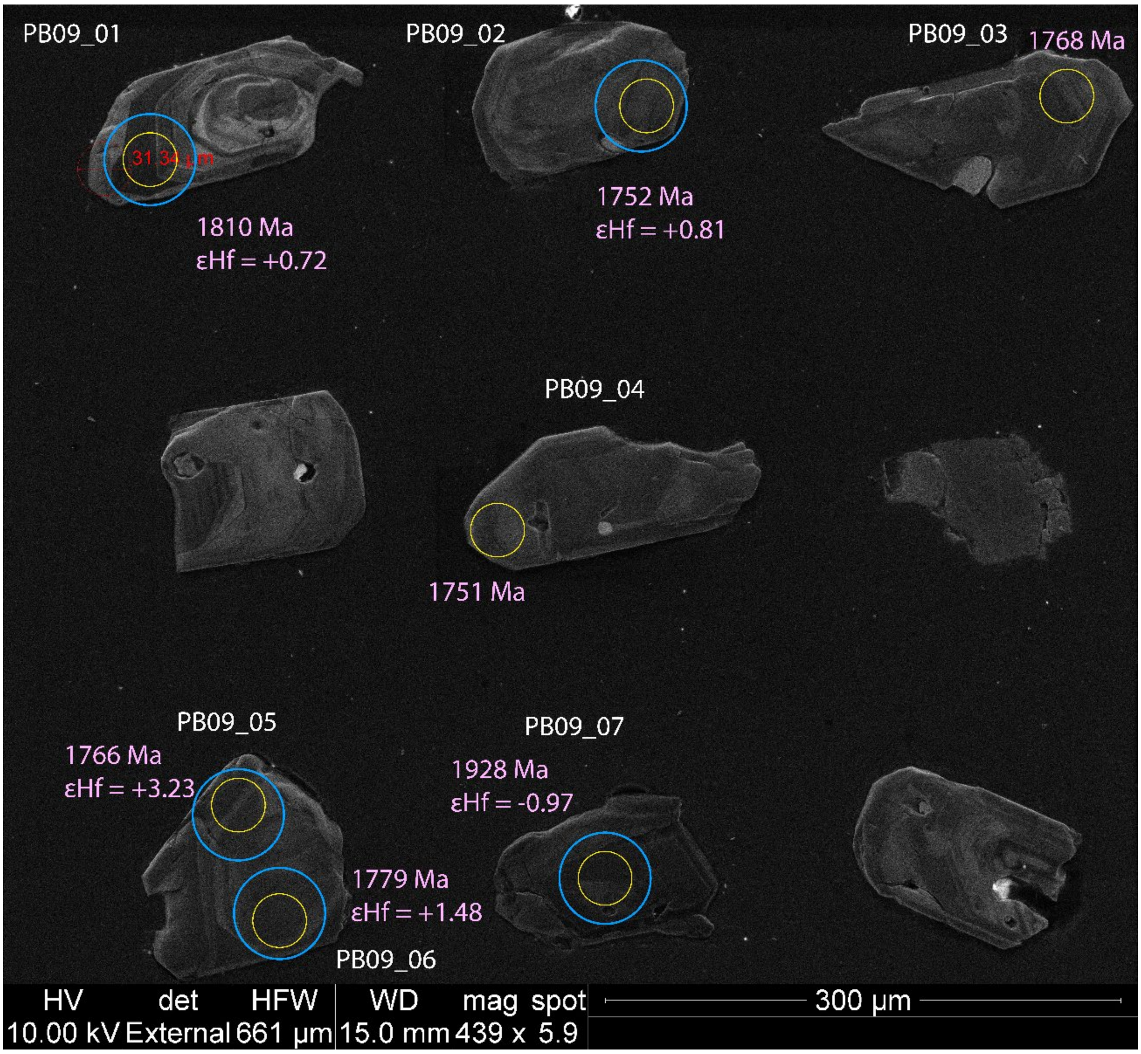
PB09_01
31.34 µm
1810 Ma
εHf = +0.72
PB09_02
1752 Ma
εHf = +0.81
PB09_03
1768 Ma
PB09_04
1751 Ma
PB09_05
1766 Ma
εHf = +3.23
1779 Ma
εHf = +1.48
PB09_06
PB09_07
1928 Ma
εHf = -0.97
HV det HFW WD mag spot
10.00 kV External 661 µm 15.0 mm 439 x 5.9
300 µm

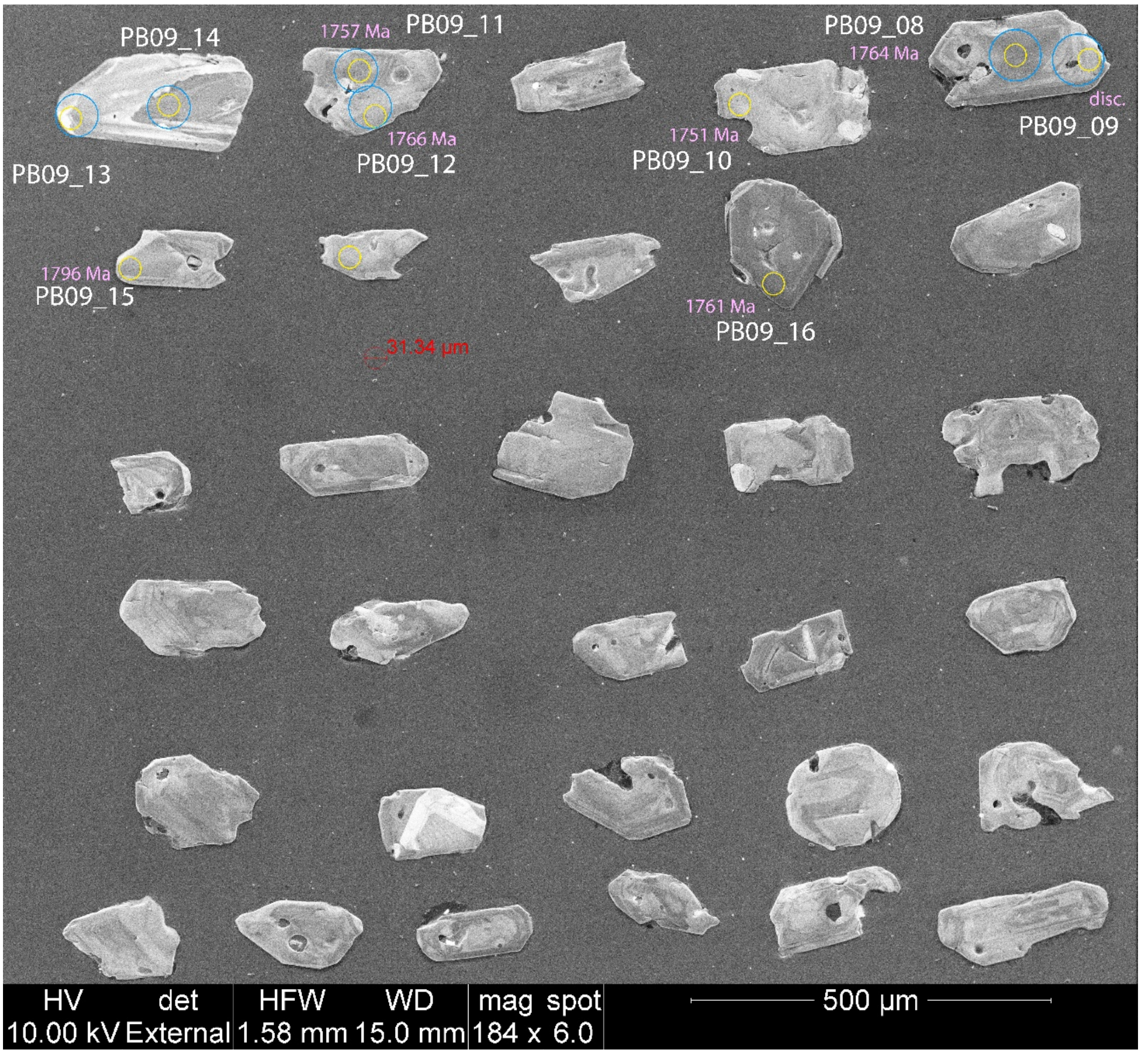
PB09_14
1757 Ma
PB09_11
PB09_08
1764 Ma
disc.
PB09_09
1766 Ma
PB09_12
1751 Ma
PB09_10
PB09_13
1796 Ma
PB09_15
1761 Ma
PB09_16
31.34 µm
HV
det
HFW
WD
mag
spot
10.00 kV
External
1.58 mm
15.0 mm
184 x
6.0
500 µm

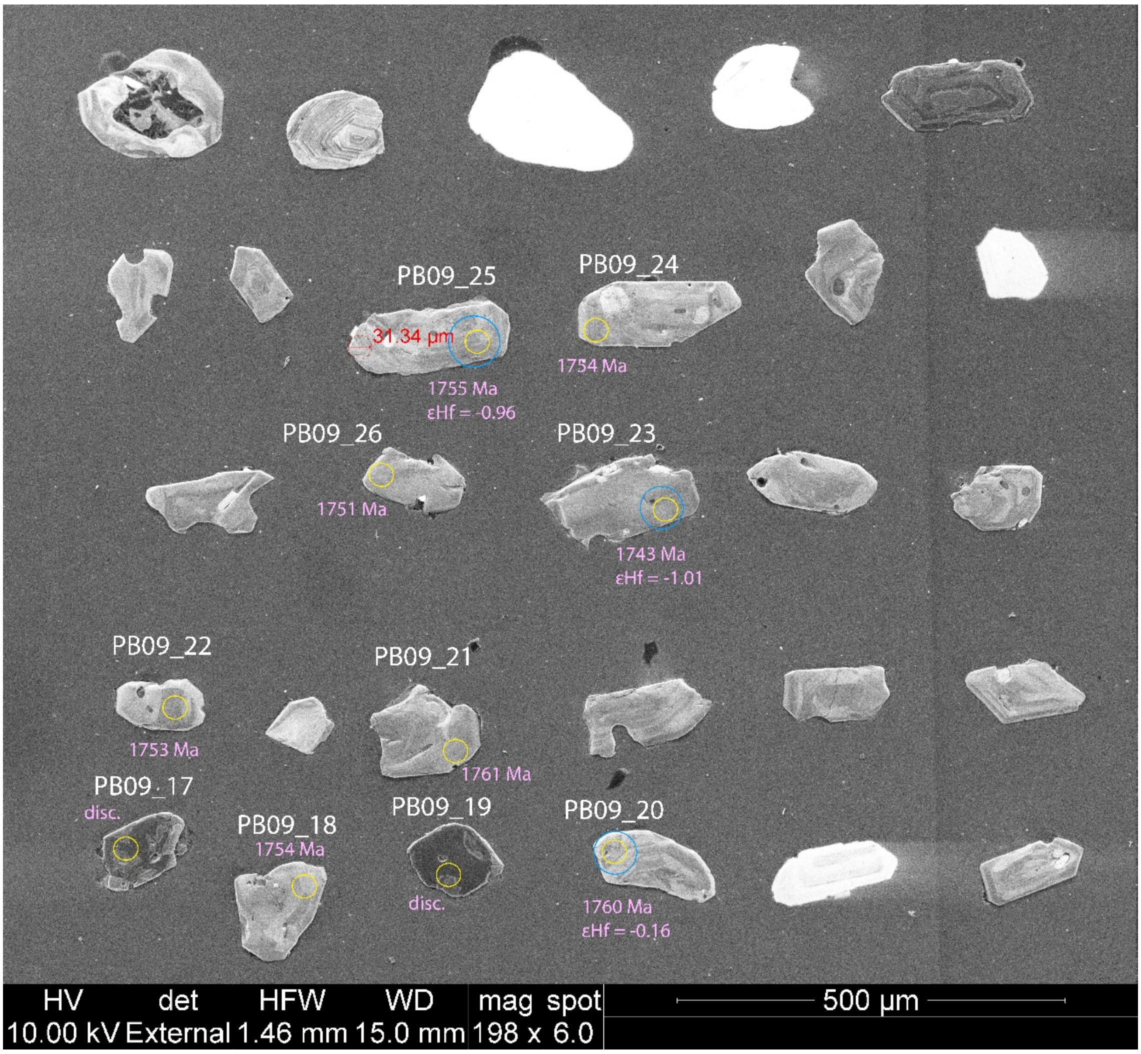
PB09_25
31.34 µm
1755 Ma
εHf = -0.96
PB09_24
1754 Ma
PB09_26
1751 Ma
PB09_23
1743 Ma
εHf = -1.01
PB09_22
1753 Ma
PB09_21
1761 Ma
PB09_17
disc.
PB09_18
1754 Ma
PB09_19
disc.
PB09_20
1760 Ma
εHf = -0.16
HV
det
HFW
WD
mag
spot
10.00 kV
External
1.46 mm
15.0 mm
198 x
6.0
500 µm
