# Supplementary material for: Large-scale crustal growth driven by LIP magmatism during the Paleoproterozoic
Source: Nat Commun. 2025 Nov 28;16:10779. doi: 10.1038/s41467-025-65826-5 (PMC12663317; doi:10.1038/s41467-025-65826-5)
Supplement: Supplementary file 7 — Supplementary Data 6 [file 41467_2025_65826_MOESM7_ESM.pdf]

Supplementary Data 6

Large-scale crustal growth driven by LIP magmatism during the Paleoproterozoic

Trace-element spidergrams and rare-earth element (REE) diagrams illustrating zircon trace-element compositions of individual samples

Matheus S. Simões, Andrew R.C. Kylander-Clark, Marcelo L. Vasquez, Carlos A. Sommer, Lucas M.M. Rossetti, John M. Cottle, Túlio A. Mendes

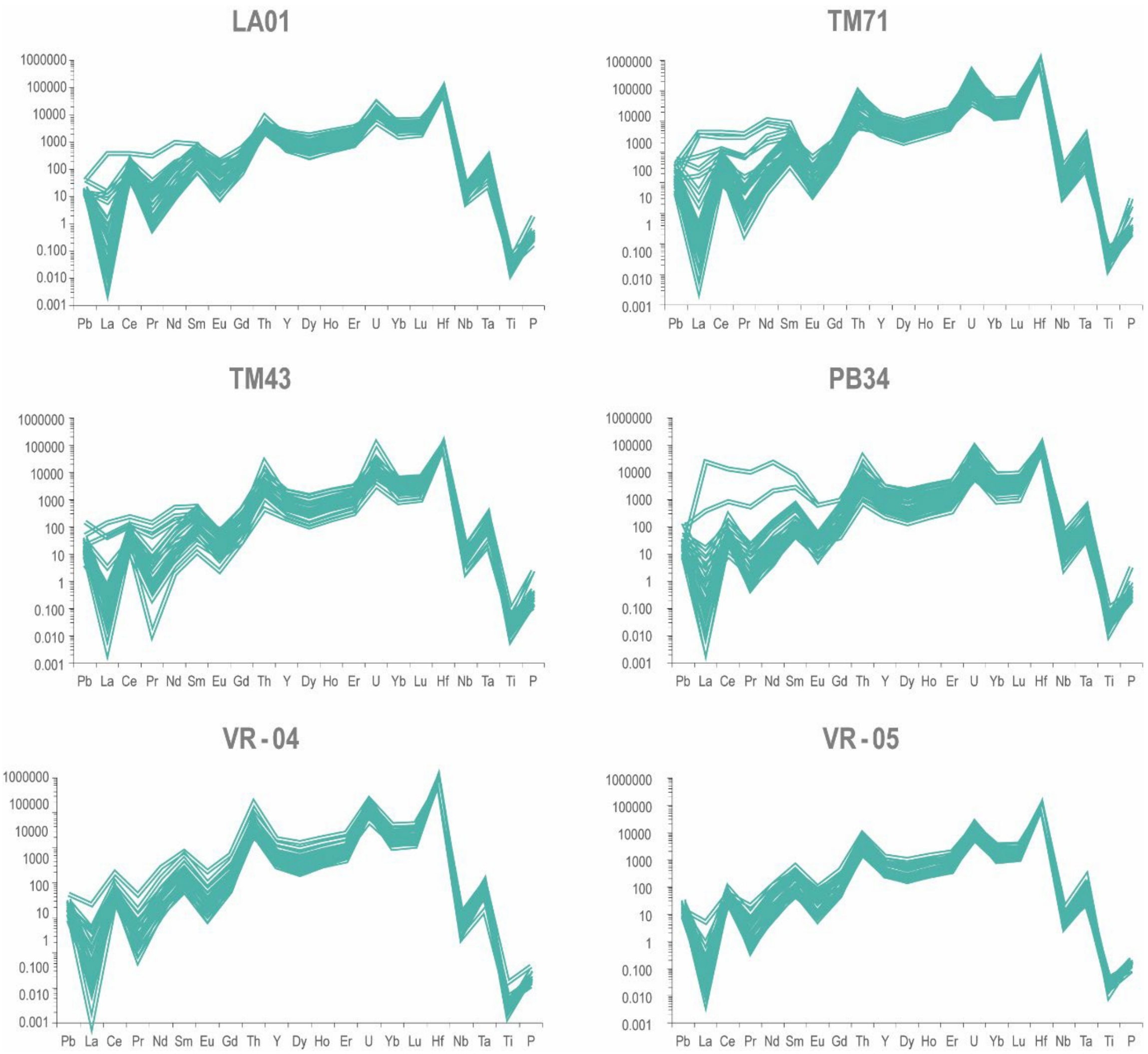

Figure 1 – Chondrite-normalized[1] zircon trace-element patterns for samples of Cycle 1.

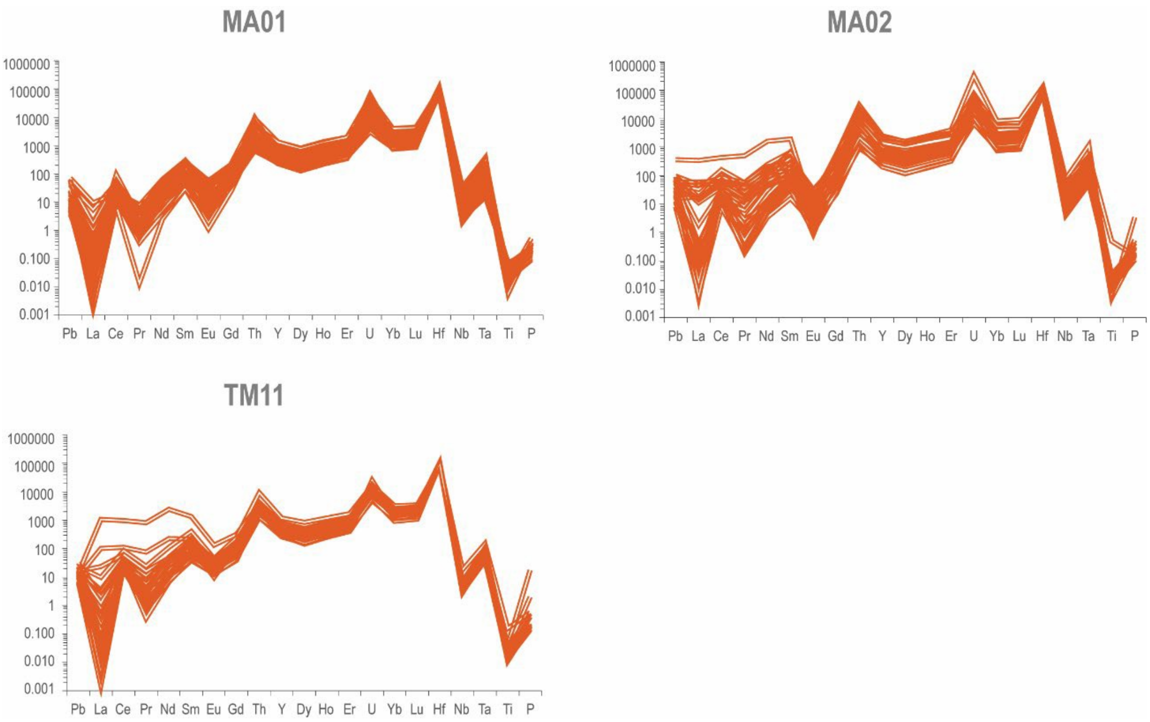

Figure 2 – Chondrite-normalized[1] zircon trace-element patterns for samples of Cycle 2.

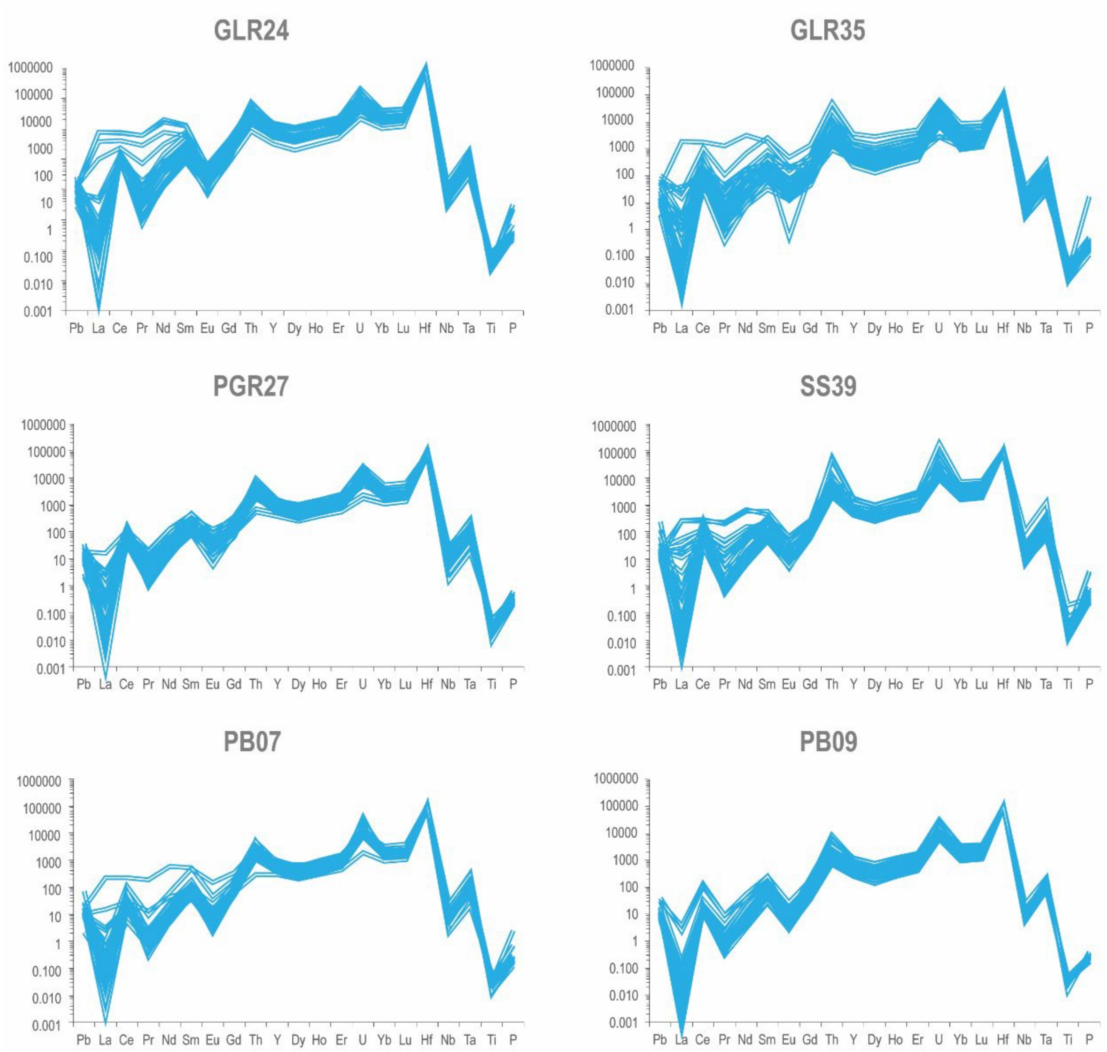

Supplementary Figure 3 – Chondrite-normalized[1] zircon trace-element patterns for samples of Cycle 3.

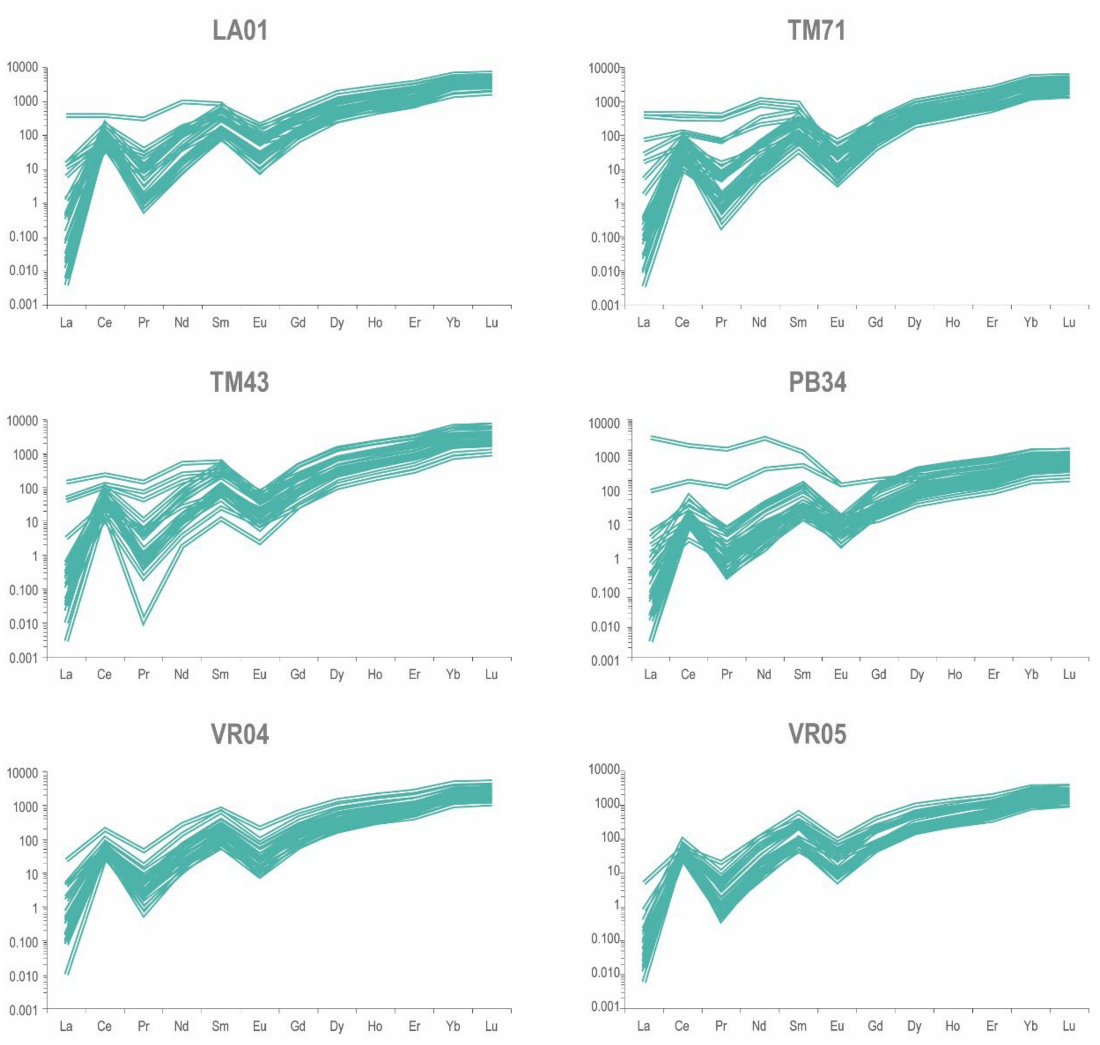

Figure 4 – Chondrite-normalized[1] zircon rare-earth element patterns forsamples of Cycle 1.

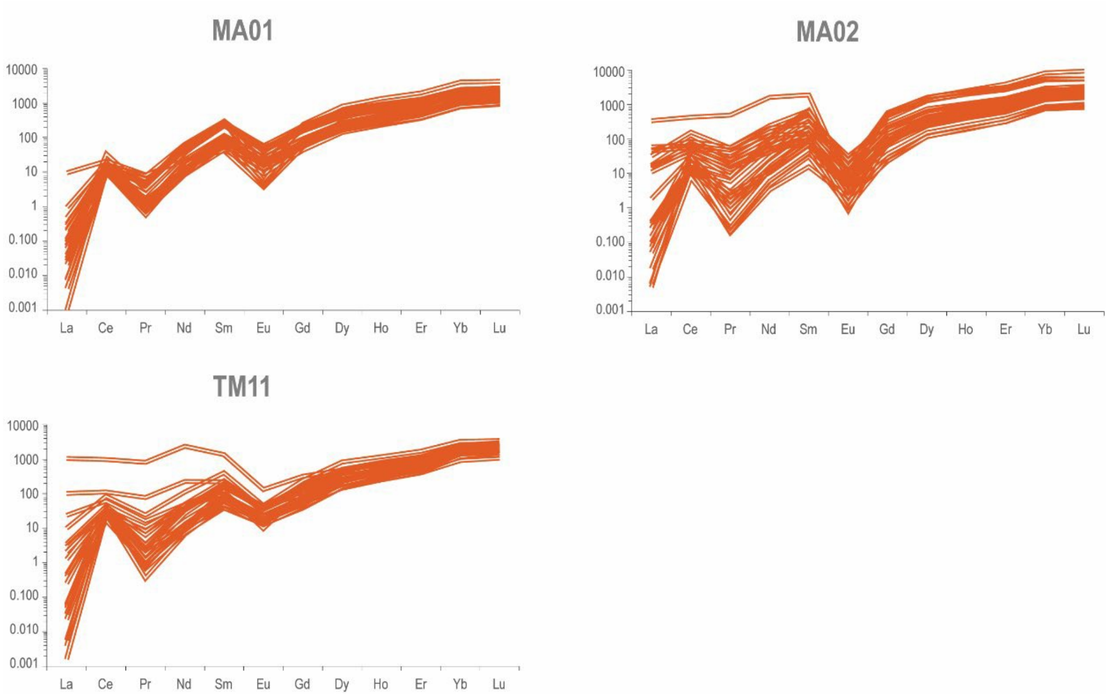

Figure 5 – Chondrite-normalized[1] zircon rare-earth element patterns forsamples of Cycle 2.

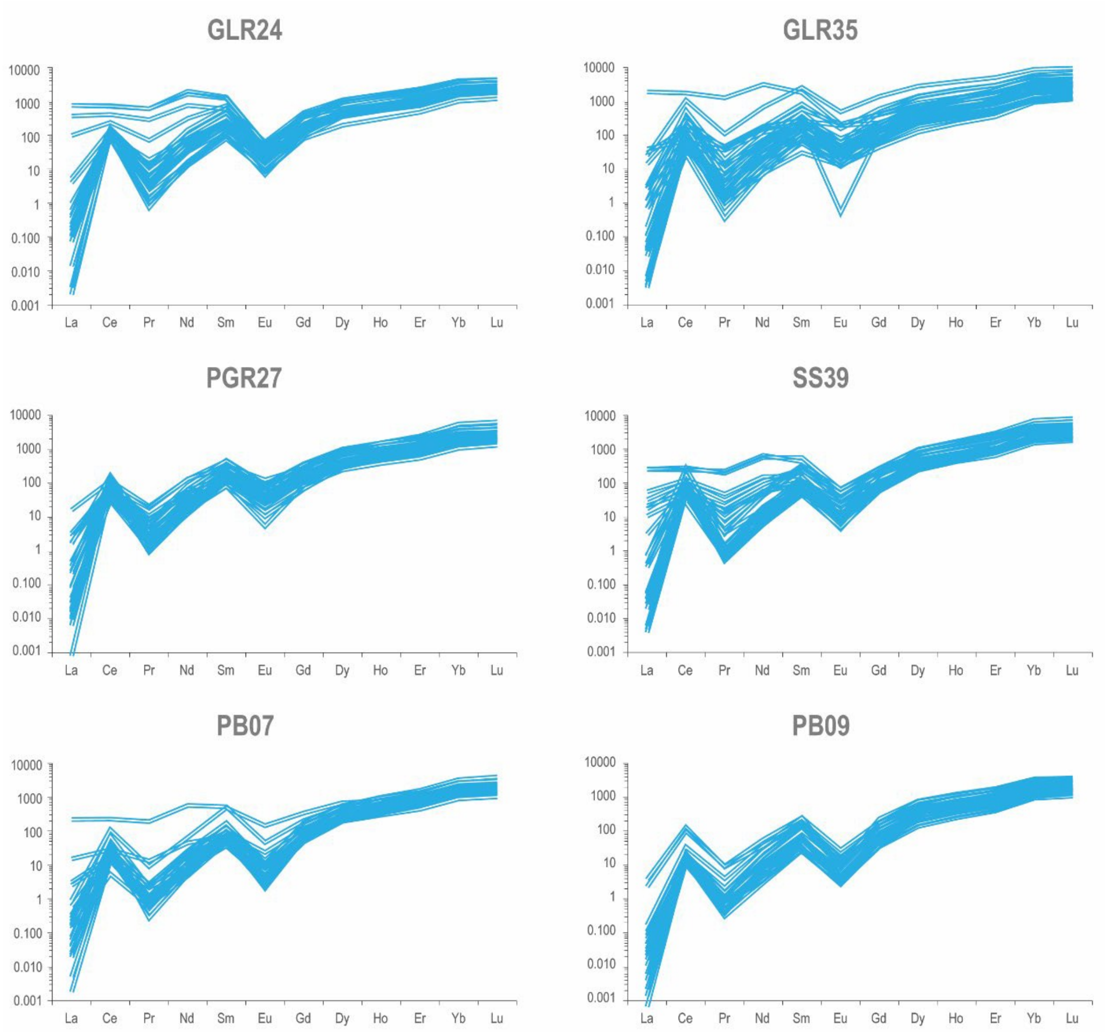

Figure 6 – Chondrite-normalized[1] zircon rare-earth element patterns forsamples of Cycle 3.

1. Sun, S.-S., McDonough, W.F. Chemical and isotopic systematics of oceanic basalts: implications for mantle composition and processes, in: Saunders, A.D., Norry, M.J. (Eds.), *Magmatism in the Ocean Basin*. Geol Soc Sp, London, **42**, 313-345 (1989)
