# Supplementary material for: Large-scale crustal growth driven by LIP magmatism during the Paleoproterozoic
Source: Nat Commun. 2025 Nov 28;16:10779. doi: 10.1038/s41467-025-65826-5 (PMC12663317; doi:10.1038/s41467-025-65826-5)

Supplementary Data for

Large-scale crustal growth driven by LIP magmatism during the Paleoproterozoic

Matheus S. Simões, Andrew R.C. Kylander-Clark, Marcelo L. Vasquez, Carlos A. Sommer, Lucas M.M. Rossetti, John M. Cottle, Túlio A. Mendes, Andrew R.C. Kylander-Clark

Chemical mohometry results based on

Luffi, P. & Ducea, M. N. Chemical mohometry: assessing crustal thickness of ancient orogens using geochemical and isotopic data. *Rev. Geophys.* **60**, e2021RG000753 (2022).

Supplementary Figure 7 – Chemical mohometry for CYCLE 1

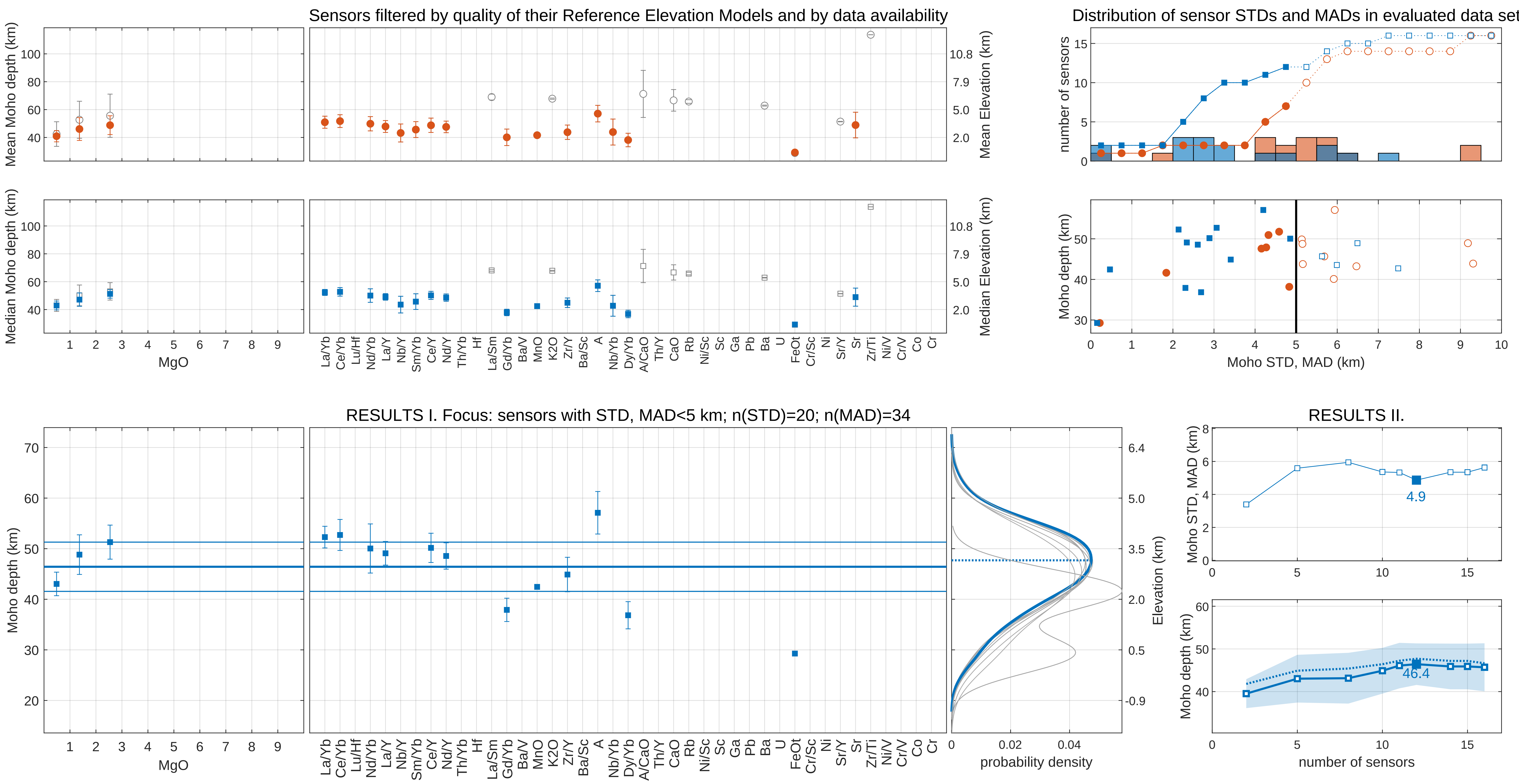

Supplementary Figure 8 – Chemical mohometry for **CYCLE 2**

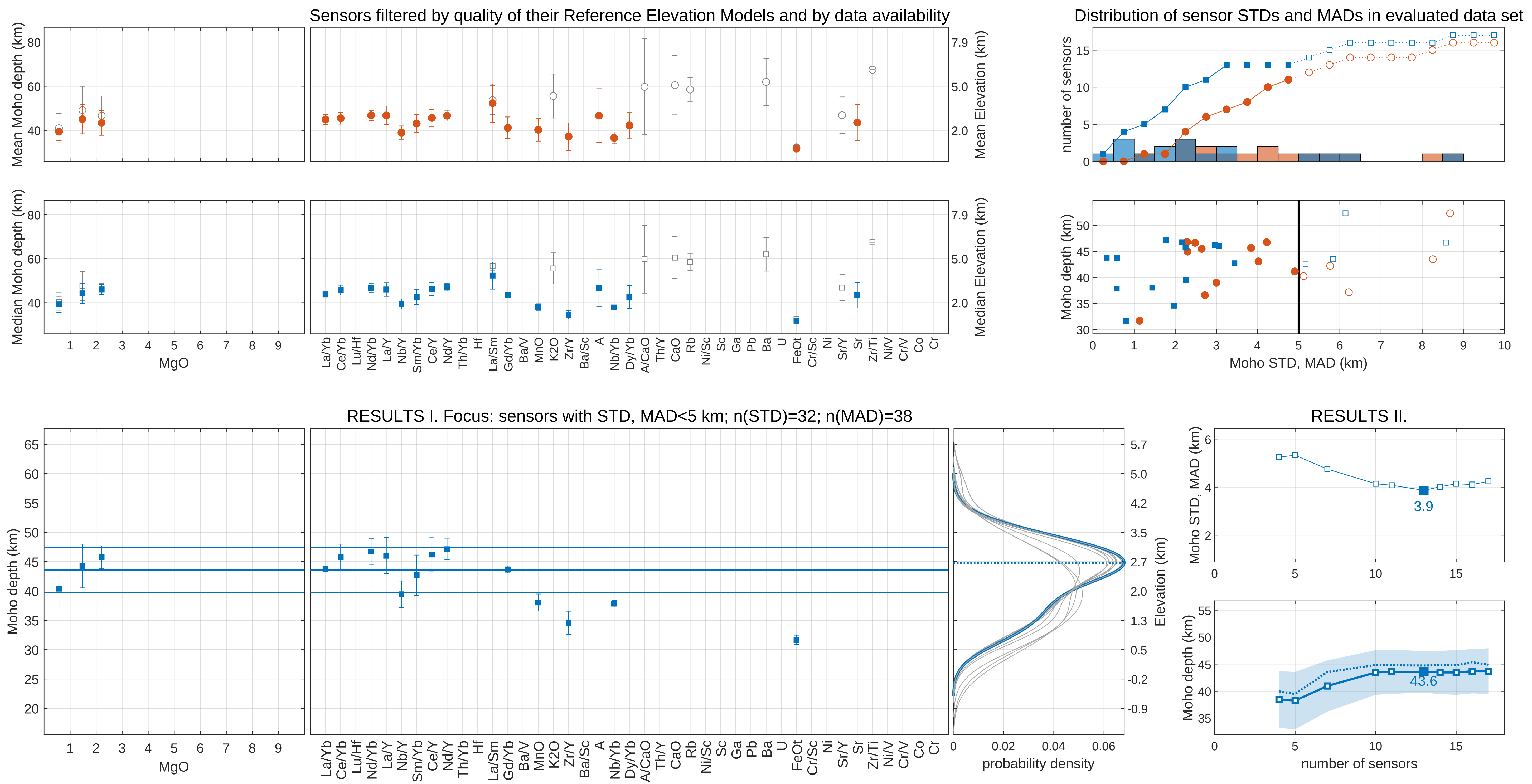

Supplementary Figure 9 – Chemical mohometry for **CYCLE 3**

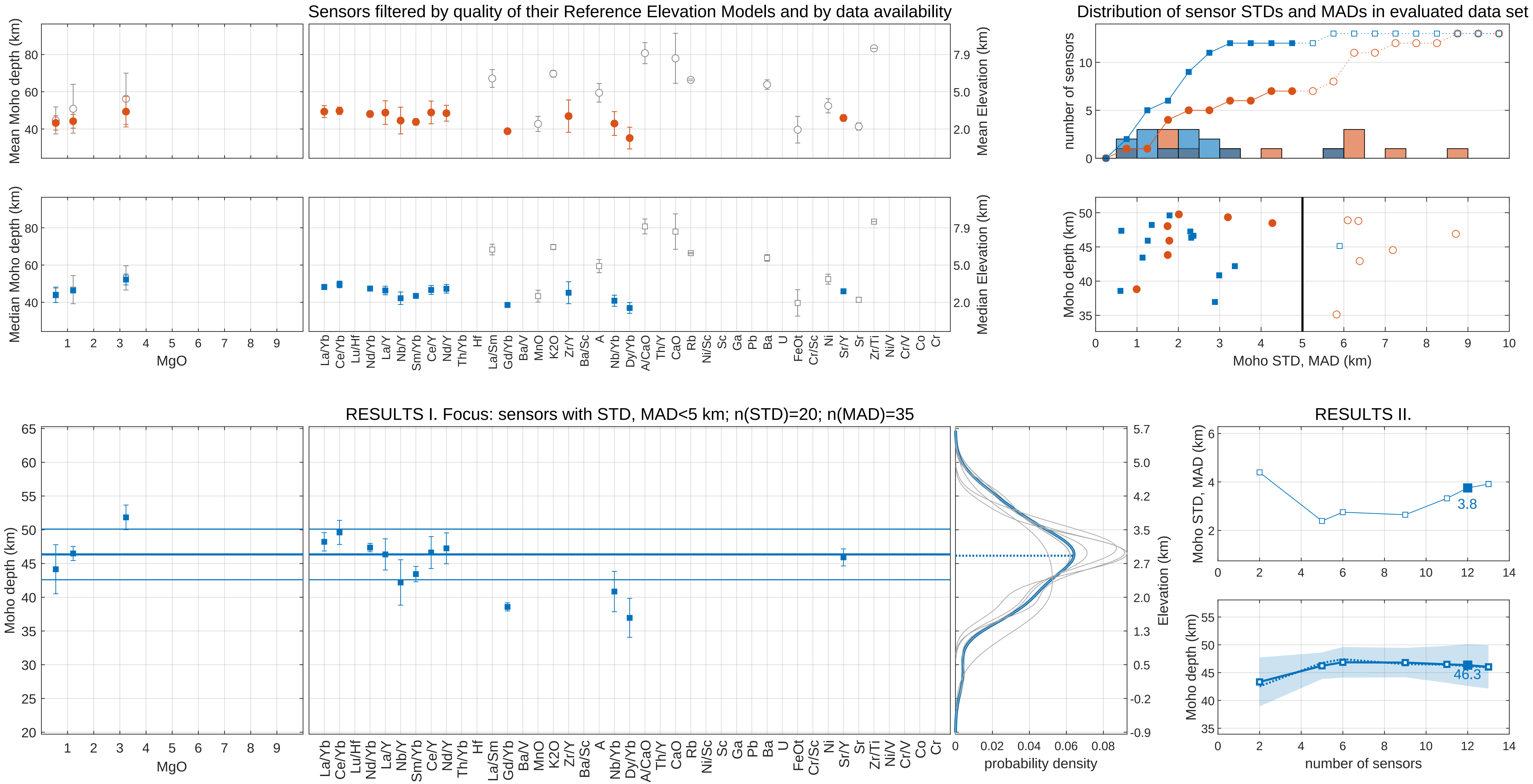

Supplement: Supplementary file 8 — Supplementary Data 7 [file 41467_2025_65826_MOESM8_ESM.pdf]
